# Ubiquitous Occurrence of Nano Selenium in Food Plants

**DOI:** 10.3390/foods12173203

**Published:** 2023-08-25

**Authors:** Jonas Verstegen, Klaus Günther

**Affiliations:** 1 Institute of Nutritional and Food Sciences, University of Bonn, 53115 Bonn, Germany; 2 Federal Institute for Drugs and Medical Devices, 53175 Bonn, Germany; 3 Research Centre Juelich, Institute of Bio- and Geosciences, IBG-2, Plant Sciences, 52428 Jülich, Germany

**Keywords:** selenium, nanoparticles, sp-ICP-MS, biosynthesis, plant, nutrition, crop science

## Abstract

Selenium is an essential trace element in human nutrition. Recent findings suggest that the biosynthesis of selenium nano particles (SeNPs) in plants might be a ubiquitous phenomenon. We investigated the potential of SeNP biosynthesis in food plants and our core objective was to explore the commonness and possible ubiquitousness of nano selenium in food plants and consequently in the human diet. By growing a variety of plants in controlled conditions and the presence of selenite we found strong evidence that SeNPs are widely present in vegetables. The shoots and roots of seven different plants, and additionally Brazil nuts, were analyzed with single-particle inductively coupled plasma mass spectrometry with a focus on edible plants including herbs and salads. SeNPs were found in every plant of our study, hence we conclude, that SeNPs are common ingredients in plant-based food and are therefore eaten daily by most humans. Considering the concerning worldwide prevalence of selenium deficiency and the great physiological properties of SeNPs, we see a high potential in utilizing this discovery.

## 1. Introduction

Selenium is an essential trace element with great importance for human health. The oxidative states it occurs in are similar to sulfur-II to +VI and accordingly, selenium can be found in corresponding compounds, including selenide (Se^2−^), selenite (SeO_3_^2−^), and selenate (SeO_4_^2−^). The two latter ones, alongside organic compounds, namely selenomethionine (SeMet) and selenocysteine (SeCys), are well-described forms of selenium in human nutrition. The European Food Safety Authority (EFSA) recommends a daily intake of 70 μg/day for adult men and women and a progressive weight dependent amount between 15 μg/day (for ages 1 to 3 years and a reference body weight of 11.9 kg) and 55 μg/day (for ages 11 to 14 years and a reference body weight of 45.7 kg) for children. An adjusted intake during pregnancy is not recommended. Yet, the average daily intake is estimated by the EFSA to be between 31.0 and 65.6 μg/day in adults (≥18 years) and 20.6 to 45.9 μg/day in children aged 3 to <10 years [1].

Intestinal absorption of selenium is generally good, particularly in comparison to other micronutrients, for example zinc (25% from milk and dairy food [2]) and iron (14 to 18% from mixed diets and 5 to 12% from vegetarian diets [3]). Absorption from selenite was found to be between 62 and 76% and above 90% for selenomethionine and selenate. The absorption of selenium in an unknown chemical state from food was found to be 83%, as tested on an intake of 100 g shrimp/day [4].

Selenium rich foods are mainly animal-based, they include fish, shellfish, and meat. Often in agriculture, fodder is enriched with selenium [5]. Plant-based food can be a less reliable source of selenium, as the selenium intake of plants is dependent on salinity, pH-levels, and soil composition, including the strong variation of the selenium content in soil [1,6]. The reliability of plants as selenium sources can be increased by using selenium-enriched fertilizer [7]. While small amounts of selenium can show beneficial effects on resistance against stress factors like heat and drought, it is not considered an essential element for plants [8,9], and with few exceptions, only small amounts of selenium are tolerable for plants. Plant species can be divided into three groups, selenium non-accumulators, selenium accumulators, and selenium hyperaccumulators and concentrations of <100 mg/kg, 100–1000 mg/kg, or >1000 mg/kg dry weight can be found in those plants, given that there is sufficient selenium available in the soil [8,10].

A total of 25 human selenoproteins have been identified so far and play important roles in human physiology. Their functions include key roles in the immune system and cancer prevention, thyroid metabolism, brain function, fertility, and antioxidant processes [11,12,13]. Selenium deficiency is associated with cardiovascular disease, cancer, liver disease, osteoarthritis, and Keshan disease [14,15,16,17,18]. Selenium impacts apoptosis and autophagy in cardiomyocytes, therefore a deficiency cannot only cause Keshan disease, but other forms of cardiomyopathy as well [17]. The antiviral properties of selenium are especially promising in nano particles. Zanamivir-loaded SeNPs showed superior cell viability than zanamivir or SeNP alone and selenium is also associated with good outcomes in COVID-19 infections [19,20].

Due to its heavier and larger nature, selenium in the form of SeCys can be superior to sulfur and Cys in enzymes, as it is both a good electrophile and nucleophile. Sulfur is less polarizable than selenium and can therefore not regenerate to its active form within the catalytic cycle as easily and quickly [9].

Even for a micronutrient, the therapeutic range of selenium is noteworthily narrow, therefore food sources and food supplements containing selenium require to be of high quality with a minimized danger of toxicity. Thus, recommendations for selenium supplementation should be made with careful consideration for the soil of the respective area and the selenium supply due to food. Toxic effects of selenium mostly occur in protein biosynthesis where SeCys and SeMet might mistakenly be put in the place of Cys or Met [21]. This replacement can happen non-specifically and subsequently cause faulty proteins. Due to the important role of Cys in the structure and function of many proteins, it being replaced by SeCys bears a greater danger than Met being replaced by SeMet. SeNPs excel in that matter, since despite their great bioavailability and activity, their toxicity is lower than that of other selenium compounds [13,22,23,24,25,26].

Among the most promising applications for SeNPs are treatments for various kinds of cancer. An optimal selenium supply can decrease the incidence of cancer. Mostly its antioxidant nature and ability to control reactive oxygen species (ROS) and radicals factor into its chemopreventive nature. Decreased levels of selenium and seleno-enzymes, like glutathione peroxidase (GPx) for example, are associated with malignant melanoma, colorectal cancer, lung cancer, hepatocellular carcinoma, and gallbladder and biliary tract cancers [14,18,26,27]. However, increased levels of selenium can increase the risk of cancer as well. This double-edged relationship between selenium intake and cancer is particularly well described for prostate cancer [12,28,29].

SeNPs are superior to other forms of selenium as food supplements, due to their high bioavailability, low toxicity, and high biological activity [13]. Additionally, SeNPs were shown to be present in an elemental state and the Se^0^ is acting particularly well at protecting against heavy-metal toxicity [13,30]. This is achieved partly by direct reductive activity, but also by the induction of several pathways and enzymes like Nrf2, GSH, and hemoglobin oxygenase.

A modification of SeNPs can be performed and a conjugation with another agent that can, for example, be used to specifically target the tumor cells can enhance the antitumor activity while lowering its toxicity [24]. Successful applications of SeNPs include trials with estrogen receptor α-positive breast cancer cells, colon cancer cells, prostate cancer cells, and cervical carcinoma cells [24,26,31,32,33]. The size effect of SeNPs and nano particles in general is still discussed. While some researchers find inconclusive results, some studies find that the activities of GPx, thioredoxin reductase, and other selenium dependent enzymes increase more when a given amount of SeNP is applied with smaller particles [34,35]. It is therefore of great interest to the nutritional and medicinal science community to find ways to produce selenium in nano form with a small size and a narrow size distribution.

Recently, we found evidence that plants are able to naturally produce SeNP when exposed to selenite [26]. This was found for species from many different orders of plants and might turn out to be a ubiquitous phenomenon. Generally, selenium is not an essential nutrient for plants and so far, no genes that encode for SeCys or SeMet were found in a plant genome [8,9]. For most plants, selenium is mainly a potential toxin, potentially causing oxidative and nitrosative stress. Additionally, as in animals, seleno amino acids might be confused with their sulfuric equivalents and cause faulty proteins in plants as well [8,26,36]. Selenium can on the other hand still cause positive effects for plant organisms in small doses, like resistance to stress from factors like metallic stress and drought [10]. Furthermore, selenium can enhance plant growth and crop yield [37,38,39].

Selenium has a high bioavailability for organic and inorganic compounds. The uptake of selenate and selenite mostly happens through sulfate and phosphate transporters, respectively [6,40]. Many of the earlier described selenium hyperaccumulators belong to the family of Brassicaceae. Various food plants, namely cruciferous vegetables, belong to that family. In this research, it was our aim to investigate the potential synthesis of SeNPs in food plants and potentially find evidence that nano selenium is already part of many humans’ diets. As a continuation of our previous work, we grew plants under controlled conditions in the presence of selenite and analyzed their SeNP content with single-particle mass spectrometry with inductively coupled plasma (sp-ICP-MS). It was our aim to quantify the amount of nano selenium and find insight into their size and size distribution and thus on the presence of nano selenium in food.

## 2. Materials and Methods

### 2.1. Chemicals and Instruments

A Sartorius arium^®^ pro system was used to produce ultrapure water (18.2 MΩ·cm). A Qiagen Tissue Ruptor II was used to homogenize the plant samples. Macerozyme R-10 derived from *Rhizupus* sp. and Proteinase K were used for plant digestion. They were purchased from GeneON and bioWorld, respectively. For the buffer preparation, disodium hydrogen citrate and citric acid were purchase from Sigma-Aldrich. Dialysis membrane Spectra/Por^®^ 3 with a MWCO of 3.5 kDa and the corresponding closures were purchased from Fisher Scientific.

For the plant growth, a hydroponic system by growland was used. Hoagland solution was prepared from a salt base that was purchased from Biozol. Sodium selenite to spike the growth solution and formalin solution to surface sterilize the seeds were purchased from Merck. Ethanol 96% that was used during the dialysis was purchased from Sigma-Aldrich.

A NexION 350D sp-ICP-MS by Perkin Elmer (Waltham, MA, USA) was used for the analysis. It was equipped with a quartz cyclonic spray chamber and a glass nebulizer (Ar 1.0 SLPM @ 43 psi). The peristaltic pump tubing with flared ends and an inner diameter of 0.38 mm was made of polyvinyl chloride.

Polypropylene tubes with a volume of 50 mL were purchased from Sarstedt AG & Co.KG (Nümbrecht, Germany) and tubes with a volume of 15 mL were purchased from Cellstar.

### 2.2. Plant Treatment

Basil (*Ocinum basilicum*), dill (*Anethum graveolens*), chard (*Beta vulgaris*), spinach (*Spinacia oleracea*), brussels sprouts (*Brassica oleracea var. gemmifera*), broccoli (*Brassica oleracea var. italica*), lamb’s lettuce (*Valerianella locusta*), and Brazil nuts (*Bertholletia excelsa*) were used in our research.

The plant treatment was performed in the same way as in our previous work on SeNPs in plants [26]. Formalin solution with a 10% concentration was used to perform a surface sterilization on the seeds for 10 min. Ultrapure water was used to rinse of any remaining formalin. The seeds were transferred onto filter paper that was kept moist and dark in order to allow germination for up to 7 days. After germination, the seedlings were conveyed to a hydroponic system, where they were grown for 28 to 42 days. Hoagland solution was used as growth solution, after it was diluted to quarter strength and was spiked with 5 mg/L sodium selenite, resulting in a concentration von 28.91 µmol/L of selenium.

Water absorption by the plants and evaporation was compensated every 2 to 3 days. Two neon tubes with a power of 55 W and a color temperature of 6500 K and a reflector were placed at a distance of 30 cm above the seeds. The plants were grown in 8 h of darkness and 16 h of artificial light. The light period started at 6 a.m. and ended at 10 p.m. After the growth period, the plants were harvested from the hydroponic system and the shoots were separated from the roots. Intermixing was avoided by disposing of the root crowns. Both tissues were rinsed with DI water.

The root surface was further cleaned. After a second rinse with DI water, rough cuts of the root tissue were prepared and subsequently transferred to a pH 6 M citrate buffer (0.1 M). The roots were stirred for 48 h to rinse off any residues of growth solution. The buffer solution was then rinsed off with DI water and a sample of 100 mg was taken from the tissue. Without the 48 h period, the shoots were treated in the same manner and samples of 100 mg were taken. A tissue ruptor was used to homogenize the samples in 8 mL of the earlier-described citrate buffer for 2 min.

A total of 2 mL of a 50 mg/mL solution of Macerozyme R-10 was added to the mixture as well as 50 µL of a 20 mg/mL solution of Proteinase K. The mixture was shaken at 37 °C. After 24 h, 0.500 mL of ethanol was mixed with 0.500 mL of supernatant and 4.000 mL of earlier-described citrate buffer. A dialysis was performed in 450 mL of buffer and 50 mL of ethanol. The dialysis tubing had a 3.5 kDa MWCO and after being filled with the 5 mL sample, it was stirred for 24 h.

The sample was removed from the tubing afterwards and if necessary, citrate buffer was added to a volume of 5 mL. The mixture was diluted by a factor of 10 with ultrapure water before being analyzed.

For every species, three individual plants were used and treated as described. If a concentration higher than 1 µg/L of dissolved selenium was found in a sample, the last dilution step was not performed with a factor of 10, but instead 50 µL of ethanol was added to 500 µL of sample, which was then diluted with ultrapure water to 10 mL, resulting in a dilution factor of 20 instead of 10. Sample 1 and Sample 2 of chard root required this treatment.

The Brazil nuts were cut into small pieces and samples of 100 mg were treated in the same manner as the root and shoot tissues. Two different brands of Brazil nuts were purchased from two different supermarkets and since they contained very different amounts of selenium, the second batch of Brazil nuts was analyzed without the last dilution step (1000 µg/mL) while the first batch was diluted to 50 mL in the last sample preparation step resulting in a concentration of 10 µg/mL.

### 2.3. ICP-MS Method and Parameters

A method was developed using the Syngistix software version 2.4 with nano application, measuring the 80Se isotope with a relative abundance of 49.61%. Argon-dimer interference was removed using hydrogen in a dynamic reaction cell (DRC). The dwell time was set to 50 µs and measuring time to 120 s, while the settling time was eliminated completely. The sample flow rate was determined daily. Further instrumental parameters included:
RF power1300 WPlasma Ar flow 15 L/minReaction cell gas flow (H2)4.4 mL/minRPq0.8 VDeflector Attractor−135 VDeflector Entrance Lens−50 VTransport Efficiency7.26%

## 3. Results

**Figure 1:**
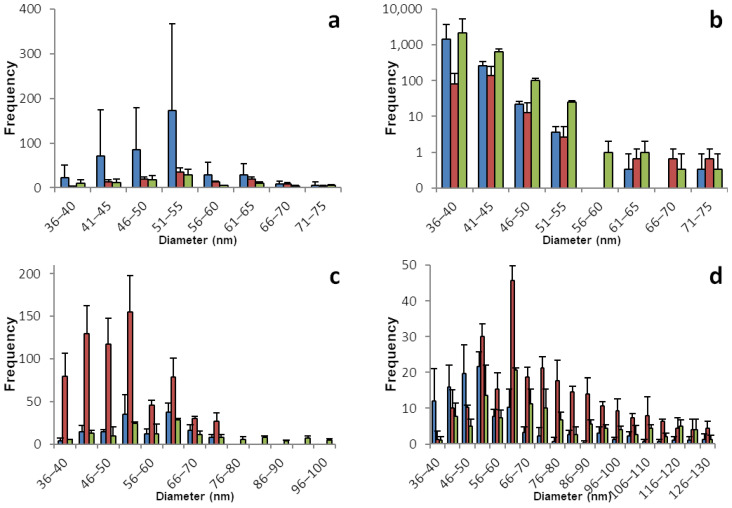
Size distribution of SeNPs in root and shoot tissues of basil and dill plants. These histograms show the sp-ICP-MS analysis of (**a**) basil root, (**b**) basil shoot, (**c**) dill root, and (**d**) dill shoot. Three specimens of each plant were grown, and each bar color represents a single plant. The standard deviation depicted describes the variation for 3 replicates of the same sample. Nano particles with a size of up to 531 nm were detected, however the histograms in this figure are meant to show the main distribution of SeNPs and were therefore cropped. Supplementary information S1 shows the full results. Supplementary information S2 shows histograms including all detected data points for the individual replicates.

The sp-ICP-MS analyses for basil and dill plants are displayed in Figure 1 and Table 1. All basil plants show moderate concentrations of dissolved selenium with 0.15 to 0.3 µg/L in the root and a low concentration between 0.05 and 0.1 µg/L in the shoot samples. The root tissues show fairly symmetrical histograms of nano particles with a narrow size distribution between 35 and 80 nm and a maximum between 50 and 55 nm. In accordance with the dissolved selenium, the number of nano particles is low to mediocre compared to other plants. The shoot samples appear to only have a very narrow size distribution with a surprisingly high number of particles in the range between 30 and 50 nm.

Dill shows an astonishing amount of similarity between the root and shoot tissues. SeNPs are found in every plant. The dissolved selenium ranges from 0.3 to 0.5 µg/L in the roots and from 0.3 to 0.8 µg/L in the shoot samples. The maximum for both parts of the plant is between 40 and 60 nm. The histograms show a steep decline for the particles smaller than the maximum and a very flat and slow decline for the particles bigger than the maximum with few particles with a diameter between 300 and 500 nm found in all samples.

**Figure 2:**
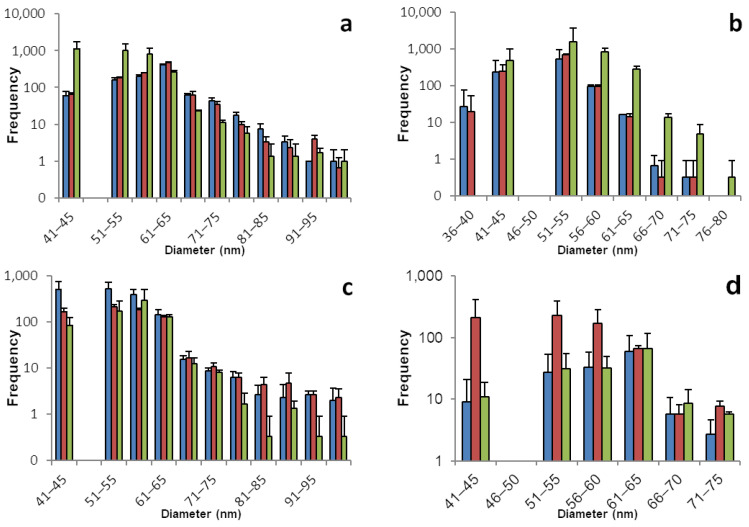
Size distribution of SeNPs in root and shoot tissues of chard and spinach plants. These histograms show the sp-ICP-MS analysis of (**a**) chard root, (**b**) chard shoot, (**c**) spinach root, and (**d**) spinach shoot. Three specimens of each plant were grown, and each bar color represents a single plant. The standard deviation depicted describes the variation for 3 replicates of the same sample. Nano particles with a size of up to 242 nm were detected, however the histograms in this figure are meant to show the main distribution of SeNPs and were therefore cropped. Supplementary information S1 shows the full results. Supplementary information S2 shows histograms including all detected data points for the individual replicates.

The sp-ICP-MS analyses for chard and spinach plants are displayed in Figure 2 and Table 2. Selenium nano particles were found in all chard plants. The samples prepared from plants 1 and 2 had high concentrations above 1 ppm of dissolved selenium, which are connected to an increased potential for false positive results. Those samples were remeasured at half concentration. All root samples show a great number of SeNPs in a broad range with the main share being between 40 and 80 nm in diameter. Fewer particles were found in the shoot tissue, especially for plants 1 and 2, which goes along with a lower concentration of dissolved selenium. Interestingly, the ratio of dissolved to nano selenium in root and shoot tissues for plants 1 and 2 is about 5:1 while for plant 3 the ratio is roughly 3:2. This comes along with a higher number of SeNPs in the shoot of plant 3, hinting that the ratio between dissolved and particulate selenium is not dependent on the organ, but rather the number of SeNPs in a plant part is dependent on the overall selenium concentration.

All spinach plants formed similar amounts of selenium nano particles. With a slightly narrower distribution than chard, SeNPs can be found mostly in the range between 40 and 70 nm. While in plant 1 there is a ratio of roughly 3:2 of dissolved selenium in root compared to shoot samples, the ratio in plants 2 and 3 is close to 1:1. However, here, we can see a larger number of nano particles in the root tissues compared to the respective shoot samples. Keeping in mind that the used method is prone for variation and the exact quantification should not be overestimated it is noticeable here, that unlike in chard plants, there is a tissue-dependent gradient and root samples have a larger amount of their selenium stored as SeNPs than their shoot counterparts. 

**Figure 3:**
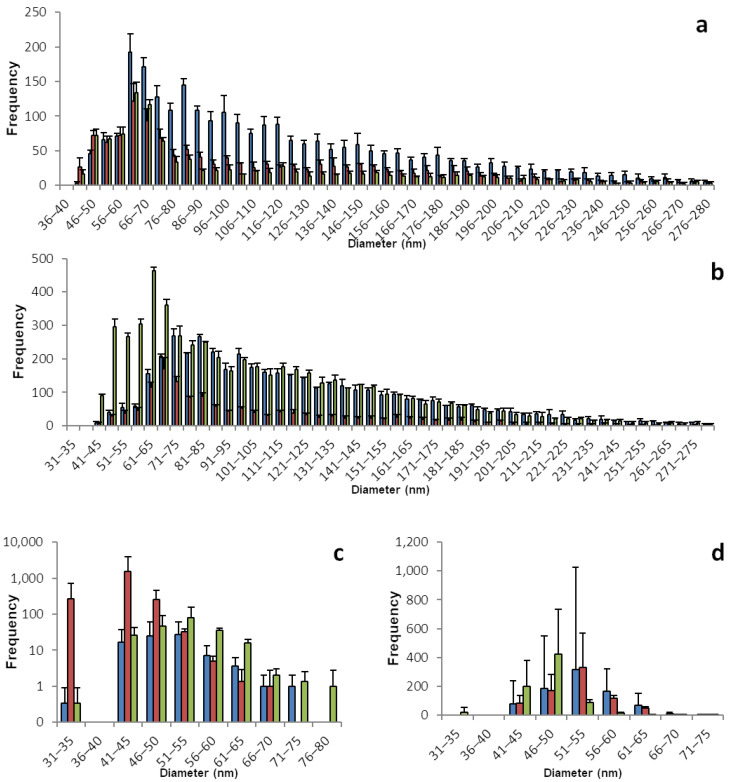
Size distribution of SeNPs in root and shoot tissues of brussels sprout and broccoli plants. These histograms show the sp-ICP-MS analysis of (**a**) brussels sprout root, (**c**) brussels sprout shoot, (**b**) broccoli root, and (**d**) broccoli shoot. Three specimens of each plant were grown, and each bar color represents a single plant. The standard deviation depicted describes the variation for 3 replicates of the same sample. Nano particles with a size of up to 536 nm were detected, however the histograms in this figure are meant to show the main distribution of SeNPs and were therefore cropped. Supplementary information S1 shows the full results. Supplementary information S2 shows histograms including all detected data points for the individual replicates.

The sp-ICP-MS analyses for brussels sprouts and broccoli are displayed in Figure 3 and Table 3. Selenium. Brussels sprouts, especially the root samples show a great number of SeNPs. With a maximum between 50 and 65 nm, all root samples contained nano particles, which were up to 400 nm and over in diameter. In contrast to other plants with few particles larger than 100 nm in diameter, in brussels sprouts roots there was a dense and broad distribution of larger particles. The shoots, on the other hand, had a rather narrow distribution of nano particles, and overall fewer nano particles, mostly in the range between 40 and 60 nm in diameter. Analogous to the particles, the concentration of dissolved selenium was between 0.5 and 0.9 µg/L in the root samples and only between 0.1 and 0.3 µg/L in the shoot samples.

Broccoli, just like brussels sprouts, belongs to the plant family Brassicaceae, which contains all the cruciferous vegetables and that is known to contain many selenium accumulators. Unsurprisingly, the broccoli samples show selenium patterns that are similar to brussels sprouts. A great number of particles with a maximum between 60 and 75 nm in a broad distribution ranging up 500 nm is found in the root samples, which have a concentration of dissolved selenium between 0.4 and 0.6 µg/L.

Equally, the shoot samples of broccoli plants are similar to brussels sprouts with a narrow distribution of nano particles and a maximum between 50 and 60 nm. However, there is less of a gradient in the overall number of nano particles or dissolved selenium in broccoli, compared to brussels sprouts. The shoot samples contain 0.2 to 0.4 µg/L dissolved selenium and a higher number of particles.

**Figure 4:**
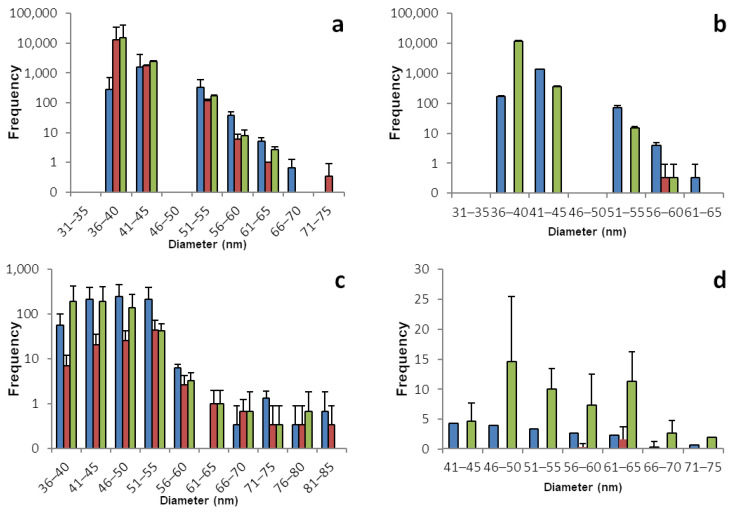
Size distribution of SeNPs in root and shoot tissues of lamb’s lettuce and in Brazil nuts: These histograms show the sp-ICP-MS analysis of (**a**) lamb’s lettuce root, (**b**) lamb’s lettuce shoot, (**c**) the first batch of Brazil nuts, and (**d**) the second batch of Brazil nuts. Three specimens of each plant were grown, and each bar color represents a single plant. The standard deviation depicted describes the variation for 3 replicates of the same sample. Nano particles with a size of up to 273 nm were detected, however the histograms in this figure are meant to show the main distribution of SeNPs and were therefore cropped. Supplementary information S1 shows the full results. Supplementary information S2 shows histograms including all detected data points for the individual replicates.

The sp-ICP-MS analyses for lamb’s lettuce and brazil nuts are displayed in Figure 4 and Table 4. Most of the lamb’s lettuce samples do not give conclusive results. While all of the histograms show SeNPs, most of them do not show a trustworthy distribution. Only a few bars being present in the histograms, often with very high numbers and very low concentrations of dissolved selenium, is a strong hint for false positive results. The case is less clear for lamb’s lettuce roots. Those samples show a more trustworthy distribution of SeNPs ranging from 36 to 70 nm. It can be assumed that the overall low concentration of selenium in lamb’s lettuce is strongly correlated to the lack of SeNPs and especially the shoot samples show close to no reliable hint for the presence of SeNPs, leading to the assumption that unlike the other plants, the consumption of lamb’s lettuce does not include the ingestion of selenium nano particles, as most of lamb’s lettuce’s SeNPs are stored in the roots.

Brazil nuts, being the most prominent representative source of plant-based selenium, have been widely discussed lately for the huge variation of selenium content, which is most probably linked to the strongly varying selenium content of the soil, which Brazil nut trees grow on. We did not grow any plants of this species ourselves, but instead bought two different brands of Brazil nuts from two different local grocery stores. The nuts were treated in the same manner as the plants and the analysis was performed accordingly [41].

And just as the current state of scientific knowledge suggests, the selenium content varies hugely. This is true for the two brands being different from each other, but even the nuts from the same package differ strongly from each other. This also applies to the different runs of sp-ICP-MS analysis that were performed with the exact same sample. When comparing the two batches, referring to the two different bags of nuts, please pay attention to the different dilutions. Replicate 1 was diluted by a factor of 100, compared to replicate number 2.

A reason for the strong differences between the three runs of a sample might be due to aggregation. While we were not yet able to investigate the coating of naturally occurring SeNPs in plants, it is safe to assume that there is a coating around the selenium core. With the exceptionally high amounts of selenium that can be accumulated in Brazil nuts, there might be a unique coating for SeNPs that allows for more efficient detoxification and selenium storage. An aggregation due to the coating might be a reason for a lack of homogenous distribution of SeNPs within the samples.

It should be kept in mind that the analytical method was optimized to reduce dissolved selenium and by doing so, minimizing the potential of false positive results. For Brazil nuts, with naturally high concentrations of selenium, a stronger dilution was needed to avoid false positives. At the expense of a more representative size histogram, we achieved valid and trustworthy results, thus being able to prove that Brazil nuts also contain selenium nano particles. Additionally, it must be kept in mind that nuts are a more complex matrix to work with than shoot and root tissues. The different composition of compounds, especially the lower amount of water in the matrix and the high amounts of proteins and fats, may complicate the extraction of nano particles. Since little is known about naturally occurring SeNPs, it is hard to make an assumption about their resistance to factors like heat or acidity and therefore care has to be taken during the extraction process.

It is, on the other hand, safe to say that Brazil nuts with a high overall concentration of selenium produce a larger number of SeNPs, mainly in the range between 40 and 60 nm. The size distribution of SeNPs in Brazil nuts is rather sharp, with more particles larger than the most frequent size. The histograms overall show a similarity in shape to the ones obtained from shoot and root samples. Despite the dialysis of dissolved selenium, some of the Brazil nuts contain huge amounts of selenium. With a single Brazil nut, the daily recommended dose of selenium could be exceeded by far. On the contrary, some of the Brazil nuts do contain comparably low amounts of selenium and a handful of them might be needed to meet the daily dose of selenium.

## 4. Discussion

SeNPs were found in every observed plant. The nano particles are present in root and shoot tissues and in the case of Brazil nuts, in the nuts themselves as well. The data acquisition time was 120 s and the flow rate was 0.25 mL/min, therefore the SeNPs in 50 µg of plant mass are shown in every histogram, with the exceptions being the chard root samples 1 and 2 representing 25 µg, the first batch of Brazil nuts representing 5 µg, and the second batch of Brazil nuts representing 500 µg as described in the plant treatment section. To ensure that the particles we detected were not root exudates or have a microbiological origin, samples were taken from the growth solution and analyzed in the same way as the plant tissues. No SeNPs were detected by the sp-ICP-MS in those samples. There are differences in the size and size distribution of the particles in the different plant species as well as in the number and proportion of SeNPs. Some observations, however, can be generalized to some extent. Typically, a higher concentration of dissolved selenium is associated with a higher number of SeNPs. The maximum number of SeNPs was found between 40 and 70 nm in all species and the main size ranges are usually not larger than 50 nm. This narrow size distribution is, on the one hand, highly advantageous for possible applications as food supplement or medicinal products and can, on the other hand, be interpreted as a sign for an active metabolic pathway that leads to the nano particle synthesis. In our previous research, we already grew eight different species of plants under the same conditions to evaluate the botanical commonness of SeNP biosynthesis in plants [26]. With these additional plants, we see strong evidence that the naturally occurring synthesis of SeNPs is in fact a ubiquitous phenomenon and we firmly predict that SeNPs can be found in any plant and hence every food plant. It is therefore safe to assume that SeNPs are ubiquitously present in every human’s diet on a daily basis.

The impact of a suboptimal selenium status on human health is not yet fully understood and requires further research. While clear selenium deficiency is well known and described, as well as selenosis from chronic selenium intoxication, there are still many unknown factors for the ideal selenium supply. A high but not oversaturated selenium is linked to reduced mortality by multiple causes including systemic inflammatory response syndrome, sepsis, and cancer mortality including reduced all-cause mortality in prospective studies [12]. Aside from mammals and plants, SeNPs can also have beneficial effects on fish. SeNPs can improve the growth and final weight as well as the antioxidant and immune function in fish. Enriching plant-based fish feed with selenium can therefore supply fish with beneficial SeNPs as well.

A size effect for nano particles was found to be significant for absorption in the gut. For all observed species, the highest count of detected particles was well under 100 nm, mostly between 40 and 70 nm, which is an ideal size for bioavailability in humans. Therefore, we see great nutritious potential for these particles. This particle size is also associated with great cell-barrier penetration. The large surface of SeNPs can, on the other hand, be a disadvantage as well, due to their high surface energy and the potential of precipitation, however compounds such as polyphenols, polysaccharides, and proteins are known to be stabilizing agents. The abundance of such compounds in plants suggests a higher stability of plant-based SeNPs compared to SeNPs that are derived by chemical synthesis.

These findings raise two main questions: What does this mean for agriculture and nutrition? What research is needed in the future? As for nutrition, we know that SeNPs are a great source of selenium, due to their high bioavailability and effect and their low toxicity. These properties make them a highly desirable compound in food. Throughout the last decade, a trend was observed showing that an increasing number of people choose to eat fully plant-based diets with all animal-derived foods excluded. Many experts believe that climate change will force everyone to focus their diet more on plants and massively reduce animal-derived foods. Since selenium is a potentially critical nutrient in the vegan diet, measures need to be taken to ensure a sufficient supply of selenium for the population.

SeNPs are highly desirable candidates for future food supplements. They show great bioavailability and low toxicity, but beyond that, they have unique beneficial effects on health that cannot be observed for other forms of selenium. While a selenite supplement affected pancreatic function and increased adipogenesis and general anabolism in adolescent rats, SeNP supplementation significantly reduced white adipose tissue and BMI [13,42].

The great variations in selenium content of the observed Brazil nuts show even more how complicated selenium nutrition can be. The selenium content of the soil in which Brazil nuts and other plants are grown can be safely assumed to be one of the most critical factors for uptake in plants. Even among trace elements, the range of recommended intake for selenium is particularly narrow. For such a nutrient, it is very important to be aware of the compounds in which it can be found in food [43,44]. In many regions, for example, Nordic countries, the selenium concentration in soil is very low. To achieve sufficient selenium supply, different measures are taken. Unlike the common approach of enriching animal fodder with selenium, Finland took the measure of adding selenium to fertilizers nationwide and thus increased the average daily intake of selenium from 25 µg/day/10 MJ in the 1970s to 80 µg/day/10 MJ today [7]. This is a great example of the benefits of plant-based selenium, and we firmly believe that SeNPs play a huge role in this matter. In many more applications and for a variety of plants, selenium biofortification has proven to be a great way to improve crop selenium content and the nutritious value of plants. The biofortification of crops has been shown to yield promising results through foliar application as well. Therefore, we see potential for future research investigating the binding forms of selenium in crops that have been treated in that manner, as it is more resource-efficient and can help answer the question of whether all plant cells are able to synthesize SeNPs [37,39,45,46].

Selenium is acting as a biostimulant. It can increase the accumulation of bioactive compounds such as vitamin c or flavonoids, which further aid the antioxidant properties of selenium [38,47]. Beneficial effects of selenium on agricultural plants and SeNPs on human health can therefore be achieved simultaneously.

## 5. Conclusions

For the future, we see cause for further research to evaluate the dose-dependent nano particle synthesis in plants and quantitative studies to judge the proportion of selenium that is present in nano form. Selenium deficiency is estimated to affect up to 1 billion people worldwide [48]. Our research shows that it is a highly desirable approach to address this problem by biofortification of crops with selenium. The ubiquitous biosynthesis of SeNPs in food plants results in a high-quality selenium source with great safety and sustainability.

Furthermore, we see cause for research on the coating of these nano particles, as the surface may impact its biological function massively. For this purpose, single-particle-ICP-MS and organic triple-quadrupole mass spectrometry must be combined in the future to determine the organic ligands on the surface of selenium nanoparticles. A complete characterization also requires the determination of the selenium modification present in the core of the nanoparticles. For example, the question arises whether the selenium is present in the red (Se rings) or grey (Se chains) modification, which would cause very different reaction behaviors. To fully assess the potential of plant-based SeNPs as food supplements and components in both natural foods and those enhanced by biofortification, an enhanced method has to be developed that differentiates between organic, inorganic, and nano selenium and can quantify the percentage of selenium that is in nano form. These additional questions very clearly show the challenges that still need to be overcome in the future for an accurate characterization of selenium nanoparticles.

## Figures and Tables

**Table 1:** Concentrations of dissolved selenium corresponding to the histograms shown in Figure 1.

	Root			Shoot		
Basil	1	2	3	1	2	3
Mean concentration of dissolved Se [µg/L]	0.288	0.258	0.166	0.073	0.056	0.106
Standard deviation	0.003	0.006	0.005	0.005	0.003	0.001
Mass of Se per kg plant mass (fresh weight) [mg]	2.88	2.58	1.66	0.73	0.56	1.06
Dill						
Mean concentration of dissolved Se [µg/L]	0.495	0.433	0.339	0.374	0.817	0.579
Standard deviation	0.029	0.011	0.007	0.003	0.019	0.007
Mass of Se per kg plant mass (fresh weight) [mg]	4.95	4.33	3.39	3.74	8.17	5.79

**Table 2:** Concentrations of dissolved selenium corresponding to the histograms shown in Figure 2.

	Root			Shoot		
Chard	1	2	3	1	2	3
Mean concentration of dissolved Se [µg/L]	0.404	0.453	0.742	0.194	0.176	0.462
Standard deviation	0.012	0.009	0.023	0.009	0.004	0.006
Mass of Se per kg plant mass (fresh weight) [mg]	4.04	4.53	7.42	1.94	1.76	4.62
Spinach						
Mean concentration of dissolved Se [µg/L]	0.565	0.560	0.362	0.357	0.552	0.378
Standard deviation	0.032	0.021	0.010	0.007	0.011	0.009
Mass of Se per kg plant mass (fresh weight) [mg]	5.65	5.60	3.62	3.57	5.52	3.78

**Table 3:** Concentrations of dissolved selenium corresponding to the histograms shown in Figure 3.

	Root			Shoot		
Brussels Sprout	1	2	3	1	2	3
Mean concentration of dissolved Se [µg/L]	0.941	0.522	0.698	0.205	0.133	0.284
Standard deviation	0.007	0.009	0.002	0.002	0.001	0.001
Mass of Se per kg plant mass (fresh weight) [mg]	9.41	5.22	6.98	2.05	1.33	2.84
Broccoli						
Mean concentration of dissolved Se [µg/L]	0.461	0.420	0.601	0.400	0.373	0.192
Standard deviation	0.017	0.001	0.001	0.007	0.034	0.002
Mass of Se per kg plant mass (fresh weight) [mg]	4.61	4.20	6.01	4.00	3.73	1.92

**Table 4:** Concentrations of dissolved selenium corresponding to the histograms shown in Figure 4.

	Root			Shoot		
Lamb’s Lettuce	1	2	3	1	2	3
Mean concentration of dissolved Se [µg/L]	0.204	0.084	0.113	0.062	0.040	0.002
Standard deviation	0.023	0.006	0.004	0.003	0.003	0.001
Mass of Se per kg plant mass (fresh weight) [mg]	2.04	0.84	1.13	0.62	0.40	0.02
Brazil Nut	Batch 1			Batch 2		
Mean concentration of dissolved Se [µg/L]	0.419	0.173	0.309	0.647	0.476	0.854
Standard deviation	0.229	0.008	0.003	0.011	0.009	0.018
Mass of Se per kg plant mass (fresh weight) [mg]	41.9	17.3	30.9	0.647	0.476	0.854
Mass of Se per Brazil nut (assuming an average weight of 5 g/nut) [µg]	209.5	86.5	154.5	3.234	2.36	4267

## Data Availability

The data for Figure 1, Figure 2, Figure 3 and Figure 4 are shown in the supplementary information.

## Supplementary Materials

The following supporting information can be downloaded at: https://www.mdpi.com/article/10.3390/foods12173203/s1, Supplementary Information S1: Raw data of the *sp-ICP-MS* results included in this study; Supplementary Information S2: all individual data points for the histograms included in Figure 1, Figure 2, Figure 3 and Figure 4.

## References

[1] EFSA Panel on Dietetic Products, Nutrition and Allergies (NDA) (2014). Scientific Opinion on Dietary Reference Values for selenium. EFSA J..

[2] Melse-Boonstra A. (2020). Bioavailability of Micronutrients From Nutrient-Dense Whole Foods: Zooming in on Dairy, Vegetables, and Fruits. Front. Nutr..

[3] Hurrell R., Egli I. (2010). Iron bioavailability and dietary reference values. Am. J. Clin. Nutr..

[4] Hadrup N., Ravn-Haren G. (2021). Absorption, distribution, metabolism and excretion (ADME) of oral selenium from organic and inorganic sources: A review. J. Trace Elem. Med. Biol..

[5] Gu X., Gao C.-Q. (2022). New horizons for selenium in animal nutrition and functional foods. Anim. Nutr..

[6] White P.J. (2016). Selenium accumulation by plants. Ann. Bot..

[7] Alfthan G., Eurola M., Ekholm P., Venäläinen E.-R., Root T., Korkalainen K., Hartikainen H., Salminen P., Hietaniemi V., Aspila P. (2015). Effects of nationwide addition of selenium to fertilizers on foods, and animal and human health in Finland: From deficiency to optimal selenium status of the population. J. Trace Elem. Med. Biol..

[8] White P.J. (2018). Selenium metabolism in plants. Biochim. Biophys. Acta Gen. Subj..

[9] Schiavon M., Pilon-Smits E.A.H. (2017). The fascinating facets of plant selenium accumulation—Biochemistry, physiology, evolution and ecology. New Phytol..

[10] Gupta M., Gupta S. (2016). An Overview of Selenium Uptake, Metabolism, and Toxicity in Plants. Front. Plant. Sci..

[11] Reeves M.A., Hoffmann P.R. (2009). The human selenoproteome: Recent insights into functions and regulation. Cell. Mol. Life Sci..

[12] Rayman M.P. (2012). Selenium and human health. Lancet.

[13] Chen J., Guo Y., Zhang X., Liu J., Gong P., Su Z., Fan L., Li G. (2023). Emerging Nanoparticles in Food: Sources, Application, and Safety. J. Agric. Food Chem..

[14] Carlisle A.E., Lee N., Matthew-Onabanjo A.N., Spears M.E., Park S.J., Youkana D., Doshi M.B., Peppers A., Li R., Joseph A.B. (2020). Selenium detoxification is required for cancer-cell survival. Nat. Metab..

[15] Kang D., Lee J., Wu C., Guo X., Lee B.J., Chun J.-S., Kim J.-H. (2020). The role of selenium metabolism and selenoproteins in cartilage homeostasis and arthropathies. Exp. Mol. Med..

[16] Pyrzynska K., Sentkowska A. (2022). Biosynthesis of selenium nanoparticles using plant extracts. J. Nanostruct. Chem..

[17] Shimada B.K., Alfulaij N., Seale L.A. (2021). The Impact of Selenium Deficiency on Cardiovascular Function. Int. J. Mol. Sci..

[18] Hughes D.J., Duarte-Salles T., Hybsier S., Trichopoulou A., Stepien M., Aleksandrova K., Overvad K., Tjønneland A., Olsen A., Affret A. (2016). Prediagnostic selenium status and hepatobiliary cancer risk in the European Prospective Investigation into Cancer and Nutrition cohort. Am. J. Clin. Nutr..

[19] Lin Z., Li Y., Guo M., Xiao M., Wang C., Zhao M., Xu T., Xia Y., Zhu B. (2017). Inhibition of H1N1 influenza virus by selenium nanoparticles loaded with zanamivir through p38 and JNK signaling pathways. RSC Adv..

[20] He L., Zhao J., Wang L., Liu Q., Fan Y., Li B., Yu Y.-L., Chen C., Li Y.-F. (2021). Using nano-selenium to combat Coronavirus Disease 2019 (COVID-19)?. Nano Today.

[21] Hoffman K.S., Vargas-Rodriguez O., Bak D.W., Mukai T., Woodward L.K., Weerapana E., Söll D., Reynolds N.M. (2019). A cysteinyl-tRNA synthetase variant confers resistance against selenite toxicity and decreases selenocysteine misincorporation. J. Biol. Chem..

[22] Jia X., Li N., Chen J. (2005). A subchronic toxicity study of elemental Nano-Se in Sprague-Dawley rats. Life Sci..

[23] Wang H., Zhang J., Yu H. (2007). Elemental selenium at nano size possesses lower toxicity without compromising the fundamental effect on selenoenzymes: Comparison with selenomethionine in mice. Free Radic. Biol. Med..

[24] Menon S., Ks S.D., Santhiya R., Rajeshkumar S., Kumar V. (2018). Selenium nanoparticles: A potent chemotherapeutic agent and an elucidation of its mechanism. Colloids Surf. B Biointerfaces.

[25] Bhattacharjee A., Basu A., Bhattacharya S. (2019). Selenium nanoparticles are less toxic than inorganic and organic selenium to mice in vivo. Nucleus.

[26] Verstegen J., Günther K. (2023). Biosynthesis of nano selenium in plants. Artif. Cells Nanomed. Biotechnol..

[27] Rai R.K., Karri R., Dubey K.D., Roy G. (2022). Regulation of Tyrosinase Enzyme Activity by Glutathione Peroxidase Mimics. J. Agric. Food Chem..

[28] Kristal A.R., Darke A.K., Morris J.S., Tangen C.M., Goodman P.J., Thompson I.M., Meyskens F.L., Goodman G.E., Minasian L.M., Parnes H.L. (2014). Baseline selenium status and effects of selenium and vitamin e supplementation on prostate cancer risk. J. Natl. Cancer Inst..

[29] Rayman M.P. (2020). Selenium intake, status, and health: A complex relationship. Hormones.

[30] Sun X.-H., Lv M.-W., Zhao Y.-X., Zhang H., Ullah Saleem M.A., Zhao Y., Li J.-L. (2023). Nano-Selenium Antagonized Cadmium-Induced Liver Fibrosis in Chicken. J. Agric. Food Chem..

[31] Nayak V., Singh K.R.B., Singh A.K., Singh R.P. (2021). Potentialities of selenium nanoparticles in biomedical science. New J. Chem..

[32] Liao G., Tang J., Wang D., Zuo H., Zhang Q., Liu Y., Xiong H. (2020). Selenium nanoparticles (SeNPs) have potent antitumor activity against prostate cancer cells through the upregulation of miR-16. World J. Surg. Oncol..

[33] Abd-Rabou A.A., Ahmed H.H., Shalby A.B. (2020). Selenium Overcomes Doxorubicin Resistance in Their Nano-platforms Against Breast and Colon Cancers. Biol. Trace Elem. Res..

[34] Peng D., Zhang J., Liu Q., Taylor E.W. (2007). Size effect of elemental selenium nanoparticles (Nano-Se) at supranutritional levels on selenium accumulation and glutathione S-transferase activity. J. Inorg. Biochem..

[35] Dolai J., Mandal K., Jana N.R. (2021). Nanoparticle Size Effects in Biomedical Applications. ACS Appl. Nano Mater..

[36] Kolbert Z., Molnár Á., Feigl G., van Hoewyk D. (2019). Plant selenium toxicity: Proteome in the crosshairs. J. Plant Physiol..

[37] Silva M.A., de Sousa G.F., Corguinha A.P.B., de Lima Lessa J.H., Dinali G.S., Oliveira C., Lopes G., Amaral D., Brown P., Guilherme L.R.G. (2022). Selenium biofortification of soybean genotypes in a tropical soil via Se-enriched phosphate fertilizers. Front. Plant Sci..

[38] Hossain M.A., Ahammed G.J., Kolbert Z., El-Ramady H., Islam T., Schiavon M. (2022). Selenium and Nano-Selenium in Environmental Stress Management and Crop Quality Improvement.

[39] Hossain A., Skalicky M., Brestic M., Maitra S., Sarkar S., Ahmad Z., Vemuri H., Garai S., Mondal M., Bhatt R. (2021). Selenium Biofortification: Roles, Mechanisms, Responses and Prospects. Molecules.

[40] Cheng C., Coldea T.E., Yang H., Zhao H. (2023). Selenium Uptake, Translocation, and Metabolization Pattern during Barley Malting: A Comparison of Selenate, Selenite, and Selenomethionine. J. Agric. Food Chem..

[41] Silva Junior E.C., Wadt L.H.O., Silva K.E., Lima R.M.B., Batista K.D., Guedes M.C., Carvalho G.S., Carvalho T.S., Reis A.R., Lopes G. (2017). Natural variation of selenium in Brazil nuts and soils from the Amazon region. Chemosphere.

[42] Ojeda M.L., Nogales F., Carreras O., Pajuelo E., Del Gallego-López M.C., Romero-Herrera I., Begines B., Moreno-Fernández J., Díaz-Castro J., Alcudia A. (2022). Different Effects of Low Selenite and Selenium-Nanoparticle Supplementation on Adipose Tissue Function and Insulin Secretion in Adolescent Male Rats. Nutrients.

[43] Bakaloudi D.R., Halloran A., Rippin H.L., Oikonomidou A.C., Dardavesis T.I., Williams J., Wickramasinghe K., Breda J., Chourdakis M. (2021). Intake and adequacy of the vegan diet. A systematic review of the evidence. Clin. Nutr..

[44] Weder S., Zerback E.H., Wagener S.M., Koeder C., Fischer M., Alexy U., Keller M. (2022). How Does Selenium Intake Differ among Children (1–3 Years) on Vegetarian, Vegan, and Omnivorous Diets? Results of the VeChi Diet Study. Nutrients.

[45] Zou C., Du Y., Rashid A., Ram H., Savasli E., Pieterse P.J., Ortiz-Monasterio I., Yazici A., Kaur C., Mahmood K. (2019). Simultaneous Biofortification of Wheat with Zinc, Iodine, Selenium, and Iron through Foliar Treatment of a Micronutrient Cocktail in Six Countries. J. Agric. Food Chem..

[46] Duborská E., Šebesta M., Matulová M., Zvěřina O., Urík M. (2022). Current Strategies for Selenium and Iodine Biofortification in Crop Plants. Nutrients.

[47] Dima S.-O., Neamțu C., Desliu-Avram M., Ghiurea M., Capra L., Radu E., Stoica R., Faraon V.-A., Zamfiropol-Cristea V., Constantinescu-Aruxandei D. (2020). Plant Biostimulant Effects of Baker’s Yeast Vinasse and Selenium on Tomatoes through Foliar Fertilization. Agronomy.

[48] Shreenath A.P., Ameer M.A., Dooley J. (2023). StatPearls: Selenium Deficiency. Treasure Island.

