# Supplementary material for: Ubiquitous Occurrence of Nano Selenium in Food Plants
Source: Foods. 2023 Aug 25;12(17):3203. doi: 10.3390/foods12173203 (PMC10487048; doi:10.3390/foods12173203)
Supplement: Supplementary file 1 [file foods-12-03203-s001.zip › Supplementary information S1 sp-ICP-MS data tables.pdf]

Supplementary information S1 for

Ubiquitous occurrence of nano selenium in food plants

Jonas Verstegen, Klaus Günther*
*Corresponding author. Email: k.guenther@fz-juelich.de

The following table includes the raw data for every sp-ICP-MS analysis that was included in this study. While the figures in the paper focus on the essential share of particles the following table gives a full list of particles.

Tabelle 1: Raw data of the sp-ICP-MS results included in this study

	Basil	Root	Plant 1
	Run 1	Run 2	Run 3
Diameter (nm)	Frequency	Frequency	Frequency
30	0	0	0
31	0	0	0
32	0	0	0
33	0	0	0
34	0	0	0
35	0	0	0
36	0	0	0
37	0	0	0
38	29	10	28
39	0	0	0
40	0	0	0
41	0	0	0
42	0	0	0
43	0	0	0
44	83	27	103
45	0	0	0
46	0	0	0
47	0	0	0
48	115	45	95
49	0	0	0
50	0	0	0
51	0	0	0
52	160	40	130
53	0	0	0
54	0	0	0
55	64	61	64
56	0	0	0
57	0	0	0
58	28	28	29

59	0	0	0
60	0	0	0
61	17	12	17
62	0	0	0
63	13	8	8
64	0	0	0
65	3	5	1
66	0	0	0
67	0	0	0
68	6	5	4
69	0	0	0
70	3	2	3
71	1	2	5
72	0	0	0
73	1	1	3
74	0	0	0
75	1	1	0
76	0	0	0
77	0	2	3
78	0	1	0
79	0	0	0
80	1	2	1
81	2	1	0
82	0	0	0
83	0	0	0
84	1	0	0
85	1	0	1
86	0	0	0
87	0	0	0
88	0	1	0
89	0	0	0
90	0	0	0
91	0	0	0
92	0	0	1
93	1	0	1
94	0	0	0
95	0	0	0
96	0	1	0
97	0	0	0
98	0	1	0
99	0	1	0
100	1		0
101			0
102			0
103			0
104			0
105			0

106			0
107			0
108			1

	Basil	Root	Plant 2
	Run 1	Run 2	Run 3
Diameter (nm)	Frequency	Frequency	Frequency
30	0	0	0
31	0	0	0
32	0	0	0
33	0	0	0
34	0	0	0
35	0	0	0
36	0	0	0
37	0	0	0
38	3	2	2
39	0	0	0
40	0	0	0
41	0	0	0
42	0	0	0
43	0	0	0
44	8	17	15
45	0	0	0
46	0	0	0
47	0	0	0
48	25	15	19
49	0	0	0
50	0	0	0
51	0	0	0
52	19	10	9
53	0	0	0
54	0	0	0
55	21	30	16
56	0	0	0
57	0	0	0
58	15	14	12
59	0	0	0
60	0	0	0
61	8	7	12
62	0	0	0
63	5	12	3
64	0	0	0
65	6	5	2
66	0	0	0
67	0	0	0

68	6	6	3
69	0	0	0
70	4	5	3
71	1	2	1
72	0	0	0
73	2	2	2
74	0	0	0
75	0	1	2
76	0	0	0
77	1	2	0
78	3	1	2
79	0	0	0
80	1	2	0
81	0	0	1
82	1	0	0
83	0	0	0
84	1	0	0
85	1	0	0
86	0	0	4
87	0	0	0
88	0	0	0
89	1	0	1
90	1	0	1
91	0	0	0
92	0	0	1
93	0	0	1
94	0	1	0
95	0	0	0
96	0	0	1
97	0	0	
98	0	0	
99	1	1	
100	0	0	
101	0	0	
102	0	0	
103	0	0	
104	0	0	
105	1	0	
106	2	0	
107		0	
108		0	
109		1	
110		0	
111		0	
112		0	
113		0	
114		0	

115	0
116	0
117	0
118	1
119	0
120	1
121	0
122	0
123	0
124	0
125	0
126	0
127	0
128	0
129	0
130	0
131	0
132	0
133	0
134	0
135	0
136	0
137	0
138	0
139	0
140	0
141	0
142	0
143	0
144	0
145	0
146	0
147	0
148	0
149	0
150	0
151	1

	Basil	Root	Plant 3
	Run 1	Run 2	Run 3
Diameter (nm)	Frequency	Frequency	Frequency
30	0	1	0
31	0	0	0
32	0	0	0
33	0	0	0

34	0	0	0
35	0	0	0
36	0	0	0
37	0	0	0
38	3	16	12
39	0	0	0
40	0	0	0
41	0	0	0
42	0	0	0
43	0	0	0
44	4	19	12
45	0	0	0
46	0	0	0
47	0	0	0
48	6	21	24
49	0	0	0
50	0	0	0
51	0	0	0
52	9	34	31
53	0	0	0
54	0	0	0
55	6	4	4
56	0	0	0
57	0	0	0
58	6	5	4
59	0	0	0
60	0	0	0
61	7	3	3
62	0	0	0
63	3	6	2
64	0	0	0
65	3	2	1
66	0	0	0
67	0	0	0
68	0	1	4
69	0	0	0
70	2	1	1
71	2	2	1
72	0	0	0
73	4	1	0
74	0	0	0
75	0	3	1
76	0	0	0
77	2	1	2
78	0	3	0
79	0	0	0
80	1	0	0

81	2	1	1
82	0	1	0
83	0	0	0
84	1	0	2
85	0	0	0
86	0	0	0
87	0	0	0
88	0	0	1
89	1	0	0
90	1	0	0
91	1	0	1
92	0	0	0
93	0	0	0
94	0	1	1
95	1	0	0
96	0	0	1
97	1	0	0
98	0	0	0
99	0	0	0
100	0	0	0
101	0	0	0
102	1	0	0
103	0	0	1
104	0	0	0
105	0	0	0
106	0	0	0
107	0	0	0
108	0	0	0
109	0	1	0
110	0	0	0
111	0	0	1
112	0	0	
113	0	1	
114	0	0	
115	0	0	
116	0	0	
117	0	0	
118	0	0	
119	0	0	
120	0	0	
121	1	0	
122		0	
123		0	
124		0	
125		0	
126		0	
127		0	

128	0
129	0
130	0
131	0
132	0
133	0
134	0
135	0
136	0
137	0
138	0
139	0
140	0
141	0
142	0
143	0
144	0
145	0
146	0
147	0
148	0
149	0
150	0
151	0
152	0
153	0
154	0
155	0
156	0
157	0
158	0
159	0
160	0
161	0
162	0
163	0
164	1

	Basil	Shoot	Plant 1
	Run 1	Run 2	Run 3
Diameter (nm)	Frequency	Frequency	Frequency
30	722	19	9
31	0	0	0
32	0	0	0
33	0	0	0

34	0	0	0
35	0	0	0
36	0	0	0
37	0	0	0
38	4167	113	123
39	0	0	0
40	0	0	0
41	0	0	0
42	0	0	0
43	0	0	0
44	348	230	215
45	0	0	0
46	0	0	0
47	0	0	0
48	24	25	17
49	0	0	0
50	0	0	0
51	0	0	0
52	4	0	5
53	0	0	0
54	0	0	0
55	0	2	0
56	0	0	0
57	0	0	0
58	0	0	0
59	0	0	0
60	0	0	0
61	0	0	0
62	0	0	0
63	0	0	0
64	0	0	0
65	0	1	0
66	0	0	0
67	0	0	0
68	0	0	0
69	0	0	0
70	0	0	0
71	1	0	0
72		0	0
73		0	0
74		0	0
75		0	0
76		0	0
77		0	0
78		0	0
79		0	0
80		0	0

81	0	0
82	0	0
83	0	0
84	0	0
85	0	0
86	0	0
87	0	0
88	0	0
89	0	0
90	0	0
91	0	0
92	0	0
93	0	0
94	0	0
95	0	0
96	0	0
97	0	0
98	0	0
99	0	0
100	0	0
101	0	0
102	0	0
103	0	0
104	0	0
105	0	0
106	0	0
107	0	0
108	0	0
109	0	0
110	0	0
111	0	0
112	0	0
113	0	0
114	0	0
115	0	0
116	0	0
117	0	0
118	1	0
119		0
120		0
121		0
122		0
123		0
124		0
125		0
126		0
127		0

128	0
129	0
130	0
131	0
132	0
133	0
134	0
135	0
136	0
137	0
138	0
139	0
140	0
141	0
142	0
143	0
144	0
145	0
146	0
147	0
148	0
149	0
150	0
151	0
152	0
153	0
154	0
155	0
156	0
157	0
158	1

	Basil	Shoot	Plant 2
	Run 1	Run 2	Run 3
Diameter (nm)	Frequency	Frequency	Frequency
30	0	13	34
31	0	0	0
32	0	0	0
33	0	0	0
34	0	0	0
35	0	0	0
36	0	0	0
37	0	0	0
38	0	96	150
39	0	0	0

40	0	0	0
41	0	0	0
42	0	0	0
43	0	0	0
44	0	211	198
45	0	0	0
46	0	0	0
47	0	0	0
48	0	15	23
49	0	0	0
50	0	0	0
51	0	0	0
52	0	2	3
53	0	0	0
54	0	0	0
55	0	1	2
56	0	0	0
57	0	0	0
58	0	0	0
59	0	0	0
60	0	0	0
61	0	1	0
62	0	0	0
63	1	0	0
64	0	0	0
65	0	0	0
66	0	0	0
67	0	0	0
68	0	0	0
69	0	0	0
70	1	1	0
71	0	1	0
72	0	0	0
73	0	0	0
74	0	0	0
75	0	0	1
76	0	0	0
77	0	0	0
78	0	0	0
79	0	0	0
80	0	0	0
81	0	0	0
82	0	1	0
83	0	0	0
84	0	0	0
85	0	0	0
86	0	0	0

87	0	0	0
88	0	0	0
89	0	0	0
90	0	0	0
91	0	0	0
92	0	0	0
93	1	0	0
94	0	0	0
95	0	0	0
96	0	0	0
97	0	0	0
98	0	0	0
99	0	0	0
100	0	0	0
101	0	0	0
102	0	0	0
103	0	0	0
104	0	0	0
105	1	0	0
106	0	0	0
107	0	0	0
108	0	0	0
109	0	0	0
110	0	1	0
111	0		0
112	0		0
113	0		0
114	0		0
115	0		0
116	0		0
117	0		0
118	0		0
119	0		0
120	0		0
121	0		0
122	0		0
123	0		0
124	0		0
125	0		0
126	0		0
127	0		0
128	0		0
129	0		0
130	0		0
131	0		0
132	0		0
133	0		0

134	0	0
135	0	0
136	0	0
137	0	0
138	0	0
139	0	0
140	0	0
141	0	0
142	0	0
143	0	0
144	0	0
145	0	0
146	0	0
147	0	0
148	0	0
149	0	0
150	0	0
151	0	0
152	0	0
153	0	0
154	0	0
155	0	0
156	0	0
157	0	0
158	0	0
159	0	0
160	0	0
161	0	0
162	0	0
163	0	0
164	0	0
165	0	0
166	0	0
167	0	0
168	0	0
169	0	0
170	0	0
171	0	0
172	0	0
173	0	0
174	0	0
175	0	0
176	0	0
177	0	0
178	0	0
179	0	0
180	0	0

181	0	0
182	0	0
183	0	0
184	0	0
185	0	0
186	0	0
187	0	0
188	0	0
189	0	0
190	0	0
191	0	0
192	0	0
193	0	0
194	0	0
195	0	0
196	0	0
197	0	0
198	0	0
199	0	0
200	0	0
201	0	0
202	0	0
203	0	0
204	0	0
205	0	0
206	0	0
207	0	0
208	0	0
209	0	0
210	0	0
211	0	0
212	0	0
213	0	1
214	0	
215	0	
216	0	
217	0	
218	0	
219	0	
220	0	
221	0	
222	0	
223	0	
224	0	
225	0	
226	0	
227	0	

228	0
229	0
230	0
231	0
232	0
233	0
234	0
235	0
236	0
237	0
238	0
239	0
240	0
241	0
242	0
243	0
244	0
245	0
246	0
247	0
248	0
249	0
250	0
251	0
252	0
253	0
254	0
255	0
256	0
257	0
258	0
259	0
260	0
261	0
262	0
263	0
264	0
265	0
266	0
267	0
268	0
269	0
270	0
271	0
272	0
273	0
274	0

275	0
276	0
277	0
278	0
279	0
280	0
281	0
282	0
283	0
284	0
285	0
286	0
287	0
288	0
289	0
290	0
291	0
292	0
293	0
294	0
295	0
296	0
297	0
298	0
299	0
300	0
301	0
302	0
303	0
304	0
305	0
306	0
307	0
308	0
309	0
310	0
311	0
312	0
313	0
314	0
315	0
316	0
317	0
318	1

Basil Shoot Plant 3

Diameter (nm)	Run 1 Frequency	Run 2 Frequency	Run 3 Frequency
30	57	1141	60
31	0	0	0
32	0	0	0
33	0	0	0
34	0	0	0
35	0	0	0
36	0	0	0
37	0	0	0
38	383	5890	326
39	0	0	0
40	0	0	0
41	0	0	0
42	0	0	0
43	0	0	0
44	530	788	657
45	0	0	0
46	0	0	0
47	0	0	0
48	114	89	104
49	0	0	0
50	0	0	0
51	0	0	0
52	25	23	18
53	0	0	0
54	0	0	0
55	3	1	4
56	0	0	0
57	0	0	0
58	1	0	2
59	0	0	0
60	0	0	0
61	1	0	1
62	0	0	0
63	1	0	0
64		0	0
65		0	0
66		0	0
67		0	0
68		0	1
69		0	0
70		0	0
71		0	0
72		0	0
73		1	0

74			0
75			0
76			0
77			0
78			0
79			0
80			0
81			0
82			0
83			0
84			0
85			0
86			0
87			0
88			0
89			0
90			0
91			0
92			0
93			0
94			0
95			0
96			0
97			0
98			0
99			0
100			0
101			0
102			0
103			0
104			0
105			0
106			0
107			0
108			0
109			0
110			0
111			0
112			1

	Dill	Root	Plant 1
Diameter (nm)	Run 1 Frequency	Run 2 Frequency	Run 3 Frequency
30	0	0	0

31	0	0	0
32	0	0	0
33	0	0	0
34	0	0	0
35	0	0	0
36	0	0	0
37	0	0	0
38	8	2	2
39	0	0	0
40	0	0	0
41	0	0	0
42	0	0	0
43	0	0	0
44	23	11	10
45	0	0	0
46	0	0	0
47	0	0	0
48	17	14	13
49	0	0	0
50	0	0	0
51	0	0	0
52	29	18	11
53	0	0	0
54	0	0	0
55	31	12	4
56	0	0	0
57	0	0	0
58	19	8	9
59	0	0	0
60	0	0	0
61	19	17	7
62	0	0	0
63	20	14	12
64	0	0	0
65	9	8	7
66	0	0	0
67	0	0	0
68	17	4	12
69	0	0	0
70	6	6	5
71	1	4	4
72	0	0	0
73	3	2	5
74	0	0	0
75	2	1	3
76	0	0	0
77	2	6	3

78	4	2	1
79	0	0	0
80	4	4	2
81	1	4	3
82	2	3	1
83	0	0	0
84	3	1	1
85	4	1	1
86	5	1	2
87	0	0	0
88	2	1	2
89	1	0	1
90	0	0	0
91	1	0	0
92	2	2	0
93	1	1	1
94	0	0	2
95	0	0	1
96	2	1	0
97	0	0	3
98	0	0	2
99	3	1	0
100	2	1	0
101	0	0	1
102	0	1	0
103	1	2	2
104	1	0	1
105	4	1	0
106	2	1	2
107	0	0	2
108	0	0	1
109	1	1	0
110	3	1	1
111	1	1	1
112	1	0	0
113	1	2	1
114	0	1	2
115	2	1	1
116	1	0	1
117	3	0	2
118	2	1	1
119	2	1	0
120	1	0	1
121	1	0	0
122	2	1	0
123	0	2	0
124	0	1	0

125	0	2	1
126	2	1	1
127	1	1	1
128	1	0	1
129	0	0	0
130	0	1	1
131	1	0	1
132	0	0	1
133	0	0	0
134	0	1	1
135	0	0	1
136	2	0	0
137	1	1	1
138	1	1	1
139	0	0	2
140	0	0	0
141	1	1	0
142	2	1	0
143	3	1	1
144	0	3	2
145	0	3	3
146	1	0	1
147	0	0	3
148	1	2	0
149	0	0	1
150	2	1	0
151	1	0	1
152	2	1	0
153	1	0	0
154	1	2	0
155	1	0	2
156	1	0	0
157	0	2	0
158	2	1	0
159	1	1	1
160	2	1	0
161	1	2	1
162	1	3	0
163	0	1	0
164	2	1	0
165	0	0	1
166	0	0	0
167	0	1	0
168	2	0	1
169	0	3	1
170	0	0	2
171	0	0	0

172	0	1	2
173	1	0	0
174	1	1	2
175	0	0	0
176	1	0	0
177	2	1	0
178	1	0	0
179	2	0	0
180	1	1	0
181	0	0	1
182	1	0	0
183	0	1	0
184	1	1	0
185	0	1	2
186	1	0	1
187	0	0	1
188	0	0	0
189	0	1	0
190	1	0	0
191	1	0	0
192	1	1	1
193	2	1	0
194	0	0	1
195	0	0	0
196	1	0	0
197	0	0	0
198	0	0	1
199	0	0	1
200	0	2	0
201	0	0	0
202	0	0	1
203	3	1	1
204	0	0	0
205	0	0	0
206	0	1	0
207	0	0	1
208	2	0	0
209	0	0	0
210	0	2	0
211	0	0	1
212	0	0	0
213	1	0	1
214	0	0	0
215	0	0	0
216	0	0	0
217	0	0	1
218	0	1	0

219	0	1	0
220	0	0	1
221	1	1	0
222	0	0	0
223	0	1	2
224	1	0	0
225	1	1	0
226	0	0	0
227	0	1	0
228	1	0	0
229	1	0	0
230	0	0	0
231	0	0	1
232	0	0	0
233	0	0	1
234	0	0	1
235	0	0	0
236	0	1	0
237	0	1	0
238	0	0	0
239	0	0	0
240	1	1	1
241	2	0	1
242	0	1	0
243	2	0	0
244	0	1	0
245	0	0	0
246	0	0	1
247	1	0	0
248	0	0	0
249	1	0	0
250	0	1	1
251	0	0	1
252	0	0	0
253	0	0	1
254	0	0	0
255	0	0	2
256	2	0	0
257	0	0	0
258	0	0	0
259	0	0	0
260	0	0	1
261	0	0	0
262	0	0	0
263	1	0	1
264	0	0	0
265	1	0	0

266	0	0	0
267	2	0	0
268	0	0	0
269	0	0	0
270	0	0	0
271	0	0	0
272	0	0	0
273	0	0	0
274	0	1	1
275	0	0	0
276	0	1	0
277	0	0	0
278	1	0	0
279	0	0	0
280	1	1	1
281	1	0	0
282	0	0	0
283	0	0	0
284	0	0	1
285	0	0	0
286	0	0	0
287	0	0	1
288	0	0	0
289	0	2	0
290	0	1	0
291	0	0	0
292	0	0	0
293	1	0	0
294	0	2	0
295	0	0	1
296	0	0	0
297	0	0	0
298	0	1	0
299	0	0	0
300	0	0	0
301	1	0	0
302	0	0	0
303	0	0	0
304	0	0	1
305	0	0	0
306	0	0	1
307	0	0	0
308	1	0	0
309	0	0	1
310	0	0	0
311	0	1	0
312	1	0	1

313	0	0	0
314	0	0	0
315	0	0	1
316	0	0	0
317	0	0	0
318	0	1	0
319	0	0	0
320	0	0	0
321	0	0	0
322	0	0	0
323	0	0	0
324	0	0	0
325	0	0	0
326	0	0	0
327	0	0	0
328	0	0	0
329	0	0	0
330	1	1	0
331	0	0	0
332	0	0	0
333	0	0	0
334	0	0	0
335	0	0	0
336	1	0	0
337	1	0	0
338	0	0	0
339	0	0	0
340	0	0	0
341	1	0	0
342	0	0	0
343	0	0	0
344	0	0	0
345	0	0	0
346	0	0	0
347	0	0	0
348	0	0	0
349	0	0	0
350	0	0	0
351	0	0	0
352	0	0	0
353	0	0	0
354	0	0	0
355	0	0	0
356	0	0	0
357	0	0	0
358	0	0	0
359	0	0	0

360	0	0	0
361	1	0	0
362	0	0	0
363	0	0	0
364	0	0	0
365	0	0	0
366	0	0	0
367	0	0	0
368	0	2	0
369	0	0	0
370	0	0	0
371	0	0	0
372	0	0	1
373	1	0	0
374	0	1	0
375	0	0	0
376	0	0	0
377	0	0	0
378	0	0	0
379	0	0	0
380	0	0	0
381	0	0	0
382	0	0	0
383	0	0	0
384	0	0	0
385	0	0	0
386	0	0	0
387	0	0	0
388	0	0	0
389	0	0	0
390	0	0	0
391	0	0	0
392	0	0	0
393	0	0	0
394	0	0	0
395	0	0	0
396	0	0	0
397	0	1	0
398	0		0
399	0		0
400	0		0
401	0		0
402	0		0
403	0		0
404	0		0
405	0		0
406	0		0

407	0	0
408	0	0
409	0	0
410	0	0
411	0	0
412	0	0
413	0	0
414	0	0
415	0	0
416	0	0
417	0	0
418	0	0
419	0	0
420	0	0
421	0	0
422	0	0
423	0	0
424	0	0
425	0	0
426	0	0
427	0	0
428	0	0
429	0	0
430	0	0
431	0	0
432	0	0
433	0	1
434	0	0
435	0	0
436	0	0
437	0	0
438	0	0
439	0	0
440	0	0
441	0	0
442	0	0
443	0	0
444	0	0
445	0	1
446	0	0
447	0	0
448	0	0
449	0	0
450	0	0
451	0	0
452	0	0
453	0	0

454	0	0
455	0	0
456	0	0
457	0	0
458	0	0
459	0	0
460	0	0
461	0	0
462	0	0
463	0	0
464	0	0
465	0	0
466	0	0
467	0	0
468	0	0
469	0	0
470	0	0
471	0	0
472	0	0
473	0	0
474	0	0
475	0	0
476	0	0
477	0	0
478	0	0
479	0	0
480	0	0
481	0	0
482	0	0
483	0	0
484	0	0
485	0	0
486	0	0
487	0	0
488	1	0
489		0
490		0
491		0
492		0
493		0
494		0
495		0
496		0
497		0
498		0
499		0
500		0

501			0
502			0
503			0
504			0
505			1

	Dill	Root	Plant 2
Diameter (nm)	Run 1 Frequency	Run 2 Frequency	Run 3 Frequency
30	0	0	0
31	0	0	0
32	0	0	0
33	0	0	0
34	0	0	0
35	0	0	0
36	0	0	0
37	0	0	0
38	62	111	67
39	0	0	0
40	0	0	0
41	0	0	0
42	0	0	0
43	0	0	0
44	122	166	101
45	0	0	0
46	0	0	0
47	0	0	0
48	117	148	86
49	0	0	0
50	0	0	0
51	0	0	0
52	94	123	63
53	0	0	0
54	0	0	0
55	70	70	45
56	0	0	0
57	0	0	0
58	47	51	40
59	0	0	0
60	0	0	0
61	46	27	29
62	0	0	0
63	35	23	30
64	0	0	0

65	21	8	18
66	0	0	0
67	0	0	0
68	20	18	18
69	0	0	0
70	10	10	15
71	10	18	5
72	0	0	0
73	7	10	5
74	0	0	0
75	7	10	8
76	0	0	0
77	8	7	6
78	5	8	10
79	0	0	0
80	3	6	6
81	6	2	5
82	5	5	5
83	0	0	0
84	4	2	1
85	2	3	5
86	6	0	5
87	0	0	0
88	5	1	3
89	4	0	4
90	2	3	2
91	2	4	1
92	1	0	3
93	3	0	1
94	3	2	1
95	2	3	3
96	2	1	1
97	3	1	2
98	1	1	1
99	1	0	1
100	2	2	1
101	2	0	1
102	0	1	2
103	4	1	1
104	1	1	1
105	0	1	1
106	2	1	3
107	2	1	0
108	2	2	4
109	2	1	1
110	1	0	1
111	1	0	0

112	0	3	1
113	2	2	0
114	0	0	1
115	1	0	1
116	1	1	0
117	2	0	0
118	0	0	0
119	0	0	3
120	1	2	3
121	0	0	0
122	1	0	1
123	1	2	1
124	0	1	0
125	0	1	2
126	1	1	1
127	0	2	0
128	0	0	0
129	3	1	0
130	0	1	3
131	0	0	0
132	2	0	0
133	0	0	0
134	0	0	0
135	0	1	0
136	0	2	0
137	1	1	3
138	0	0	1
139	0	1	0
140	2	2	0
141	0	0	0
142	1	1	1
143	1	1	0
144	0	0	0
145	1	0	0
146	0	1	0
147	2	2	0
148	0	0	1
149	0	0	0
150	1	1	0
151	1	0	0
152	1	1	1
153	1	0	0
154	0	1	0
155	1	1	0
156	0	0	0
157	0	0	0
158	0	0	0

159	0	0	0
160	0	0	0
161	3	0	0
162	0	0	1
163	0	0	1
164	0	1	1
165	4	0	0
166	1	0	0
167	0	1	1
168	0	0	0
169	0	1	0
170	0	0	0
171	0	0	1
172	2	0	0
173	1	0	0
174	0	0	0
175	1	0	0
176	1	1	0
177	0	0	0
178	0	0	1
179	0	0	0
180	1	0	0
181	1	0	0
182	0	0	0
183	0	0	1
184	0	1	0
185	0	0	0
186	0	0	0
187	0	0	0
188	0	0	0
189	1	0	1
190	0	0	1
191	0	1	0
192	0	0	0
193	0	0	0
194	0	1	0
195	0	0	0
196	0	0	0
197	0	0	0
198	1	0	0
199	1	0	0
200	0	0	0
201	2	0	0
202	0	0	0
203	0	0	1
204	1	0	0
205	0	0	0

206	1	0	0
207	0	0	1
208	0	0	0
209	0	0	0
210	0	0	0
211	0	0	0
212	0	0	0
213	0	0	0
214	0	1	0
215	0	0	0
216	0	0	0
217	1	0	0
218	0	0	0
219	0	0	0
220	0	0	0
221	0	0	0
222	0	0	0
223	0	0	1
224	0	0	0
225	1	0	0
226	0	0	1
227	0	1	0
228	0	0	1
229	0	0	0
230	0	1	1
231	0	0	0
232	0	0	1
233	0	0	2
234	0	0	0
235	0	0	1
236	0	0	0
237	0	1	1
238	1	0	1
239	0	2	0
240	0	1	0
241	1	0	0
242	0	0	0
243	0	0	0
244	0	0	0
245	0	0	0
246	1	0	0
247	0	0	1
248	0	0	1
249	0	1	1
250	0	0	0
251	0	0	0
252	0	0	0

253	0	0	1
254	0	0	0
255	0	1	1
256	1	0	0
257	0	1	1
258	0	0	1
259	1	0	0
260	0	0	0
261	0	0	0
262	0	0	0
263	0	0	0
264	1	0	0
265	0	0	0
266	0	0	1
267	0	0	0
268	0	0	0
269	0	0	0
270	1	0	1
271	0	0	0
272	0	0	0
273	0	0	0
274	0	0	0
275	0	0	0
276	0	0	0
277	0	0	0
278	0	0	0
279	0	0	0
280	0	0	0
281	0	0	0
282	0	0	0
283	0	0	0
284	0	0	0
285	0	0	0
286	0	0	0
287	0	0	0
288	0	0	0
289	0	0	0
290	0	0	0
291	0	0	0
292	0	0	0
293	0	0	0
294	0	0	0
295	0	0	2
296	0	0	0
297	0	1	0
298	0	0	0
299	0	0	0

300	0	0	0
301	0	0	1
302	0	0	0
303	0	0	0
304	0	0	0
305	0	0	0
306	0	0	0
307	0	0	0
308	0	0	0
309	0	0	0
310	0	0	0
311	0	0	1
312	0	0	0
313	0	0	0
314	0	0	0
315	0	0	0
316	0	0	0
317	0	0	0
318	0	0	0
319	0	0	1
320	0	0	0
321	0	0	0
322	0	0	0
323	0	0	0
324	0	0	1
325	0	0	0
326	0	0	0
327	0	0	1
328	0	0	0
329	0	0	0
330	0	0	1
331	0	1	0
332	0	0	0
333	0	0	0
334	2	0	0
335	0	0	0
336	0	0	0
337	0	0	0
338	0	0	0
339	0	0	0
340	0	0	1
341	0	0	0
342	0	0	0
343	0	0	0
344	0	0	0
345	0	0	0
346	0	0	0

347	0	0	0
348	0	0	0
349	0	1	1
350	0	0	0
351	0	0	0
352	0	0	0
353	0	0	0
354	0	0	0
355	1	0	0
356	0	0	0
357	0	0	0
358	0	0	0
359	0	0	0
360	0	0	0
361	0	0	0
362	0	0	0
363	0	0	0
364	0	0	0
365	0	0	0
366	0	0	0
367	0	0	0
368	0	0	0
369	0	0	1
370	0	0	0
371	0	0	0
372	0	0	0
373	0	0	0
374	0	0	0
375	0	0	0
376	0	0	0
377	0	0	0
378	0	0	0
379	0	0	0
380	0	0	0
381	0	0	0
382	1	0	0
383	0	0	0
384	0	0	0
385	0	0	0
386	0	0	0
387	0	0	0
388	0	0	0
389	0	0	0
390	0	0	0
391	0	0	0
392	0	0	0
393	0	0	0

394	0	0	0
395	0	0	0
396	0	0	0
397	0	0	0
398	0	0	0
399	0	0	0
400	0	0	0
401	0	0	0
402	0	0	0
403	0	0	0
404	0	0	0
405	0	0	0
406	0	0	0
407	0	0	0
408	0	0	0
409	0	0	1
410	0	0	0
411	0	0	0
412	0	0	0
413	0	0	1
414	0	0	0
415	0	0	0
416	0	0	0
417	0	0	0
418	0	0	0
419	0	0	0
420	0	0	0
421	0	0	0
422	0	0	0
423	0	0	0
424	0	0	0
425	0	0	0
426	0	0	0
427	0	0	0
428	0	0	0
429	0	0	0
430	0	0	0
431	0	0	0
432	0	0	0
433	0	0	0
434	0	0	0
435	0	0	0
436	0	0	0
437	0	0	0
438	0	0	0
439	0	0	0
440	0	0	0

441	0	0	0
442	0	0	0
443	0	0	0
444	0	0	0
445	0	0	0
446	0	0	0
447	0	0	0
448	0	0	0
449	0	0	0
450	0	0	0
451	0	0	0
452	0	0	0
453	0	0	0
454	0	0	0
455	0	0	0
456	0	0	0
457	0	0	0
458	0	0	0
459	0	0	0
460	0	0	0
461	0	0	0
462	0	0	0
463	0	0	0
464	0	0	0
465	0	0	0
466	0	0	0
467	0	0	0
468	0	0	0
469	0	0	0
470	0	0	0
471	0	0	0
472	0	0	0
473	0	0	0
474	0	0	0
475	0	0	0
476	0	0	0
477	0	0	0
478	0	0	0
479	0	0	0
480	0	0	0
481	0	0	0
482	0	0	0
483	0	0	0
484	0	0	0
485	0	0	0
486	0	0	0
487	1	0	0

488	0	0
489	0	0
490	0	0
491	0	0
492	0	0
493	0	0
494	0	0
495	0	0
496	0	0
497	0	0
498	0	0
499	0	0
500	0	0
501	0	0
502	0	0
503	0	0
504	0	0
505	0	0
506	0	0
507	0	0
508	0	0
509	0	0
510	0	0
511	0	0
512	0	0
513	0	0
514	0	0
515	0	0
516	0	0
517	0	0
518	0	0
519	0	0
520	0	0
521	0	0
522	0	0
523	0	0
524	0	0
525	0	0
526	0	0
527	0	0
528	0	0
529	1	0
530		0
531		1

Dill Root Plant 3

Diameter (nm)	Run 1 Frequency	Run 2 Frequency	Run 3 Frequency
30	0	0	0
31	0	0	0
32	0	0	0
33	0	0	0
34	0	0	0
35	0	0	0
36	0	0	0
37	0	0	0
38	9	2	7
39	0	0	0
40	0	0	0
41	0	0	0
42	0	0	0
43	0	0	0
44	24	4	11
45	0	0	0
46	0	0	0
47	0	0	0
48	12	9	9
49	0	0	0
50	0	0	0
51	0	0	0
52	15	8	12
53	0	0	0
54	0	0	0
55	22	7	9
56	0	0	0
57	0	0	0
58	12	10	14
59	0	0	0
60	0	0	0
61	13	15	14
62	0	0	0
63	10	7	5
64	0	0	0
65	5	11	5
66	0	0	0
67	0	0	0
68	5	5	4
69	0	0	0
70	2	9	9
71	4	2	3
72	0	0	0
73	3	3	3

74	0	0	0
75	4	1	1
76	0	0	0
77	3	2	2
78	0	4	2
79	0	0	0
80	1	2	2
81	2	1	1
82	1	2	2
83	0	0	0
84	1	5	4
85	3	1	1
86	2	2	0
87	0	0	0
88	0	0	2
89	3	0	1
90	1	0	1
91	2	2	1
92	1	3	2
93	1	2	2
94	2	1	1
95	1	2	0
96	3	1	1
97	0	1	2
98	1	0	0
99	0	2	0
100	1	0	2
101	0	0	0
102	1	1	0
103	0	0	0
104	0	0	0
105	0	0	1
106	0	2	2
107	0	0	0
108	1	1	2
109	0	1	1
110	1	0	2
111	0	0	0
112	1	0	1
113	2	0	1
114	0	1	3
115	0	0	0
116	0	0	0
117	0	0	1
118	2	1	1
119	1	0	1
120	0	0	0

121	0	0	0
122	0	1	0
123	1	0	0
124	1	0	0
125	0	1	1
126	1	0	1
127	0	0	0
128	0	1	0
129	0	0	1
130	0	1	1
131	0	0	0
132	0	0	1
133	1	0	0
134	0	0	1
135	2	1	0
136	0	1	0
137	0	0	0
138	0	0	0
139	1	0	0
140	0	0	3
141	0	0	0
142	0	0	1
143	0	0	0
144	0	2	0
145	0	0	1
146	1	0	0
147	0	1	0
148	0	2	0
149	0	1	2
150	0	0	1
151	0	0	0
152	0	0	1
153	1	0	0
154	0	0	0
155	1	0	1
156	0	0	0
157	1	1	1
158	0	0	0
159	0	1	0
160	0	0	0
161	1	0	0
162	1	0	1
163	0	0	1
164	0	0	0
165	0	1	0
166	0	0	0
167	1	0	1

168	0	1	0
169	0	0	1
170	2	0	0
171	0	0	0
172	0	0	0
173	1	0	1
174	1	0	0
175	0	0	1
176	0	0	0
177	0	0	0
178	0	0	0
179	0	0	0
180	0	0	0
181	0	0	0
182	0	0	0
183	1	0	0
184	0	0	0
185	0	0	0
186	0	0	0
187	0	0	1
188	0	0	0
189	1	2	0
190	0	0	0
191	0	0	0
192	0	0	0
193	0	1	0
194	0	0	0
195	0	1	0
196	0	0	3
197	0	0	0
198	0	0	0
199	2	0	0
200	0	0	0
201	0	0	0
202	0	0	0
203	0	0	0
204	1	1	0
205	1	0	0
206	0	1	0
207	1	0	0
208	0	0	0
209	1	0	0
210	0	0	0
211	0	1	0
212	0	0	0
213	0	2	0
214	0	0	1

215	0	0	0
216	0	0	0
217	0	0	0
218	0	1	0
219	0	0	1
220	0	0	0
221	0	0	0
222	0	0	0
223	0	0	0
224	1	0	0
225	0	0	0
226	0	0	0
227	1	1	0
228	0	0	0
229	0	0	0
230	0	0	0
231	0	0	2
232	0	0	2
233	0	0	0
234	0	0	0
235	0	0	1
236	0	0	0
237	0	0	1
238	0	0	0
239	0	0	0
240	1	0	0
241	0	0	0
242	0	0	0
243	0	0	0
244	0	0	0
245	1	0	0
246	0	0	0
247	0	0	0
248	0	0	1
249	0	0	0
250	0	0	0
251	0	0	0
252	0	0	0
253	0	0	0
254	0	0	0
255	0	0	0
256	0	0	0
257	0	0	0
258	0	0	0
259	0	0	0
260	0	0	0
261	0	0	0

262	0	1	0
263	0	0	0
264	1	0	0
265	0	0	0
266	0	0	0
267	0	0	0
268	0	1	0
269	0	1	1
270	0	0	0
271	0	0	0
272	0	0	0
273	1	0	0
274	0	0	0
275	0	0	0
276	0	0	0
277	0	0	0
278	0	0	0
279	0	0	0
280	1	0	0
281	0	0	0
282	0	1	0
283	0	0	0
284	0	0	0
285	0	0	0
286	0	0	0
287	1	0	0
288	0	0	0
289	0	1	0
290	0	1	0
291	0	0	0
292	0	0	0
293	0	0	0
294	1	0	0
295	0	0	0
296	0	0	1
297	0	0	0
298	0	0	0
299	0	0	0
300	0	0	0
301	0	0	0
302	0	0	0
303	0	0	0
304	0	0	0
305	0	0	0
306	0	0	0
307	0	0	0
308	0	0	0

309	0	0	0
310	0	0	0
311	0	0	0
312	0	0	0
313	0	0	0
314	0	0	0
315	0	0	0
316	0	0	0
317	0	0	0
318	0	0	0
319	0	0	0
320	0	0	0
321	0	1	0
322	0	0	0
323	0	0	0
324	0	1	0
325	0		0
326	0		0
327	0		0
328	0		0
329	0		0
330	0		0
331	0		0
332	0		0
333	0		0
334	0		0
335	0		0
336	0		0
337	0		0
338	0		0
339	0		0
340	0		0
341	0		0
342	0		1
343	0		0
344	0		0
345	0		1
346	0		0
347	0		0
348	0		0
349	0		0
350	0		0
351	0		0
352	0		0
353	0		0
354	0		0
355	0		0

356	0	0
357	0	0
358	0	0
359	0	0
360	0	0
361	0	0
362	0	0
363	0	0
364	0	0
365	0	0
366	0	1
367	0	0
368	0	0
369	0	0
370	0	0
371	0	0
372	0	0
373	0	0
374	0	0
375	0	0
376	0	0
377	0	0
378	0	0
379	0	0
380	0	0
381	0	0
382	0	0
383	0	0
384	0	0
385	0	0
386	0	0
387	0	1
388	0	
389	0	
390	0	
391	0	
392	0	
393	0	
394	0	
395	0	
396	0	
397	0	
398	0	
399	0	
400	0	
401	0	
402	0	

403	0
404	0
405	0
406	0
407	0
408	0
409	0
410	0
411	0
412	0
413	0
414	0
415	0
416	0
417	0
418	0
419	0
420	0
421	0
422	0
423	0
424	1

	Dill	Shoot	Plant 1
	Run 1	Run 2	Run 3
Diameter (nm)	Frequency	Frequency	Frequency
30	0	0	0
31	0	0	0
32	0	0	0
33	0	0	0
34	0	0	0
35	0	0	0
36	0	0	0
37	0	0	0
38	12	21	3
39	0	0	0
40	0	0	0
41	0	0	0
42	0	0	0
43	0	0	0
44	22	16	10
45	0	0	0
46	0	0	0
47	0	0	0
48	27	21	11

49	0	0	0
50	0	0	0
51	0	0	0
52	15	12	11
53	0	0	0
54	0	0	0
55	9	12	6
56	0	0	0
57	0	0	0
58	6	10	7
59	0	0	0
60	0	0	0
61	3	6	7
62	0	0	0
63	2	1	3
64	0	0	0
65	1	2	6
66	0	0	0
67	0	0	0
68	2	2	2
69	0	0	0
70	0	1	3
71	0	3	0
72	0	0	0
73	1	1	1
74	0	0	0
75	0	1	0
76	0	0	0
77	2	0	0
78	0	0	0
79	0	0	0
80	0	0	0
81	1	1	0
82	0	0	0
83	0	0	0
84	0	1	1
85	1	0	3
86	0	0	1
87	0	0	0
88	0	0	0
89	0	0	0
90	0	0	0
91	1	0	2
92	0	0	0
93	0	0	0
94	1	1	1
95	0	1	2

96	0	0	0
97	0	0	0
98	0	2	1
99	1	0	0
100	0	0	0
101	0	0	0
102	1	0	1
103	0	0	1
104	2	0	1
105	0	1	0
106	0	0	0
107	0	0	0
108	1	0	0
109	0	0	0
110	0	0	1
111	0	0	0
112	0	0	0
113	0	0	0
114	0	0	0
115	0	1	1
116	0	0	0
117	0	0	0
118	0	0	1
119	1	0	1
120	0	0	0
121	0	0	0
122	0	0	0
123	0	0	0
124	2	0	0
125	0	0	1
126	0	1	0
127	0	0	0
128	0	0	1
129	0	1	0
130	0	1	0
131	0	0	0
132	2	0	1
133	0	1	0
134	0	0	0
135	0	0	0
136	0	0	0
137	0	0	0
138	0	1	0
139	0	0	0
140	0	0	0
141	1	0	0
142	0	0	0

143	0	0	0
144	0	0	0
145	0	0	0
146	0	0	1
147	0	0	0
148	1	0	0
149	0	0	2
150	1	0	0
151	0	0	0
152	1	0	0
153	0	0	0
154	0	0	0
155	0	0	0
156	0	0	0
157	1	1	1
158	0	0	0
159	0	0	0
160	0	0	2
161	0	0	0
162	0	1	0
163	0	0	1
164	0	1	0
165	0	0	0
166	0	0	0
167	1	0	0
168	1	0	0
169	1	0	0
170	0	0	0
171	0	0	0
172	0	0	0
173	0	0	1
174	0	0	0
175	0	0	0
176	0	0	0
177	0	2	0
178	0	0	0
179	0	0	0
180	0	0	0
181	0	1	1
182	0	0	1
183	0	0	0
184	0	0	0
185	0	0	0
186	0	0	0
187	0	0	0
188	0	0	0
189	0	0	0

190	0	0	0
191	0	0	0
192	0	0	0
193	1	0	1
194		0	1
195		0	0
196		0	0
197		0	0
198		0	2
199		0	0
200		0	0
201		0	0
202		0	0
203		0	0
204		0	1
205		0	0
206		0	0
207		0	0
208		0	0
209		0	0
210		0	0
211		0	0
212		0	0
213		0	0
214		0	0
215		0	0
216		0	0
217		0	0
218		0	0
219		0	0
220		0	0
221		0	0
222		0	0
223		0	0
224		0	0
225		0	0
226		0	0
227		0	0
228		0	0
229		0	0
230		0	0
231		0	0
232		0	0
233		0	0
234		0	0
235		0	0
236		0	0

237	0	0
238	0	0
239	0	0
240	0	0
241	0	0
242	0	0
243	0	0
244	0	0
245	0	0
246	0	0
247	0	0
248	0	0
249	0	0
250	0	0
251	0	0
252	0	0
253	0	0
254	0	0
255	0	0
256	0	0
257	0	0
258	0	0
259	0	0
260	0	0
261	0	0
262	0	0
263	0	0
264	0	0
265	0	0
266	1	1
267		0
268		0
269		0
270		0
271		0
272		0
273		0
274		0
275		0
276		0
277		0
278		0
279		0
280		0
281		0
282		0
283		0

284	0
285	0
286	0
287	0
288	0
289	0
290	0
291	0
292	0
293	0
294	0
295	0
296	0
297	0
298	0
299	0
300	0
301	0
302	0
303	0
304	0
305	0
306	0
307	0
308	0
309	0
310	0
311	0
312	0
313	0
314	0
315	0
316	0
317	0
318	0
319	0
320	0
321	0
322	0
323	0
324	0
325	0
326	0
327	0
328	0
329	0
330	0

331	0
332	0
333	0
334	0
335	0
336	0
337	0
338	0
339	0
340	0
341	0
342	0
343	0
344	0
345	0
346	0
347	0
348	0
349	0
350	0
351	0
352	0
353	0
354	0
355	0
356	0
357	0
358	0
359	0
360	0
361	0
362	0
363	0
364	0
365	0
366	0
367	0
368	0
369	0
370	0
371	0
372	0
373	0
374	0
375	0
376	0
377	0

378	0
379	0
380	0
381	0
382	0
383	0
384	0
385	0
386	0
387	0
388	0
389	0
390	0
391	0
392	0
393	0
394	0
395	0
396	0
397	0
398	0
399	0
400	1

	Dill	Shoot	Plant 2
	Run 1	Run 2	Run 3
Diameter (nm)	Frequency	Frequency	Frequency
30	0	0	0
31	0	0	0
32	0	0	0
33	0	0	0
34	0	0	0
35	0	0	0
36	0	0	0
37	0	0	0
38	0	0	4
39	0	0	0
40	0	0	0
41	0	0	0
42	0	0	0
43	0	0	0
44	7	16	7
45	0	0	0
46	0	0	0
47	0	0	0

48	10	10	11
49	0	0	0
50	0	0	0
51	0	0	0
52	10	14	12
53	0	0	0
54	0	0	0
55	17	20	17
56	0	0	0
57	0	0	0
58	18	18	10
59	0	0	0
60	0	0	0
61	19	22	24
62	0	0	0
63	12	14	14
64	0	0	0
65	10	11	11
66	0	0	0
67	0	0	0
68	10	9	13
69	0	0	0
70	7	8	9
71	9	9	6
72	0	0	0
73	6	5	11
74	0	0	0
75	9	4	5
76	0	0	0
77	7	4	6
78	11	5	11
79	0	0	0
80	3	2	4
81	7	5	5
82	3	2	4
83	0	0	0
84	3	5	3
85	2	1	4
86	7	3	6
87	0	0	0
88	4	4	3
89	5	4	0
90	3	2	1
91	4	2	3
92	3	3	1
93	2	3	1
94	1	0	2

95	2	2	3
96	0	3	1
97	2	2	2
98	2	1	3
99	1	1	1
100	2	1	6
101	1	2	0
102	3	2	2
103	2	1	1
104	0	2	2
105	2	1	1
106	4	3	1
107	1	2	0
108	1	1	0
109	3	1	0
110	3	3	1
111	0	1	1
112	1	2	3
113	5	2	2
114	0	0	0
115	0	2	0
116	1	0	2
117	0	0	0
118	0	4	2
119	0	0	1
120	0	3	0
121	0	0	1
122	4	1	1
123	2	0	1
124	0	0	1
125	1	0	0
126	0	1	2
127	0	1	3
128	0	1	0
129	1	1	0
130	1	1	1
131	4	1	2
132	2	0	0
133	0	1	0
134	1	1	3
135	1	1	0
136	1	0	1
137	0	3	2
138	1	0	0
139	0	0	1
140	0	2	1
141	0	4	2

142	0	1	1
143	1	2	1
144	4	0	1
145	1	0	0
146	1	1	1
147	0	2	2
148	0	1	0
149	1	0	2
150	0	2	0
151	1	0	0
152	1	1	0
153	0	0	1
154	0	2	3
155	1	1	0
156	5	0	1
157	2	1	2
158	1	1	4
159	1	0	0
160	1	0	2
161	1	0	0
162	1	2	0
163	1	1	0
164	1	0	0
165	1	1	2
166	1	2	0
167	0	0	1
168	1	0	0
169	0	1	1
170	0	0	0
171	0	1	0
172	0	0	0
173	0	1	0
174	0	0	3
175	1	1	0
176	0	2	0
177	0	0	0
178	0	1	1
179	0	0	1
180	1	0	0
181	0	1	0
182	0	0	0
183	1	0	0
184	0	0	0
185	0	3	1
186	0	0	0
187	0	1	0
188	0	1	1

189	2	0	0
190	0	1	0
191	0	0	0
192	0	0	0
193	0	0	0
194	1	0	0
195	1	0	0
196	0	1	1
197	0	0	0
198	1	1	0
199	0	0	1
200	1	1	0
201	0	1	0
202	1	0	0
203	0	1	0
204	1	0	0
205	1	0	0
206	0	0	0
207	1	0	0
208	0	0	0
209	0	1	2
210	0	0	0
211	0	0	2
212	0	0	0
213	0	0	0
214	0	1	0
215	1	0	0
216	0	0	0
217	1	1	0
218	0	0	0
219	0	1	1
220	1	0	1
221	1	0	0
222	0	0	0
223	1	0	1
224	0	0	0
225	0	1	0
226	1	1	0
227	1	0	0
228	0	1	0
229	0	1	0
230	0	0	0
231	0	0	0
232	1	0	0
233	1	0	0
234	0	0	0
235	0	0	1

236	0	0	0
237	0	0	0
238	0	0	0
239	0	0	0
240	1	0	0
241	0	1	0
242	0	0	0
243	0	0	0
244	0	0	0
245	1	0	0
246	0	1	0
247	0	0	0
248	0	1	0
249	0	0	0
250	0	0	0
251	0	0	0
252	0	0	0
253	0	0	0
254	0	0	0
255	0	0	1
256	0	0	1
257	0	1	0
258	1	0	0
259	0	0	0
260	0	0	0
261	1	0	0
262	0	0	0
263	0	0	0
264	0	0	0
265	0	1	0
266	0	1	0
267	0	1	1
268	0	0	0
269	0	0	0
270	0	1	0
271	0	0	0
272	0	0	0
273	0	0	0
274	2	0	0
275	0	0	0
276	0	0	0
277	0	0	0
278	0	0	0
279	0	0	0
280	0	0	0
281	0	0	0
282	0	0	0

283	0	1	0
284	0	0	0
285	1	0	0
286	1	0	0
287	0	0	0
288	0	0	0
289	0	0	0
290	0	0	0
291	0	0	0
292	0	0	1
293	0	0	0
294	0	0	0
295	0	0	0
296	0	0	0
297	0	0	0
298	0	0	0
299	0	0	0
300	0	0	0
301	0	0	0
302	0	0	0
303	0	0	0
304	0	0	0
305	0	0	0
306	0	0	0
307	0	0	0
308	1	0	0
309	0	0	0
310	0	0	0
311	0	0	1
312	0	0	0
313	1	0	0
314	0	0	0
315	0	0	0
316	0	0	0
317	0	0	0
318	0	0	0
319	0	0	0
320	0	0	0
321	0	0	0
322	0	0	0
323	0	0	0
324	0	0	0
325	0	0	0
326	0	0	0
327	0	0	0
328	0	0	0
329	0	0	0

330	0	0	1
331	0	0	0
332	0	0	0
333	0	0	0
334	0	0	1
335	0	0	
336	0	0	
337	0	0	
338	0	0	
339	1	0	
340	0	0	
341	0	0	
342	0	0	
343	0	0	
344	0	0	
345	0	0	
346	1	0	
347		0	
348		0	
349		0	
350		0	
351		1	

	Dill	Shoot	Plant 3
	Run 1	Run 2	Run 3
Diameter (nm)	Frequency	Frequency	Frequency
30	0	0	0
31	0	0	0
32	0	0	0
33	0	0	0
34	0	0	0
35	0	0	0
36	0	0	0
37	0	0	0
38	1	2	0
39	0	0	0
40	0	0	0
41	0	0	0
42	0	0	0
43	0	0	0
44	12	5	6
45	0	0	0
46	0	0	0
47	0	0	0
48	5	3	7

49	0	0	0
50	0	0	0
51	0	0	0
52	12	5	3
53	0	0	0
54	0	0	0
55	11	6	4
56	0	0	0
57	0	0	0
58	8	9	5
59	0	0	0
60	0	0	0
61	5	6	5
62	0	0	0
63	7	6	8
64	0	0	0
65	9	8	8
66	0	0	0
67	0	0	0
68	5	6	9
69	0	0	0
70	2	9	3
71	2	1	5
72	0	0	0
73	3	1	8
74	0	0	0
75	3	4	3
76	0	0	0
77	3	1	3
78	3	3	3
79	0	0	0
80	2	0	2
81	1	0	0
82	0	2	0
83	0	0	0
84	0	1	1
85	1	2	0
86	1	1	2
87	0	0	0
88	3	2	1
89	0	3	1
90	1	1	1
91	1	0	1
92	1	0	1
93	2	1	1
94	0	0	1
95	1	2	1

96	1	2	1
97	0	0	1
98	0	1	1
99	2	1	0
100	1	1	0
101	1	0	3
102	0	0	1
103	1	0	0
104	1	0	0
105	0	0	1
106	2	1	2
107	0	1	1
108	1	1	0
109	0	0	0
110	2	2	0
111	0	1	1
112	0	0	0
113	2	0	0
114	1	0	0
115	0	1	0
116	2	1	2
117	0	1	2
118	1	1	1
119	0	0	1
120	1	1	1
121	0	0	2
122	1	1	1
123	2	0	1
124	0	0	1
125	1	0	2
126	0	0	0
127	1	0	0
128	1	1	0
129	0	1	0
130	0	0	0
131	0	0	0
132	0	0	0
133	0	0	0
134	0	0	2
135	0	0	2
136	1	1	1
137	0	0	2
138	1	0	2
139	0	0	0
140	0	1	1
141	1	1	1
142	0	1	0

143	0	2	0
144	1	0	0
145	0	0	1
146	1	0	1
147	0	1	0
148	0	0	0
149	0	0	0
150	0	0	0
151	2	0	0
152	0	2	1
153	0	0	0
154	0	0	0
155	0	2	1
156	1	0	0
157	0	0	1
158	0	0	0
159	1	1	0
160	0	0	0
161	1	0	0
162	1	0	1
163	0	0	0
164	0	0	0
165	1	1	0
166	0	0	1
167	0	2	0
168	0	0	2
169	0	0	0
170	0	0	1
171	0	0	2
172	0	0	0
173	0	0	0
174	0	0	0
175	1	2	1
176	0	0	0
177	0	0	1
178	0	2	0
179	0	0	1
180	0	1	0
181	0	0	0
182	0	0	0
183	0	0	0
184	0	0	0
185	0	0	0
186	0	1	0
187	0	0	0
188	1	0	0
189	0	0	0

190	2	0	0
191	0	1	0
192	0	0	0
193	0	0	0
194	0	1	0
195	0	1	1
196	0	0	1
197	1	0	0
198	1	0	0
199	0	0	1
200	0	0	0
201	1	0	0
202	1	0	0
203	0	0	0
204	0	0	0
205	0	0	0
206	0	1	0
207	0	2	0
208	0	0	0
209	1	0	0
210	0	0	0
211	0	1	1
212	0	0	0
213	0	0	0
214	0	0	0
215	0	0	0
216	0	1	0
217	0	0	0
218	0	0	0
219	1	0	0
220	0	0	0
221	0	0	0
222	0	0	0
223	0	1	1
224	0	0	0
225	0	0	0
226	0	0	1
227	0	0	1
228	0	0	2
229	0	0	0
230	0	0	1
231	0	0	0
232	0	0	0
233	0	0	0
234	0	0	0
235	0	0	0
236	0	0	0

237	0	0	0
238	1	0	0
239	1	0	0
240	0	0	1
241	0	0	0
242	0	0	0
243	0	0	0
244	0	1	0
245	0	0	0
246	0	0	0
247	0	0	0
248	0	0	0
249	0	0	0
250	0	0	0
251	0	0	0
252	0	0	0
253	0	0	0
254	0	0	0
255	0	0	1
256	0	0	0
257	0	1	0
258	0	0	0
259	0	0	0
260	0	0	0
261	0	0	0
262	0	0	0
263	0	0	0
264	0	0	0
265	0	0	0
266	1	0	0
267	0	0	0
268	0	0	0
269	0	0	0
270	0	0	0
271	0	0	0
272	0	0	0
273	0	0	0
274	0	0	0
275	0	0	0
276	0	0	0
277	0	0	0
278	0	0	0
279	0	0	0
280	0	0	0
281	0	0	0
282	0	0	0
283	0	0	0

284	0	0	0
285	0	0	0
286	0	0	0
287	0	0	0
288	0	0	0
289	0	0	0
290	0	0	0
291	0	0	0
292	0	0	0
293	0	0	0
294	0	1	0
295	0	0	0
296	1	0	0
297	0	1	0
298	0	0	0
299	0	0	0
300	0	0	0
301	0	0	0
302	0	0	0
303	0	0	0
304	0	0	0
305	0	0	0
306	0	0	0
307	0	0	0
308	0	0	0
309	0	1	0
310	0	0	1
311	0	0	0
312	0	0	0
313	0	0	0
314	0	0	0
315	0	0	0
316	0	0	0
317	0	0	0
318	0	0	0
319	0	1	0
320	0	0	0
321	0	0	0
322	0	0	0
323	0	0	0
324	0	0	0
325	0	0	0
326	0	0	0
327	0	0	0
328	0	0	0
329	0	0	0
330	0	0	0

331	0	0	0
332	0	0	0
333	0	0	0
334	0	0	0
335	0	0	0
336	0	0	0
337	0	0	0
338	0	0	1
339	1	0	0
340	0	0	0
341	0	0	0
342	0	0	0
343	1	0	0
344	0	0	0
345	0	0	0
346	0	0	0
347	0	0	0
348	0	0	0
349	0	0	0
350	0	0	0
351	0	0	0
352	0	0	0
353	0	0	0
354	0	1	0
355	0	0	0
356	0	0	0
357	0	0	0
358	0	0	0
359	0	1	0
360	0	0	0
361	0	0	0
362	0	0	0
363	0	0	0
364	0	0	0
365	0	0	0
366	0	0	0
367	0	0	0
368	0	0	0
369	0	0	0
370	0	0	0
371	0	0	0
372	0	0	0
373	0	0	0
374	0	0	0
375	0	0	0
376	0	1	0
377	0	0	0

378	0	0	0
379	0	0	0
380	1	0	0
381	0	0	0
382	0	0	0
383	0	0	0
384	0	0	0
385	0	0	0
386	0	0	0
387	0	0	0
388	0	0	0
389	0	0	0
390	0	0	0
391	0	0	0
392	0	0	0
393	0	0	0
394	0	0	0
395	0	0	0
396	0	0	0
397	0	0	0
398	0	0	0
399	1	0	0
400		0	0
401		0	0
402		0	0
403		0	0
404		0	0
405		0	0
406		0	0
407		0	0
408		0	0
409		0	0
410		0	0
411		0	0
412		0	0
413		0	0
414		0	0
415		0	0
416		0	0
417		0	0
418		0	0
419		0	0
420		0	0
421		0	0
422		0	0
423		0	0
424		0	0

425	0	0
426	0	0
427	0	0
428	0	0
429	0	0
430	0	0
431	0	0
432	0	0
433	0	0
434	0	0
435	0	0
436	0	0
437	0	0
438	0	0
439	0	0
440	0	0
441	0	0
442	0	0
443	0	0
444	0	0
445	0	0
446	0	0
447	0	0
448	0	0
449	0	0
450	0	0
451	0	0
452	0	0
453	0	0
454	0	0
455	0	0
456	0	0
457	0	0
458	0	0
459	0	0
460	0	0
461	0	0
462	0	0
463	1	0
464		0
465		0
466		0
467		0
468		0
469		0
470		0
471		0

472	0
473	0
474	0
475	0
476	0
477	0
478	0
479	0
480	0
481	0
482	0
483	0
484	0
485	0
486	0
487	0
488	0
489	0
490	0
491	0
492	0
493	0
494	0
495	0
496	0
497	0
498	1

	Chard	Root	Plant 1
	Run 1	Run 2	Run 3
Diameter (nm)	Frequency	Frequency	Frequency
36	0	0	0
37	0	0	0
38	0	0	0
39	0	0	0
40	0	0	0
41	0	0	0
42	0	0	0
43	0	0	0
44	0	0	0
45	69	40	70
46	0	0	0
47	0	0	0
48	0	0	0
49	0	0	0

50	0	0	0
51	0	0	0
52	189	155	138
53	0	0	0
54	0	0	0
55	0	0	0
56	0	0	0
57	222	198	180
58	0	0	0
59	0	0	0
60	0	0	0
61	0	0	0
62	286	293	283
63	0	0	0
64	0	0	0
65	132	142	125
66	0	0	0
67	0	0	0
68	0	0	0
69	61	54	69
70	0	0	0
71	0	0	0
72	38	28	30
73	0	0	0
74	0	0	0
75	11	19	5
76	0	0	0
77	0	0	0
78	14	12	11
79	0	0	0
80	7	2	7
81	0	0	0
82	3	4	6
83	0	0	0
84	0	0	0
85	1	5	3
86	0	0	0
87	1	0	2
88	0	0	0
89	1	3	3
90	0	0	0
91	0	1	0
92	0	0	0
93	0	0	1
94	1	0	0
95	0	0	0
96	0	2	1

97	0	0	0
98	0	0	0
99	0	0	0
100	0	0	0
101	0	0	0
102	0	1	0
103	0	0	0
104	0	0	0
105	0	0	1
106	0	0	0
107	0	0	0
108	1	0	1
109	0	1	0
110	0	0	0
111	0	0	0
112	1	1	0
113	0	0	0
114	0	0	0
115	0	0	0
116	0	0	1
117	0	0	
118	1	0	
119	2	0	
120	0	0	
121	0	0	
122	1	0	
123	0	1	
124	0	0	
125	0	0	
126	0	0	
127	0	0	
128	0	0	
129	1	1	
130	0	0	
131	0	0	
132	1	0	
133		0	
134		0	
135		0	
136		0	
137		0	
138		0	
139		0	
140		0	
141		1	

	Chard	Root	Plant 2
	Run 1	Run 2	Run 3
Diameter (nm)	Frequency	Frequency	Frequency
36	0	0	0
37	0	0	0
38	0	0	0
39	0	0	0
40	0	0	0
41	0	0	0
42	0	0	0
43	0	0	0
44	0	0	0
45	71	61	66
46	0	0	0
47	0	0	0
48	0	0	0
49	0	0	0
50	0	0	0
51	0	0	0
52	195	183	178
53	0	0	0
54	0	0	0
55	0	0	0
56	0	0	0
57	254	237	248
58	0	0	0
59	0	0	0
60	0	0	0
61	0	0	0
62	369	309	327
63	0	0	0
64	0	0	0
65	125	132	151
66	0	0	0
67	0	0	0
68	0	0	0
69	78	61	50
70	0	0	0
71	0	0	0
72	28	23	24
73	0	0	0
74	0	0	0
75	11	4	13
76	0	0	0
77	0	0	0

78	6	5	2
79	0	0	0
80	3	7	7
81	0	0	0
82	1	1	3
83	0	0	0
84	0	0	0
85	1	3	1
86	0	0	0
87	3	1	2
88	0	0	0
89	1	0	0
90	0	0	0
91	3	1	0
92	0	0	0
93	1	1	4
94	0	1	1
95	0	0	0
96	0	1	0
97	0	0	0
98	1	0	0
99	0	0	0
100	0	0	0
101	0	0	0
102	1	2	1
103		0	0
104		1	0
105		0	0
106		0	0
107		0	0
108		1	1
109		1	
110		0	
111		0	
112		0	
113		0	
114		0	
115		0	
116		0	
117		0	
118		0	
119		1	
120		0	
121		0	
122		0	
123		0	
124		0	

125	1
126	0
127	0
128	0
129	0
130	0
131	0
132	0
133	0
134	1

	Chard	Root	Plant 3
	Run 1	Run 2	Run 3
Diameter (nm)	Frequency	Frequency	Frequency
36	0	0	0
37	0	0	0
38	0	0	0
39	0	0	0
40	0	0	0
41	0	0	0
42	0	0	0
43	0	0	0
44	0	0	0
45	1526	1373	401
46	0	0	0
47	0	0	0
48	0	0	0
49	0	0	0
50	0	0	0
51	0	0	0
52	1417	1254	446
53	0	0	0
54	0	0	0
55	0	0	0
56	0	0	0
57	1078	947	369
58	0	0	0
59	0	0	0
60	0	0	0
61	0	0	0
62	201	203	187
63	0	0	0
64	0	0	0
65	85	73	57
66	0	0	0

67	0	0	0
68	0	0	0
69	22	23	24
70	0	0	0
71	0	0	0
72	9	9	10
73	0	0	0
74	0	0	0
75	2	4	0
76	0	0	0
77	0	0	0
78	5	3	8
79	0	0	0
80	0	0	1
81	0	0	0
82	2	1	0
83	0	0	0
84	0	0	0
85	1	0	0
86	0	0	0
87	1	0	1
88	0	0	0
89	2	0	0
90	0	0	0
91	0	1	0
92	0	0	0
93	1	0	1
94	1	0	1
95	0	0	0
96	1	0	0
97	0	0	0
98	0	0	2
99	0	0	0
100	0	0	0
101	0	2	2
102	0	0	0
103	0	0	0
104	1	0	1
105	1	0	0
106	0	0	0
107	0	0	0
108	0	1	0
109	0	0	0
110	0	0	0
111	0	0	0
112	0	0	0
113	0	0	0

114	0	0	0
115	0	0	0
116	0	0	0
117	0	0	0
118	0	0	0
119	0	0	0
120	0	0	0
121	0	0	0
122	0	1	0
123	0		0
124	0		0
125	0		1
126	0		0
127	1		0
128			0
129			0
130			0
131			0
132			0
133			0
134			0
135			0
136			1

	Chard	Shoot	Plant 1
	Run 1	Run 2	Run 3
Diameter (nm)	Frequency	Frequency	Frequency
36	0	83	0
37	0	0	0
38	0	0	0
39	0	0	0
40	0	0	0
41	0	0	0
42	0	0	0
43	0	0	0
44	0	0	0
45	23	503	182
46	0	0	0
47	0	0	0
48	0	0	0
49	0	0	0
50	0	0	0
51	0	0	0
52	51	855	670
53	0	0	0

54	0	0	0
55	0	0	0
56	0	0	0
57	87	100	104
58	0	0	0
59	0	0	0
60	0	0	0
61	0	0	0
62	12	15	16
63	0	0	0
64	0	0	0
65	4	1	1
66	0	0	0
67	0	0	0
68	0	0	0
69	1	0	1
70	0	0	
71	0	0	
72	1	0	
73		0	
74		0	
75		0	
76		0	
77		0	
78		0	
79		0	
80		1	

	Chard	Shoot	Plant 2
	Run 1	Run 2	Run 3
Diameter (nm)	Frequency	Frequency	Frequency
36	0	60	0
37	0	0	0
38	0	0	0
39	0	0	0
40	0	0	0
41	0	0	0
42	0	0	0
43	0	0	0
44	0	0	0
45	201	382	165
46	0	0	0
47	0	0	0
48	0	0	0
49	0	0	0

50	0	0	0
51	0	0	0
52	711	739	653
53	0	0	0
54	0	0	0
55	0	0	0
56	0	0	0
57	103	97	89
58	0	0	0
59	0	0	0
60	0	0	0
61	0	0	0
62	14	10	15
63	0	0	0
64	0	0	0
65	1	2	2
66		0	0
67		0	0
68		0	0
69		0	1
70		0	
71		0	
72		1	
73		0	
74		0	
75		0	
76		0	
77		0	
78		0	
79		0	
80		0	
81		0	
82		0	
83		0	
84		0	
85		0	
86		0	
87		0	
88		0	
89		0	
90		0	
91		0	
92		0	
93		0	
94		0	
95		0	
96		0	

97	0
98	0
99	0
100	0
101	0
102	0
103	0
104	0
105	0
106	0
107	0
108	0
109	0
110	0
111	0
112	0
113	0
114	0
115	0
116	0
117	0
118	0
119	0
120	0
121	0
122	1

	Chard	Shoot	Plant 3
	Run 1	Run 2	Run 3
Diameter (nm)	Frequency	Frequency	Frequency
36	0	0	0
37	0	0	0
38	0	0	0
39	0	0	0
40	0	0	0
41	0	0	0
42	0	0	0
43	0	0	0
44	0	0	0
45	203	1079	163
46	0	0	0
47	0	0	0
48	0	0	0
49	0	0	0
50	0	0	0

51	0	0	0
52	395	4032	407
53	0	0	0
54	0	0	0
55	0	0	0
56	0	0	0
57	760	1069	735
58	0	0	0
59	0	0	0
60	0	0	0
61	0	0	0
62	189	284	209
63	0	0	0
64	0	0	0
65	56	59	64
66	0	0	0
67	0	0	0
68	0	0	0
69	11	13	18
70	0	0	0
71	0	0	0
72	6	4	0
73	0	0	0
74	0	0	0
75	3	1	1
76	0		
77	0		
78	1		

	Spinach	Root	Plant 1
	Run 1	Run 2	Run 3
Diameter (nm)	Frequency	Frequency	Frequency
36	0	0	0
37	0	0	0
38	0	0	0
39	0	0	0
40	0	0	0
41	0	0	0
42	0	0	0
43	0	0	0
44	0	0	0
45	238	709	595
46	0	0	0
47	0	0	0
48	0	0	0

49	0	0	0
50	0	0	0
51	0	0	0
52	283	657	622
53	0	0	0
54	0	0	0
55	0	0	0
56	0	0	0
57	265	493	426
58	0	0	0
59	0	0	0
60	0	0	0
61	0	0	0
62	143	109	75
63	0	0	0
64	0	0	0
65	47	34	34
66	0	0	0
67	0	0	0
68	0	0	0
69	13	19	14
70	0	0	0
71	0	0	0
72	5	7	6
73	0	0	0
74	0	0	0
75	2	3	3
76	0	0	0
77	0	0	0
78	3	5	3
79	0	0	0
80	5	2	1
81	0	0	0
82	0	2	3
83	0	0	0
84	0	0	0
85	1	1	1
86	0	0	0
87	2	0	1
88	0	0	0
89	1	0	2
90	0	0	0
91	2	1	1
92	0	0	0
93	0	1	0
94	1	0	1
95	0	0	0

96	0	0	2
97	0	0	0
98	1	1	0
99	3	0	1
100	0	0	0
101	0	2	0
102	1	0	0
103	0	0	0
104	0	0	0
105	1	0	1
106	0	0	0
107	1	1	0
108	0	0	0
109	0	2	0
110	0	0	0
111	0	0	0
112	0	0	0
113	0	0	0
114	1	1	0
115	0		0
116	1		0
117	0		0
118	0		0
119	0		0
120	0		0
121	0		0
122	0		0
123	0		0
124	0		0
125	0		0
126	0		0
127	0		1
128	1		0
129	0		1
130	0		
131	1		
132	0		
133	0		
134	0		
135	0		
136	0		
137	0		
138	0		
139	0		
140	0		
141	1		
142	0		

143	0
144	0
145	0
146	1

	Spinach	Root	Plant 2
	Run 1	Run 2	Run 3
Diameter (nm)	Frequency	Frequency	Frequency
36	0	0	0
37	0	0	0
38	0	0	0
39	0	0	0
40	0	0	0
41	0	0	0
42	0	0	0
43	0	0	0
44	0	0	0
45	192	173	130
46	0	0	0
47	0	0	0
48	0	0	0
49	0	0	0
50	0	0	0
51	0	0	0
52	245	208	187
53	0	0	0
54	0	0	0
55	0	0	0
56	0	0	0
57	200	191	174
58	0	0	0
59	0	0	0
60	0	0	0
61	0	0	0
62	106	96	89
63	0	0	0
64	0	0	0
65	33	35	24
66	0	0	0
67	0	0	0
68	0	0	0
69	24	11	15
70	0	0	0
71	0	0	0

72	7	7	7
73	0	0	0
74	0	0	0
75	6	4	2
76	0	0	0
77	0	0	0
78	4	4	3
79	0	0	0
80	1	4	3
81	0	0	0
82	2	2	2
83	0	0	0
84	0	0	0
85	0	4	3
86	0	0	0
87	4	1	2
88	0	0	0
89	4	1	2
90	0	0	0
91	2	1	1
92	0	0	0
93	0	1	1
94	1	1	0
95	0	0	0
96	0	0	0
97	0	0	0
98	1	0	1
99	0	3	2
100	0	0	0
101	0	2	3
102	0	0	1
103	0	0	0
104	0	2	0
105	0	0	0
106	0	0	0
107	1	0	0
108	1	3	0
109	0	0	0
110	0	0	0
111	1	1	0
112	0	1	0
113	0	1	0
114	0	0	0
115	0	0	0
116	0	0	0
117	1	0	0
118	1	0	0

119	0	0	0
120	1	0	1
121	0	0	0
122	0	0	0
123	0	1	0
124	0	0	0
125	0	0	0
126	0	0	0
127	0	0	0
128	1	0	1
129	0	0	0
130	0	0	0
131	0	0	0
132	0	0	0
133	0	0	0
134	0	0	0
135	0	0	0
136	0	0	0
137	0	0	0
138	0	0	0
139	0	0	0
140	0	0	0
141	0	0	0
142	0	0	0
143	0	0	0
144	0	0	0
145	0	0	0
146	0	0	0
147	0	0	0
148	0	0	0
149	0	0	0
150	0	0	0
151	0	0	0
152	0	0	0
153	0	0	0
154	0	0	0
155	0	1	0
156	0		0
157	0		0
158	0		0
159	0		1
160	0		1
161	0		
162	0		
163	0		
164	0		
165	0		

166	0
167	0
168	0
169	0
170	0
171	0
172	0
173	0
174	0
175	0
176	0
177	0
178	0
179	0
180	0
181	0
182	0
183	0
184	0
185	0
186	0
187	0
188	0
189	0
190	0
191	0
192	0
193	0
194	0
195	0
196	0
197	0
198	0
199	0
200	0
201	0
202	0
203	0
204	0
205	0
206	0
207	0
208	0
209	0
210	0
211	0
212	0

213	0
214	0
215	0
216	0
217	0
218	0
219	0
220	0
221	0
222	0
223	0
224	0
225	0
226	0
227	0
228	0
229	0
230	0
231	0
232	0
233	0
234	0
235	0
236	0
237	0
238	0
239	0
240	0
241	0
242	1

	Spinach	Root	Plant 3
	Run 1	Run 2	Run 3
Diameter (nm)	Frequency	Frequency	Frequency
36	0	0	0
37	0	0	0
38	0	0	0
39	0	0	0
40	0	0	0
41	0	0	0
42	0	0	0
43	0	0	0
44	0	0	0
45	34	108	109
46	0	0	0

47	0	0	0
48	0	0	0
49	0	0	0
50	0	0	0
51	0	0	0
52	49	256	219
53	0	0	0
54	0	0	0
55	0	0	0
56	0	0	0
57	65	402	431
58	0	0	0
59	0	0	0
60	0	0	0
61	0	0	0
62	119	104	93
63	0	0	0
64	0	0	0
65	27	19	32
66	0	0	0
67	0	0	0
68	0	0	0
69	9	12	17
70	0	0	0
71	0	0	0
72	7	5	6
73	0	0	0
74	0	0	0
75	1	2	3
76	0	0	0
77	0	0	0
78	1	3	1
79	0	0	0
80	0	0	0
81	0	0	0
82	0	1	0
83	0	0	0
84	0	0	0
85	0	0	0
86	0	0	0
87	0	0	1
88	0	0	0
89	1	2	0
90	0	0	0
91	1	0	0
92	0	0	0
93	0	0	0

94	0	0	0
95	0	0	0
96	1	0	0
97		0	0
98		0	0
99		0	0
100		0	0
101		0	0
102		0	0
103		0	0
104		0	1
105		0	0
106		0	0
107		0	0
108		0	0
109		0	0
110		0	0
111		0	0
112		0	0
113		0	0
114		0	0
115		0	0
116		0	0
117		1	0
118			0
119			0
120			0
121			0
122			0
123			0
124			0
125			0
126			0
127			0
128			1

	Spinach	Shoot	Plant 1
	Run 1	Run 2	Run 3
Diameter (nm)	Frequency	Frequency	Frequency
36	0	0	0
37	0	0	0
38	0	0	0
39	0	0	0
40	0	0	0
41	0	0	0

42	0	0	0
43	0	0	0
44	0	0	0
45	11	4	12
46	0	0	0
47	0	0	0
48	0	0	0
49	0	0	0
50	0	0	0
51	0	0	0
52	32	22	27
53	0	0	0
54	0	0	0
55	0	0	0
56	0	0	0
57	36	39	25
58	0	0	0
59	0	0	0
60	0	0	0
61	0	0	0
62	44	53	41
63	0	0	0
64	0	0	0
65	16	15	9
66	0	0	0
67	0	0	0
68	0	0	0
69	5	7	5
70	0	0	0
71	0	0	0
72	0	3	1
73	0	0	0
74	0	0	0
75	1	2	1
76	0	0	0
77	0	0	0
78	1	0	2
79	0	0	0
80	0	0	1
81	0	0	
82	2	1	

	Spinach	Shoot	Plant 2
	Run 1	Run 2	Run 3
Diameter (nm)	Frequency	Frequency	Frequency

36	0	0	0
37	0	0	0
38	0	0	0
39	0	0	0
40	0	0	0
41	0	0	0
42	0	0	0
43	0	0	0
44	0	0	0
45	94	93	445
46	0	0	0
47	0	0	0
48	0	0	0
49	0	0	0
50	0	0	0
51	0	0	0
52	141	131	413
53	0	0	0
54	0	0	0
55	0	0	0
56	0	0	0
57	111	100	301
58	0	0	0
59	0	0	0
60	0	0	0
61	0	0	0
62	61	60	52
63	0	0	0
64	0	0	0
65	10	12	6
66	0	0	0
67	0	0	0
68	0	0	0
69	8	6	3
70	0	0	0
71	0	0	0
72	4	5	6
73	0	0	0
74	0	0	0
75	4	1	3
76	0	0	0
77	0	0	0
78	1	2	1
79	0	0	0
80	4	0	3
81	0	0	0
82	0	0	2

83	0	0	0
84	0	0	0
85	0	1	0
86	0	0	0
87	1	0	0
88	0	0	0
89	0	0	0
90	0	0	0
91	0	1	0
92	0	0	0
93	0	0	0
94	1	1	
95	0	0	
96	0	0	
97	0	0	
98	0	0	
99	0	1	
100	0	0	
101	0	0	
102	1	0	
103	0	0	
104	0	0	
105	0	0	
106	0	0	
107	0	0	
108	0	0	
109	1	0	
110	0	0	
111	0	0	
112	0	0	
113	0	0	
114	0	0	
115	0	0	
116	0	0	
117	0	0	
118	0	0	
119	0	0	
120	0	0	
121	0	0	
122	0	0	
123	0	0	
124	0	0	
125	0	0	
126	0	0	
127	0	0	
128	0	0	
129	0	0	

130	0	0
131	0	0
132	0	0
133	0	0
134	0	0
135	0	0
136	0	0
137	0	0
138	1	0
139	0	0
140	0	0
141	0	0
142	0	0
143	0	0
144	1	0
145	0	0
146	0	0
147	0	0
148	0	0
149	0	1
150	0	0
151	0	0
152	0	0
153	0	0
154	0	1
155	0	
156	0	
157	0	
158	0	
159	0	
160	0	
161	0	
162	0	
163	1	

	Spinach	Shoot	Plant 3
Diameter (nm)	Run 1 Frequency	Run 2 Frequency	Run 3 Frequency
36	0	0	0
37	0	0	0
38	0	0	0
39	0	0	0
40	0	0	0
41	0	0	0
42	0	0	0

43	0	0	0
44	0	0	0
45	4	19	10
46	0	0	0
47	0	0	0
48	0	0	0
49	0	0	0
50	0	0	0
51	0	0	0
52	18	58	17
53	0	0	0
54	0	0	0
55	0	0	0
56	0	0	0
57	29	51	16
58	0	0	0
59	0	0	0
60	0	0	0
61	0	0	0
62	11	95	22
63	0	0	0
64	0	0	0
65	29	29	16
66	0	0	0
67	0	0	0
68	0	0	0
69	3	14	9
70	0	0	0
71	0	0	0
72	5	3	2
73	0	0	0
74	0	0	0
75	1	3	3
76	0	0	0
77	0	0	0
78	1	0	1
79	0	0	0
80	1	0	2
81	0	0	0
82	1	1	1
83	0	0	0
84	0	0	0
85	2	1	0
86	0	0	0
87	1	1	1
88	0	0	0
89	2	0	1

90	0	0	0
91	0	0	0
92	0	0	0
93	0	1	0
94	0	0	0
95	0	0	0
96	0	0	0
97	0	0	0
98	0	0	0
99	0	0	0
100	0	0	0
101	0	0	0
102	0	0	0
103	0	0	0
104	1	0	0
105	0	0	0
106	0	0	0
107	0	0	0
108	1	0	0
109	0	0	0
110	0	0	0
111	0	0	0
112	0	0	0
113	0	0	0
114	0	1	0
115	0	0	0
116	0	0	0
117	0	0	0
118	0	0	0
119	0	0	0
120	0	1	0
121	0		0
122	0		0
123	0		0
124	0		0
125	0		0
126	0		0
127	0		0
128	0		0
129	0		0
130	0		0
131	0		1
132	0		
133	0		
134	0		
135	1		

	Brussels Sprout	Root	Plant 1
	Run 1	Run 2	Run 3
Diameter (nm)	Frequency	Frequency	Frequency
34	0	0	0
35	0	0	0
36	0	0	0
37	0	0	0
38	0	0	0
39	0	0	0
40	0	0	0
41	0	0	0
42	0	0	0
43	2	2	5
44	0	0	0
45	0	0	0
46	0	0	0
47	0	0	0
48	0	0	0
49	41	50	47
50	0	0	0
51	0	0	0
52	0	0	0
53	0	0	0
54	60	78	61
55	0	0	0
56	0	0	0
57	0	0	0
58	69	69	76
59	0	0	0
60	0	0	0
61	93	78	103
62	0	0	0
63	0	0	0
64	0	0	0
65	86	97	120
66	0	0	0
67	0	0	0
68	83	98	75
69	0	0	0
70	88	87	83
71	0	0	0
72	0	0	0
73	63	78	56
74	0	0	0
75	70	63	53

76	0	0	0
77	60	48	56
78	0	0	0
79	52	50	60
80	0	0	0
81	60	52	52
82	0	0	0
83	57	37	43
84	0	0	0
85	38	55	42
86	0	0	0
87	35	37	51
88	0	0	0
89	42	39	26
90	27	40	28
91	0	0	0
92	25	36	34
93	31	35	38
94	0	0	0
95	23	33	25
96	21	31	36
97	22	21	34
98	0	0	0
99	21	33	19
100	16	24	39
101	28	21	29
102	0	0	0
103	21	20	24
104	13	27	26
105	16	22	24
106	23	17	20
107	17	12	21
108	25	23	15
109	17	18	16
110	0	0	0
111	13	25	16
112	18	12	26
113	14	19	24
114	13	16	17
115	15	20	13
116	35	28	32
117	13	18	14
118	14	18	9
119	13	21	13
120	12	14	11
121	11	12	8
122	9	11	7

123	16	9	11
124	21	25	21
125	12	11	11
126	17	14	11
127	11	11	9
128	10	12	17
129	21	8	8
130	7	13	10
131	7	8	7
132	16	16	23
133	8	13	12
134	13	20	24
135	9	12	5
136	7	10	6
137	21	10	17
138	11	5	6
139	9	18	14
140	13	4	5
141	14	11	17
142	8	8	15
143	2	7	10
144	16	20	14
145	5	6	10
146	12	6	13
147	19	12	22
148	5	4	9
149	9	10	12
150	10	12	21
151	13	13	9
152	8	7	5
153	15	7	17
154	8	8	9
155	13	7	11
156	6	10	12
157	13	7	11
158	8	14	6
159	7	4	13
160	11	8	8
161	13	4	7
162	11	14	5
163	9	6	4
164	7	13	10
165	11	12	13
166	10	11	9
167	4	5	3
168	15	9	9
169	7	4	5

170	5	7	8
171	9	6	6
172	12	10	11
173	5	17	2
174	5	2	6
175	7	12	11
176	8	5	5
177	15	6	16
178	9	10	11
179	4	2	2
180	16	8	13
181	7	7	8
182	7	5	7
183	8	10	6
184	7	4	7
185	10	6	7
186	10	7	10
187	8	7	4
188	4	10	7
189	7	5	5
190	9	8	6
191	6	7	5
192	3	4	2
193	8	10	6
194	5	2	9
195	0	6	7
196	6	6	2
197	5	4	7
198	4	10	7
199	2	4	11
200	8	12	9
201	5	4	5
202	4	4	5
203	4	7	6
204	3	6	9
205	5	10	6
206	7	9	1
207	5	6	7
208	4	1	6
209	3	8	7
210	5	4	3
211	4	4	8
212	6	4	2
213	6	4	4
214	10	3	5
215	5	2	2
216	2	4	4

217	4	4	3
218	5	6	4
219	2	4	5
220	5	5	3
221	2	7	0
222	4	3	5
223	3	1	7
224	5	4	4
225	5	7	6
226	5	5	4
227	4	4	3
228	4	2	2
229	3	5	4
230	8	3	3
231	5	5	3
232	4	3	5
233	5	3	6
234	3	0	10
235	3	0	1
236	3	1	4
237	5	2	2
238	4	2	4
239	1	3	3
240	3	1	3
241	4	3	1
242	2	6	2
243	4	2	0
244	1	3	7
245	4	4	1
246	1	0	6
247	2	4	5
248	0	2	2
249	7	2	4
250	4	3	4
251	1	3	1
252	0	3	2
253	3	2	1
254	2	4	2
255	1	5	2
256	3	2	1
257	2	3	2
258	3	2	1
259	1	2	2
260	0	3	1
261	2	6	2
262	2	2	2
263	2	2	1

264	2	6	2
265	2	1	0
266	0	3	1
267	2	2	3
268	2	0	3
269	2	0	0
270	2	1	1
271	0	4	1
272	2	1	1
273	1	0	1
274	1	4	3
275	1	0	0
276	1	1	1
277	1	1	0
278	2	1	1
279	0	2	0
280	0	2	2
281	0	0	0
282	0	0	1
283	1	1	1
284	3	3	1
285	0	2	1
286	2	1	0
287	1	2	0
288	1	1	2
289	0	2	0
290	1	0	1
291	0	0	2
292	0	4	1
293	0	0	2
294	0	1	0
295	1	2	0
296	2	2	0
297	0	0	0
298	1	1	0
299	1	1	0
300	1	2	1
301	0	0	0
302	3	0	1
303	2	1	1
304	0	1	2
305	1	0	0
306	1	0	0
307	1	1	1
308	1	1	2
309	0	2	0
310	0	1	0

311	0	0	2
312	1	0	0
313	0	1	0
314	0	1	1
315	0	0	0
316	0	0	0
317	0	0	0
318	0	0	1
319	2	0	0
320	1	0	0
321	2	0	1
322	0	0	0
323	1	0	0
324	0	0	1
325	0	0	2
326	1	1	2
327	1	1	0
328	0	0	0
329	0	0	1
330	0	0	1
331	1	0	0
332	0	2	0
333	0	0	1
334	0	1	0
335	1	1	1
336	0	0	0
337	1	1	1
338	1	1	0
339	0	0	1
340	0	1	0
341	0	1	0
342	1	0	0
343	0	0	2
344	0	0	0
345	2	0	0
346	0	0	0
347	0	0	0
348	0	0	0
349	0	1	0
350	0	0	0
351	0	0	0
352	0	0	0
353	0	0	0
354	1	0	1
355	0	1	0
356	0	0	2
357	0	0	0

358	0	1	0
359	0	0	0
360	1	0	0
361	0	1	0
362	0	0	0
363	1	0	0
364	0	0	0
365	0	0	0
366	0	1	0
367	0	0	0
368	0	0	2
369	2	0	0
370	0	0	0
371	0	2	0
372	1	1	0
373	0	0	0
374	1	0	0
375	1	0	0
376	0	0	0
377	0	0	0
378	0	0	0
379	0	0	1
380	0	1	0
381	0	0	0
382	1	0	0
383	2	0	0
384	0	0	0
385	0	0	0
386	1	0	2
387	0	1	0
388	1	0	0
389	0	0	0
390	0	0	0
391	0	0	0
392	0	0	0
393	0	0	0
394	0	0	0
395	0	0	0
396	0	1	1
397	0	0	1
398	0	0	0
399	0	0	0
400	0	0	1
401	0	0	0
402	0	0	0
403	0	0	0
404	0	0	0

405	0	0	0
406	0	0	0
407	0	0	0
408	0	0	0
409	0	0	0
410	0	0	0
411	0	0	0
412	0	0	0
413	0	0	0
414	0	1	0
415	0	0	0
416	0	0	0
417	0	0	0
418	0	0	0
419	0	0	0
420	0	0	0
421	0	0	0
422	0	0	0
423	0	0	0
424	0	0	0
425	0	0	0
426	0	0	0
427	0	0	0
428	0	0	0
429	0	0	0
430	0	0	0
431	0	0	0
432	0	0	0
433	0	0	0
434	0	0	1
435	0	0	0
436	0	0	1
437	0	0	0
438	0	0	0
439	0	0	0
440	0	0	0
441	0	0	1
442	0	0	0
443	0	0	0
444	0	1	1
445	0		0
446	0		0
447	0		0
448	0		0
449	0		0
450	0		0
451	0		0

452	0		0
453	0		0
454	0		0
455	0		0
456	0		0
457	0		0
458	0		0
459	0		0
460	1		0
461	0		0
462	0		0
463	0		0
464	0		0
465	0		0
466	0		0
467	0		0
468	0		0
469	0		0
470	0		0
471	0		0
472	0		0
473	0		0
474	0		0
475	0		0
476	0		0
477	0		0
478	0		0
479	1		0
480			0
481			1

	Brussels Sprout	Root	Plant 2
	Run 1	Run 2	Run 3
Diameter (nm)	Frequency	Frequency	Frequency
34	0	0	0
35	0	0	0
36	0	0	0
37	0	0	0
38	0	0	0
39	0	0	0
40	0	0	0
41	0	0	0
42	0	0	0
43	13	27	39
44	0	0	0

45	0	0	0
46	0	0	0
47	0	0	0
48	0	0	0
49	68	80	67
50	0	0	0
51	0	0	0
52	0	0	0
53	0	0	0
54	56	72	57
55	0	0	0
56	0	0	0
57	0	0	0
58	62	85	69
59	0	0	0
60	0	0	0
61	45	69	58
62	0	0	0
63	0	0	0
64	0	0	0
65	48	68	77
66	0	0	0
67	0	0	0
68	34	53	61
69	0	0	0
70	41	43	48
71	0	0	0
72	0	0	0
73	26	47	30
74	0	0	0
75	36	36	29
76	0	0	0
77	25	14	23
78	0	0	0
79	27	21	21
80	0	0	0
81	18	21	20
82	0	0	0
83	16	17	21
84	0	0	0
85	11	19	13
86	0	0	0
87	17	11	10
88	0	0	0
89	16	9	18
90	10	13	19
91	0	0	0

92	8	7	16
93	7	11	10
94	0	0	0
95	11	11	11
96	13	18	9
97	6	4	6
98	0	0	0
99	15	4	10
100	8	10	15
101	4	10	9
102	0	0	0
103	11	7	8
104	9	10	6
105	8	5	8
106	4	13	8
107	5	4	6
108	4	4	6
109	4	10	9
110	0	0	0
111	11	5	4
112	3	12	6
113	3	5	8
114	4	3	4
115	8	10	7
116	7	11	9
117	8	3	2
118	3	4	1
119	1	4	10
120	5	7	5
121	4	5	6
122	2	7	7
123	4	6	4
124	12	9	10
125	4	3	2
126	8	2	6
127	3	4	2
128	6	7	8
129	5	4	3
130	4	4	2
131	5	4	6
132	4	8	9
133	14	2	1
134	8	10	8
135	6	3	5
136	7	6	0
137	13	7	4
138	4	1	3

139	10	7	5
140	6	5	3
141	6	6	5
142	8	9	9
143	3	1	4
144	10	4	4
145	3	3	3
146	10	6	7
147	8	6	9
148	4	2	3
149	3	8	4
150	7	7	7
151	8	9	8
152	2	1	1
153	3	16	8
154	9	2	5
155	4	1	6
156	5	3	5
157	3	4	5
158	4	5	7
159	6	5	4
160	6	2	3
161	4	4	4
162	3	2	4
163	3	5	5
164	5	2	4
165	4	7	4
166	2	5	1
167	4	2	1
168	7	8	7
169	5	0	0
170	6	1	4
171	6	0	0
172	7	7	6
173	4	8	2
174	4	5	2
175	1	8	3
176	5	0	2
177	1	4	3
178	1	2	3
179	3	1	2
180	4	2	3
181	4	3	2
182	6	5	3
183	1	8	6
184	1	5	0
185	2	9	3

186	3	8	5
187	3	2	1
188	0	9	4
189	2	5	6
190	6	3	3
191	3	4	4
192	3	2	0
193	4	3	0
194	3	5	3
195	1	4	1
196	3	2	5
197	7	2	6
198	2	3	0
199	1	2	1
200	4	2	2
201	2	3	1
202	2	2	4
203	1	2	1
204	2	4	1
205	1	3	1
206	1	1	0
207	3	0	0
208	1	5	1
209	2	1	1
210	0	2	0
211	3	3	1
212	1	0	2
213	3	4	1
214	4	3	1
215	2	6	3
216	0	1	2
217	5	1	0
218	2	3	2
219	3	2	3
220	1	0	1
221	0	1	0
222	2	1	1
223	1	3	0
224	1	2	1
225	1	2	0
226	0	0	0
227	0	1	1
228	2	0	2
229	3	6	3
230	1	3	1
231	0	3	0
232	1	1	1

233	3	2	2
234	1	0	2
235	0	3	3
236	3	0	0
237	1	0	0
238	1	2	2
239	2	1	0
240	0	1	3
241	2	0	2
242	1	1	0
243	1	5	0
244	1	0	1
245	0	0	2
246	0	0	2
247	2	0	1
248	1	2	1
249	1	2	1
250	1	0	0
251	2	1	0
252	1	3	2
253	1	1	3
254	2	1	2
255	1	1	1
256	4	0	1
257	0	1	2
258	0	0	1
259	0	0	1
260	1	0	0
261	1	0	1
262	2	0	0
263	2	1	2
264	2	1	3
265	2	0	0
266	0	0	0
267	1	1	1
268	2	0	1
269	0	1	1
270	1	1	0
271	0	0	2
272	1	1	0
273	0	1	1
274	2	0	1
275	0	1	1
276	0	0	1
277	2	0	0
278	2	0	2
279	0	1	0

280	1	1	1
281	2	0	0
282	1	0	0
283	0	0	0
284	1	0	0
285	1	1	0
286	0	0	0
287	1	0	1
288	1	0	0
289	1	0	0
290	2	1	0
291	0	0	0
292	0	3	0
293	0	0	0
294	0	0	1
295	1	1	0
296	0	1	0
297	0	0	1
298	1	0	0
299	1	0	1
300	0	2	0
301	1	0	0
302	1	1	1
303	1	1	1
304	0	1	0
305	1	0	1
306	0	0	0
307	0	1	1
308	2	0	0
309	0	0	3
310	0	1	0
311	0	1	0
312	1	0	1
313	0	0	0
314	0	0	0
315	0	0	0
316	0	0	0
317	0	0	0
318	0	0	0
319	1	1	0
320	1	0	0
321	0	0	1
322	0	0	1
323	0	1	0
324	0	0	0
325	0	0	0
326	0	0	0

327	0	1	0
328	0	1	0
329	0	0	0
330	1	0	1
331	0	0	0
332	1	0	0
333	0	0	0
334	0	2	0
335	0	0	0
336	1	0	0
337	0	0	0
338	0	0	0
339	1	0	0
340	0	1	0
341	0	0	0
342	0	0	0
343	0	0	0
344	0	0	0
345	0	0	0
346	0	0	0
347	0	0	1
348	0	0	0
349	0	0	0
350	1	0	0
351	0	0	0
352	0	0	0
353	0	0	0
354	0	0	1
355	0	1	0
356	0	0	0
357	0	0	0
358	0	1	0
359	0	0	0
360	0	0	0
361	0	0	0
362	1	0	0
363	0	0	0
364	0	0	0
365	0	0	0
366	0	0	0
367	0	0	0
368	0	0	0
369	0	0	0
370	0	0	0
371	0	0	0
372	0	0	0
373	0	1	0

374	0	0	0
375	0	0	0
376	0	0	0
377	0	0	0
378	0	0	0
379	0	0	0
380	0	0	0
381	0	0	0
382	0	0	0
383	0	0	1
384	1	0	0
385		0	0
386		0	0
387		0	0
388		0	0
389		1	0
390			0
391			0
392			0
393			0
394			0
395			0
396			0
397			0
398			0
399			0
400			0
401			0
402			0
403			0
404			0
405			0
406			0
407			0
408			0
409			0
410			0
411			0
412			0
413			0
414			1
415			0
416			0
417			0
418			0
419			0
420			0

421			1
422			1

	Brussels Sprout	Root	Plant 3
	Run 1	Run 2	Run 3
Diameter (nm)	Frequency	Frequency	Frequency
34	0	0	0
35	0	0	0
36	0	0	0
37	0	0	0
38	0	0	0
39	0	0	0
40	0	0	0
41	0	0	0
42	0	0	0
43	15	23	11
44	0	0	0
45	0	0	0
46	0	0	0
47	0	0	0
48	0	0	0
49	73	81	63
50	0	0	0
51	0	0	0
52	0	0	0
53	0	0	0
54	66	71	65
55	0	0	0
56	0	0	0
57	0	0	0
58	84	75	64
59	0	0	0
60	0	0	0
61	63	71	80
62	0	0	0
63	0	0	0
64	0	0	0
65	76	45	65
66	0	0	0
67	0	0	0
68	69	67	60
69	0	0	0
70	56	43	54
71	0	0	0
72	0	0	0

73	38	34	32
74	0	0	0
75	32	29	28
76	0	0	0
77	20	11	20
78	0	0	0
79	19	14	17
80	0	0	0
81	17	17	11
82	0	0	0
83	12	16	8
84	0	0	0
85	11	9	13
86	0	0	0
87	6	6	7
88	0	0	0
89	10	7	7
90	7	8	5
91	0	0	0
92	6	6	10
93	5	8	11
94	0	0	0
95	9	5	5
96	7	4	4
97	6	6	3
98	0	0	0
99	5	9	3
100	5	10	4
101	6	5	7
102	0	0	0
103	4	3	1
104	2	2	7
105	4	6	1
106	5	8	6
107	6	3	7
108	5	2	5
109	3	6	4
110	0	0	0
111	1	4	3
112	6	5	1
113	6	6	5
114	4	6	2
115	5	0	0
116	10	7	9
117	5	1	4
118	8	8	3
119	6	4	6

120	4	3	5
121	6	5	5
122	5	3	3
123	0	2	3
124	7	3	8
125	5	3	1
126	3	3	0
127	5	3	1
128	5	5	1
129	1	2	2
130	5	1	2
131	2	2	1
132	6	4	3
133	4	1	1
134	6	6	7
135	2	1	4
136	3	1	2
137	7	7	4
138	0	1	2
139	5	5	5
140	0	3	2
141	6	2	4
142	4	3	3
143	0	1	4
144	6	5	4
145	2	3	5
146	5	3	4
147	1	4	6
148	1	0	3
149	1	4	5
150	6	2	3
151	1	4	2
152	1	1	2
153	7	5	7
154	6	2	7
155	4	2	3
156	4	4	5
157	1	3	2
158	2	5	1
159	2	6	3
160	1	2	2
161	2	1	5
162	4	4	4
163	4	1	1
164	6	2	0
165	1	4	2
166	1	3	3

167	0	2	2
168	7	2	1
169	2	2	2
170	3	2	4
171	2	5	0
172	4	4	1
173	0	2	1
174	1	3	2
175	2	4	5
176	2	0	3
177	5	2	1
178	3	0	0
179	1	4	1
180	4	1	6
181	2	0	5
182	2	1	3
183	4	4	7
184	2	3	3
185	3	2	2
186	5	1	3
187	2	2	3
188	2	5	3
189	4	6	1
190	0	3	2
191	3	2	2
192	1	4	3
193	2	3	2
194	1	2	3
195	4	3	2
196	2	1	1
197	5	1	0
198	2	2	3
199	3	2	3
200	4	3	1
201	1	0	0
202	5	5	3
203	2	2	0
204	4	0	1
205	2	1	5
206	6	2	2
207	2	1	1
208	2	0	4
209	2	1	4
210	1	1	1
211	8	2	2
212	1	1	0
213	0	2	1

214	0	0	1
215	3	1	3
216	1	2	3
217	3	1	3
218	2	3	1
219	0	0	2
220	1	1	1
221	0	1	2
222	0	0	3
223	2	1	1
224	3	1	0
225	2	1	2
226	1	2	2
227	0	3	3
228	1	1	0
229	3	2	1
230	2	2	2
231	2	1	1
232	1	2	1
233	3	4	0
234	1	0	0
235	1	0	0
236	1	1	1
237	1	0	2
238	1	1	0
239	1	1	2
240	2	2	1
241	1	2	0
242	1	0	3
243	1	1	0
244	0	0	0
245	0	0	0
246	1	1	0
247	0	1	0
248	0	1	1
249	3	3	0
250	0	0	0
251	2	1	2
252	0	1	1
253	0	0	1
254	0	1	0
255	2	0	1
256	0	1	2
257	1	1	0
258	1	0	2
259	3	1	0
260	1	0	2

261	1	2	0
262	2	0	2
263	1	1	2
264	0	0	1
265	0	2	0
266	0	0	0
267	2	0	1
268	2	1	1
269	0	0	0
270	0	0	0
271	0	2	0
272	2	3	1
273	1	1	1
274	1	1	1
275	2	0	0
276	1	3	0
277	0	0	1
278	0	0	1
279	0	1	2
280	1	1	0
281	0	1	1
282	1	0	0
283	0	1	0
284	0	0	0
285	0	0	0
286	1	0	2
287	3	1	0
288	0	0	1
289	0	0	1
290	0	0	0
291	0	0	0
292	0	1	1
293	1	0	2
294	0	0	0
295	0	1	1
296	1	0	0
297	1	0	0
298	1	1	0
299	1	0	0
300	0	0	0
301	1	0	0
302	0	0	0
303	1	0	0
304	0	0	1
305	0	0	1
306	1	0	0
307	2	0	0

308	0	0	2
309	0	2	0
310	0	1	0
311	0	0	0
312	0	0	1
313	0	1	0
314	0	0	0
315	0	2	0
316	0	0	0
317	0	0	0
318	0	0	0
319	0	0	0
320	0	1	0
321	0	1	0
322	0	0	0
323	0	0	1
324	0	0	0
325	1	0	0
326	0	0	0
327	1	0	0
328	0	0	0
329	1	0	0
330	0	0	1
331	0	2	0
332	0	1	0
333	2	0	0
334	0	0	0
335	0	0	0
336	0	0	0
337	0	0	0
338	0	0	0
339	0	0	0
340	0	0	0
341	0	0	0
342	1	0	0
343	0	0	0
344	1	1	0
345	0	0	0
346	0	0	0
347	0	0	0
348	1	0	0
349	0	0	0
350	0	0	0
351	0	1	0
352	1	0	0
353	0	0	0
354	0	0	1

355	0	0	0
356	0	0	1
357	0	0	0
358	0	0	0
359	0	0	0
360	0	0	0
361	0	0	0
362	0	0	0
363	0	0	0
364	0	0	0
365	0	0	0
366	0	0	0
367	0	0	0
368	0	0	0
369	0	0	0
370	0	0	0
371	0	1	0
372	0	0	1
373	0	1	0
374	0	0	0
375	0	0	0
376	0	0	0
377	0	0	0
378	0	0	0
379	0	1	0
380	0	0	0
381	0	0	0
382	0	0	0
383	0	0	0
384	0	0	0
385	0	0	0
386	0	0	0
387	0	0	0
388	0	0	0
389	0	0	0
390	0	0	0
391	0	0	0
392	0	0	0
393	0	0	0
394	0	0	0
395	0	0	0
396	0	0	0
397	1	0	1
398		0	0
399		0	0
400		0	0
401		0	0

402		0	0
403		0	1
404		0	1
405		0	
406		0	
407		0	
408		0	
409		0	
410		0	
411		0	
412		0	
413		0	
414		1	

	Brussels Sprout	Shoot	Plant 1
	Run 1	Run 2	Run 3
Diameter (nm)	Frequency	Frequency	Frequency
34	1	0	0
35	0	0	0
36	0	0	0
37	0	0	0
38	0	0	0
39	0	0	0
40	0	0	0
41	0	0	0
42	0	0	0
43	41	3	5
44	0	0	0
45	0	0	0
46	0	0	0
47	0	0	0
48	0	0	0
49	65	2	7
50	0	0	0
51	0	0	0
52	0	0	0
53	0	0	0
54	66	3	11
55	0	0	0
56	0	0	0
57	0	0	0
58	11	0	10
59	0	0	0
60	0	0	0
61	2	5	1

62	0	0	0
63	0	0	0
64	0	0	0
65	2	1	0
66	0	0	0
67	0	0	0
68	1	0	0
69	0	0	0
70	1	0	1
71	0	0	0
72	0	0	0
73	0	2	0
74	0	0	0
75	1	0	0
76	0	0	0
77	0	0	1
78	0	0	0
79	0	1	0
80	0	0	0
81	1	1	0
82	0	0	0
83	0	0	0
84	0	0	0
85	0	0	0
86	0	0	0
87	0	0	0
88	0	0	0
89	0	0	0
90	0	0	0
91	0	0	0
92	0	0	0
93	0	0	0
94	0	0	0
95	0	0	0
96	0	0	1
97	0	0	0
98	0	0	0
99	0	0	0
100	0	1	0
101	0	0	1
102	0	0	0
103	0	0	0
104	0	0	0
105	0	0	0
106	0	0	0
107	0	0	0
108	0	0	0

109	0	0	0
110	0	0	0
111	1	0	0
112	0	0	0
113	0	0	0
114	0	0	1
115	0	0	0
116	0	0	0
117	0	0	0
118	0	0	0
119	0	0	0
120	0	0	0
121	0	0	0
122	0	0	0
123	0	0	0
124	0	0	0
125	0	0	1
126	0	0	0
127	0	0	0
128	0	0	0
129	0	0	0
130	0	0	0
131	0	0	0
132	0	0	0
133	0	0	0
134	1	0	0
135		0	0
136		0	0
137		0	0
138		0	0
139		0	0
140		0	0
141		1	0
142		0	0
143		0	0
144		0	0
145		0	0
146		0	0
147		0	0
148		0	0
149		0	0
150		0	0
151		0	0
152		0	0
153		0	0
154		0	0
155		0	0

156	0	0
157	0	0
158	0	0
159	0	1
160	0	
161	0	
162	0	
163	0	
164	0	
165	0	
166	0	
167	1	
168	0	
169	0	
170	0	
171	0	
172	0	
173	0	
174	0	
175	1	
176	0	
177	0	
178	0	
179	0	
180	0	
181	0	
182	0	
183	0	
184	0	
185	0	
186	0	
187	0	
188	0	
189	0	
190	0	
191	0	
192	0	
193	0	
194	0	
195	0	
196	0	
197	0	
198	0	
199	0	
200	0	
201	0	
202	0	

203	0
204	0
205	0
206	0
207	0
208	0
209	0
210	0
211	0
212	0
213	0
214	0
215	0
216	0
217	0
218	0
219	0
220	0
221	0
222	0
223	0
224	0
225	0
226	0
227	0
228	0
229	0
230	0
231	0
232	0
233	0
234	0
235	0
236	0
237	0
238	0
239	1

	Brussels Sprout	Shoot	Plant 2
	Run 1	Run 2	Run 3
Diameter (nm)	Frequency	Frequency	Frequency
34	786	4	24
35	0	0	0
36	0	0	0
37	0	0	0

38	0	0	0
39	0	0	0
40	0	0	0
41	0	0	0
42	0	0	0
43	4310	24	163
44	0	0	0
45	0	0	0
46	0	0	0
47	0	0	0
48	0	0	0
49	411	34	328
50	0	0	0
51	0	0	0
52	0	0	0
53	0	0	0
54	35	24	37
55	0	0	0
56	0	0	0
57	0	0	0
58	7	4	4
59	0	0	0
60	0	0	0
61	0	3	1
62	0	0	0
63	0	0	0
64	0	0	0
65	0	0	0
66	0	0	0
67	0	0	0
68	2	0	0
69	0	0	0
70	1	0	0
71	0	0	0
72	0	0	0
73	0	0	0
74	0	0	0
75	0	0	0
76	0	0	0
77	0	0	0
78	0	0	0
79	1	0	0
80		0	0
81		0	0
82		0	0
83		0	0
84		0	0

85	0	0
86	0	0
87	0	1
88	0	0
89	0	0
90	0	0
91	0	0
92	0	1
93	0	0
94	0	0
95	0	0
96	0	0
97	0	0
98	0	0
99	0	0
100	0	0
101	0	0
102	0	0
103	0	0
104	0	0
105	0	0
106	0	0
107	0	0
108	0	0
109	0	0
110	0	0
111	0	0
112	0	0
113	0	0
114	0	0
115	0	0
116	0	0
117	0	0
118	0	0
119	0	0
120	0	0
121	0	0
122	0	0
123	0	0
124	0	0
125	0	0
126	0	0
127	0	0
128	0	0
129	0	0
130	0	0
131	0	0

132	0	0
133	0	0
134	0	0
135	0	0
136	0	0
137	0	0
138	0	0
139	0	0
140	0	0
141	0	0
142	0	0
143	0	0
144	0	1
145	0	
146	0	
147	0	
148	0	
149	0	
150	0	
151	0	
152	0	
153	0	
154	0	
155	0	
156	0	
157	0	
158	0	
159	0	
160	0	
161	0	
162	0	
163	0	
164	0	
165	0	
166	0	
167	0	
168	0	
169	0	
170	0	
171	0	
172	0	
173	0	
174	0	
175	0	
176	0	
177	0	
178	0	

179	0
180	0
181	0
182	0
183	0
184	0
185	0
186	0
187	0
188	0
189	0
190	0
191	0
192	0
193	0
194	0
195	0
196	0
197	0
198	0
199	0
200	0
201	0
202	0
203	0
204	0
205	0
206	0
207	0
208	0
209	0
210	0
211	0
212	0
213	0
214	0
215	0
216	1
217	0
218	0
219	0
220	0
221	0
222	0
223	0
224	0
225	0

226	0
227	0
228	0
229	0
230	0
231	0
232	0
233	0
234	0
235	0
236	0
237	0
238	1

	Brussels Sprout	Shoot	Plant 3
	Run 1	Run 2	Run 3
Diameter (nm)	Frequency	Frequency	Frequency
34	0	1	0
35	0	0	0
36	0	0	0
37	0	0	0
38	0	0	0
39	0	0	0
40	0	0	0
41	0	0	0
42	0	0	0
43	13	21	45
44	0	0	0
45	0	0	0
46	0	0	0
47	0	0	0
48	0	0	0
49	13	28	96
50	0	0	0
51	0	0	0
52	0	0	0
53	0	0	0
54	28	42	171
55	0	0	0
56	0	0	0
57	0	0	0
58	32	40	37
59	0	0	0
60	0	0	0
61	15	7	16

62	0	0	0
63	0	0	0
64	0	0	0
65	3	4	2
66	0	0	0
67	0	0	0
68	0	0	2
69	0	0	0
70	1	2	1
71	0	0	
72	0	0	
73	2	1	
74	0	0	
75	0	1	
76	0	0	
77	0	2	
78	0	0	
79	0	1	
80	0	0	
81	0	2	
82	0	0	
83	0	0	
84	0	0	
85	0	0	
86	0	0	
87	0	0	
88	0	0	
89	0	0	
90	0	0	
91	0	0	
92	0	0	
93	0	0	
94	0	0	
95	0	0	
96	0	0	
97	0	1	
98	0		
99	0		
100	0		
101	0		
102	0		
103	0		
104	0		
105	0		
106	0		
107	0		
108	0		

109	0
110	0
111	0
112	0
113	0
114	0
115	0
116	0
117	0
118	0
119	0
120	0
121	0
122	0
123	0
124	0
125	0
126	0
127	0
128	0
129	0
130	0
131	0
132	0
133	0
134	0
135	0
136	0
137	0
138	0
139	0
140	0
141	0
142	0
143	0
144	0
145	0
146	0
147	0
148	0
149	0
150	0
151	0
152	0
153	0
154	0
155	0

156	0
157	0
158	0
159	0
160	0
161	0
162	0
163	0
164	0
165	0
166	0
167	0
168	0
169	0
170	0
171	0
172	0
173	0
174	0
175	0
176	0
177	0
178	0
179	1

	Broccoli	Root	Plant 1
	Run 1	Run 2	Run 3
Diameter (nm)	Frequency	Frequency	Frequency
34	0	0	0
35	0	0	0
36	0	0	0
37	0	0	0
38	0	0	0
39	0	0	0
40	0	0	0
41	0	0	0
42	0	0	0
43	14	8	6
44	0	0	0
45	0	0	0
46	0	0	0
47	0	0	0
48	0	0	0
49	47	36	37
50	0	0	0

51	0	0	0
52	0	0	0
53	0	0	0
54	48	46	69
55	0	0	0
56	0	0	0
57	0	0	0
58	52	62	64
59	0	0	0
60	0	0	0
61	61	88	66
62	0	0	0
63	0	0	0
64	0	0	0
65	94	81	77
66	0	0	0
67	0	0	0
68	88	97	91
69	0	0	0
70	115	117	112
71	0	0	0
72	0	0	0
73	157	128	142
74	0	0	0
75	131	120	128
76	0	0	0
77	123	94	116
78	0	0	0
79	96	120	96
80	0	0	0
81	99	112	114
82	0	0	0
83	86	82	81
84	0	0	0
85	85	76	64
86	0	0	0
87	64	82	81
88	0	0	0
89	71	69	80
90	71	77	63
91	0	0	0
92	48	65	63
93	58	57	46
94	0	0	0
95	39	56	70
96	59	63	66
97	39	51	64

98	0	0	0
99	51	45	46
100	51	48	58
101	48	48	47
102	0	0	0
103	36	45	37
104	47	36	44
105	43	55	34
106	51	40	31
107	44	43	37
108	31	39	33
109	32	47	49
110	0	0	0
111	36	53	34
112	33	28	37
113	24	29	22
114	31	34	36
115	24	29	21
116	50	53	48
117	35	28	25
118	27	17	26
119	19	25	25
120	23	25	27
121	20	30	29
122	31	27	24
123	24	27	14
124	44	38	50
125	27	20	23
126	21	23	24
127	21	17	21
128	41	46	35
129	17	15	17
130	13	15	14
131	28	20	26
132	37	38	37
133	9	18	11
134	36	31	36
135	14	24	12
136	18	19	11
137	34	51	28
138	15	17	15
139	32	35	39
140	12	19	14
141	27	22	31
142	26	27	37
143	8	11	16
144	20	32	27

145	19	8	13
146	23	21	26
147	27	22	24
148	11	18	12
149	27	27	20
150	19	26	19
151	17	28	22
152	7	16	19
153	18	14	28
154	24	22	20
155	16	17	12
156	18	21	17
157	20	17	24
158	13	25	26
159	11	13	23
160	23	20	10
161	22	12	11
162	13	16	18
163	10	18	20
164	11	12	19
165	19	16	23
166	12	14	22
167	19	11	17
168	21	21	16
169	15	16	19
170	10	13	6
171	12	12	14
172	20	26	25
173	10	12	14
174	7	11	12
175	17	11	23
176	13	7	8
177	13	22	18
178	8	9	10
179	11	9	14
180	17	14	11
181	15	18	12
182	9	10	9
183	9	14	9
184	6	9	15
185	11	13	9
186	17	12	21
187	8	11	8
188	16	18	12
189	5	13	8
190	11	12	9
191	13	9	10

192	7	8	7
193	10	7	5
194	6	14	12
195	10	14	13
196	7	14	7
197	13	7	11
198	8	9	2
199	7	6	10
200	10	14	12
201	4	11	12
202	8	4	3
203	6	13	7
204	3	13	9
205	13	12	11
206	12	3	9
207	7	5	5
208	7	9	11
209	1	6	7
210	7	8	6
211	4	10	5
212	4	3	12
213	7	12	4
214	4	7	8
215	9	8	9
216	5	12	9
217	1	4	4
218	6	14	3
219	5	12	4
220	4	9	8
221	1	9	7
222	7	3	8
223	3	5	12
224	5	4	9
225	8	8	9
226	4	4	3
227	1	5	3
228	4	4	3
229	2	2	4
230	3	6	3
231	4	3	2
232	3	3	4
233	5	2	9
234	4	7	8
235	3	2	4
236	2	8	2
237	2	4	3
238	0	10	0

239	4	6	8
240	3	2	2
241	5	4	2
242	1	1	4
243	4	1	4
244	3	4	3
245	5	4	2
246	3	3	4
247	0	1	2
248	4	3	3
249	4	4	2
250	2	2	2
251	1	1	7
252	5	3	6
253	2	2	3
254	2	0	2
255	2	5	4
256	1	0	1
257	1	4	1
258	1	1	3
259	3	5	6
260	6	0	3
261	3	2	1
262	4	1	1
263	2	0	0
264	1	0	1
265	1	5	5
266	0	1	1
267	2	1	1
268	1	0	3
269	1	0	2
270	1	1	3
271	3	2	4
272	1	2	1
273	2	2	2
274	2	0	3
275	1	4	0
276	1	0	1
277	3	0	1
278	0	0	1
279	1	0	1
280	0	0	2
281	1	0	1
282	0	0	1
283	1	2	0
284	1	2	0
285	0	1	1

286	0	0	0
287	1	1	0
288	0	0	1
289	2	0	1
290	1	1	2
291	1	1	2
292	0	0	1
293	0	1	1
294	0	0	1
295	3	0	2
296	0	1	0
297	0	0	1
298	0	0	1
299	1	1	1
300	0	0	1
301	1	1	0
302	0	1	2
303	0	1	1
304	0	1	2
305	1	0	1
306	0	1	1
307	1	1	0
308	2	0	2
309	1	2	2
310	0	1	0
311	1	1	0
312	0	1	1
313	0	0	0
314	0	2	0
315	1	0	0
316	0	1	0
317	0	0	0
318	0	1	0
319	0	1	0
320	1	0	0
321	1	0	0
322	0	1	0
323	0	3	0
324	0	0	1
325	0	1	1
326	0	1	1
327	0	0	0
328	0	0	0
329	0	0	0
330	0	0	0
331	0	0	1
332	0	0	0

333	0	0	1
334	1	0	0
335	0	0	1
336	0	0	2
337	1	0	0
338	0	0	0
339	1	0	0
340	0	0	0
341	0	0	0
342	0	0	0
343	0	1	0
344	0	0	0
345	0	0	0
346	0	0	0
347	1	0	0
348	0	1	1
349	0	0	0
350	0	2	0
351	0	0	0
352	0	0	0
353	0	0	1
354	0	0	0
355	0	0	1
356	0	0	0
357	0	0	0
358	1	0	0
359	1	0	0
360	0	1	0
361	0	1	0
362	0	0	0
363	0	1	0
364	0	0	0
365	1	0	0
366	0	0	0
367	0	1	0
368	0	0	0
369	0	0	0
370	0	0	0
371	1	0	0
372	0	0	0
373	0	0	0
374	0	0	0
375	0	0	0
376	0	0	0
377	0	0	0
378	0	0	0
379	0	0	0

380	0	1	0
381	0	0	0
382	0	0	0
383	1	0	0
384	0	0	0
385	0	0	0
386	0	0	0
387	0	0	0
388	0	0	0
389	0	0	0
390	0	0	0
391	0	0	0
392	0	0	0
393	0	0	0
394	0	0	0
395	0	0	0
396	0	0	0
397	0	0	0
398	0	0	0
399	0	0	1
400	0	0	0
401	1	1	0
402	0	0	0
403	0	0	0
404	0	0	0
405	0	0	0
406	0	0	0
407	0	0	0
408	0	0	0
409	0	0	0
410	0	0	0
411	0	0	0
412	0	0	0
413	0	0	0
414	0	0	0
415	0	0	0
416	0	0	0
417	0	0	0
418	0	0	0
419	0	0	0
420	0	1	1
421	0	0	
422	0	0	
423	0	0	
424	0	0	
425	0	0	
426	0	0	

427	0	1
428	0	
429	0	
430	0	
431	0	
432	0	
433	0	
434	0	
435	0	
436	0	
437	0	
438	0	
439	0	
440	0	
441	1	

	Broccoli	Root	Plant 2
	Run 1	Run 2	Run 3
Diameter (nm)	Frequency	Frequency	Frequency
34	0	0	0
35	0	0	0
36	0	0	0
37	0	0	0
38	0	0	0
39	0	0	0
40	0	0	0
41	0	0	0
42	0	0	0
43	2	11	7
44	0	0	0
45	0	0	0
46	0	0	0
47	0	0	0
48	0	0	0
49	24	33	30
50	0	0	0
51	0	0	0
52	0	0	0
53	0	0	0
54	28	41	45
55	0	0	0
56	0	0	0
57	0	0	0
58	38	47	53
59	0	0	0

60	0	0	0
61	51	43	55
62	0	0	0
63	0	0	0
64	0	0	0
65	51	75	74
66	0	0	0
67	0	0	0
68	54	95	77
69	0	0	0
70	76	102	104
71	0	0	0
72	0	0	0
73	68	67	63
74	0	0	0
75	52	81	63
76	0	0	0
77	49	59	46
78	0	0	0
79	38	28	35
80	0	0	0
81	39	36	25
82	0	0	0
83	31	29	27
84	0	0	0
85	29	22	27
86	0	0	0
87	20	21	25
88	0	0	0
89	19	19	20
90	17	21	16
91	0	0	0
92	13	14	17
93	16	13	22
94	0	0	0
95	14	18	8
96	19	15	13
97	17	16	9
98	0	0	0
99	9	11	17
100	12	7	10
101	11	7	11
102	0	0	0
103	16	8	13
104	9	13	15
105	1	8	9
106	10	9	9

107	9	9	7
108	8	9	6
109	5	7	7
110	0	0	0
111	11	8	7
112	9	7	5
113	10	9	12
114	5	7	6
115	10	14	8
116	20	14	10
117	8	5	4
118	7	6	4
119	6	8	6
120	8	4	3
121	7	4	7
122	7	5	6
123	6	5	8
124	8	16	13
125	3	5	4
126	3	5	5
127	3	4	6
128	3	10	8
129	4	5	2
130	5	7	4
131	3	1	3
132	13	13	8
133	3	3	3
134	7	8	13
135	0	2	7
136	4	2	3
137	7	5	4
138	4	1	2
139	7	5	9
140	7	5	5
141	7	6	8
142	7	6	8
143	4	2	2
144	8	5	4
145	0	0	3
146	7	2	3
147	8	6	3
148	4	2	3
149	5	3	3
150	6	9	5
151	8	6	1
152	3	2	4
153	6	5	4

154	0	5	3
155	5	3	5
156	9	4	4
157	10	3	5
158	7	4	8
159	7	3	3
160	2	6	3
161	5	3	7
162	7	3	2
163	2	5	4
164	7	5	4
165	6	3	4
166	4	3	6
167	6	6	3
168	7	8	2
169	1	2	2
170	2	5	0
171	4	2	2
172	5	6	4
173	1	4	6
174	2	3	3
175	3	3	2
176	4	1	3
177	8	5	2
178	6	1	5
179	3	3	3
180	3	2	4
181	6	3	1
182	2	0	3
183	8	2	3
184	4	2	2
185	6	4	2
186	2	2	4
187	3	0	3
188	4	2	3
189	2	1	2
190	6	4	1
191	3	2	1
192	0	2	1
193	3	0	3
194	4	5	3
195	1	1	0
196	5	2	1
197	1	2	3
198	3	2	5
199	3	3	5
200	2	4	4

201	0	1	0
202	3	4	1
203	0	1	0
204	0	0	1
205	4	4	3
206	0	2	2
207	0	6	1
208	0	1	0
209	0	0	1
210	0	0	2
211	3	2	3
212	1	2	1
213	2	1	2
214	0	2	2
215	0	2	1
216	1	2	2
217	1	1	1
218	0	1	2
219	3	1	3
220	1	3	0
221	0	0	1
222	2	0	2
223	2	2	0
224	1	0	0
225	1	3	1
226	3	0	0
227	2	1	3
228	0	0	0
229	1	0	0
230	1	3	1
231	0	1	1
232	0	1	1
233	1	0	0
234	0	0	2
235	0	1	2
236	2	1	3
237	3	0	1
238	3	1	0
239	0	3	1
240	0	1	0
241	0	1	3
242	0	0	0
243	1	0	1
244	3	1	1
245	0	3	2
246	0	2	0
247	2	1	1

248	0	0	2
249	3	1	0
250	0	0	0
251	1	0	1
252	1	0	0
253	1	1	0
254	0	0	0
255	1	0	0
256	0	1	0
257	0	0	1
258	2	0	0
259	0	0	0
260	1	0	0
261	0	0	1
262	0	1	1
263	0	0	1
264	1	0	2
265	1	0	3
266	0	1	1
267	0	1	0
268	1	0	0
269	0	1	0
270	0	0	1
271	2	1	0
272	0	1	0
273	0	0	0
274	0	0	0
275	1	1	0
276	0	0	0
277	0	0	0
278	1	1	1
279	0	0	0
280	0	0	0
281	0	1	1
282	0	1	0
283	1	0	1
284	0	0	1
285	0	1	0
286	0	0	0
287	0	1	0
288	1	0	0
289	0	0	1
290	0	0	0
291	0	0	0
292	0	0	0
293	0	0	0
294	1	0	1

295	0	0	1
296	1	0	1
297	1	1	0
298	0	0	0
299	0	0	0
300	1	0	0
301	0	0	0
302	0	0	1
303	0	0	0
304	1	0	0
305	0	1	1
306	0	0	0
307	1	0	0
308	0	0	1
309	0	0	0
310	0	0	2
311	0	0	1
312	0	0	0
313	0	1	0
314	0	0	1
315	0	0	0
316	1	0	0
317	0	0	0
318	0	0	0
319	0	0	0
320	0	0	0
321	0	0	0
322	0	0	0
323	1	0	0
324	0	0	0
325	0	1	0
326	0	0	1
327	1	0	0
328	1	1	0
329	0	0	0
330	0	0	0
331	0	0	0
332	1	0	1
333	0	0	0
334	0	0	0
335	0	0	0
336	0	0	0
337	0	0	0
338	0	0	0
339	0	0	0
340	0	0	0
341	2	0	0

342	0	0	0
343	1	0	0
344	0	0	0
345	0	0	0
346	1	0	0
347	0	0	0
348	0	0	1
349	0	0	1
350	0	0	0
351	0	0	0
352	0	0	0
353	0	1	0
354	0	0	0
355	0	0	0
356	0	0	0
357	1	0	0
358	1	0	0
359	0	0	0
360	0	0	0
361	1	0	0
362	0	0	0
363	0	0	1
364	0	0	0
365	0	0	0
366	0	0	0
367	0	0	0
368	0	0	0
369	1	0	1
370	0	0	0
371	0	1	0
372	0	0	0
373	0	0	0
374	0	0	0
375	0	0	0
376	1	0	0
377	0	0	0
378	0	0	0
379	0	0	0
380	1	0	0
381	0	0	0
382	0	0	1
383	0	0	0
384	0	0	0
385	0	0	0
386	0	0	0
387	0	1	0
388	0	0	0

389	0	0	0
390	0	1	1
391	0	0	
392	0	0	
393	0	0	
394	0	0	
395	0	0	
396	0	0	
397	0	0	
398	0	0	
399	0	0	
400	0	0	
401	0	0	
402	0	0	
403	0	0	
404	0	0	
405	0	0	
406	0	0	
407	0	0	
408	0	0	
409	0	0	
410	0	0	
411	0	0	
412	0	0	
413	0	0	
414	0	0	
415	0	0	
416	0	1	
417	0		
418	0		
419	0		
420	0		
421	0		
422	0		
423	0		
424	0		
425	0		
426	0		
427	0		
428	1		
429	0		
430	0		
431	0		
432	0		
433	0		
434	1		
435	0		

436	0
437	0
438	1
439	0
440	0
441	0
442	0
443	0
444	0
445	0
446	0
447	0
448	0
449	0
450	0
451	0
452	0
453	0
454	0
455	0
456	0
457	0
458	0
459	0
460	0
461	0
462	0
463	0
464	0
465	0
466	0
467	0
468	0
469	0
470	0
471	0
472	0
473	0
474	0
475	0
476	0
477	0
478	0
479	0
480	0
481	0
482	0

483	0
484	0
485	0
486	0
487	0
488	0
489	0
490	0
491	0
492	0
493	0
494	0
495	0
496	0
497	0
498	0
499	0
500	0
501	0
502	0
503	0
504	1
505	0
506	0
507	0
508	0
509	0
510	0
511	0
512	0
513	0
514	0
515	0
516	0
517	0
518	0
519	0
520	0
521	0
522	0
523	0
524	0
525	0
526	0
527	0
528	0
529	0

530	0
531	1

	Broccoli	Root	Plant 3
	Run 1	Run 2	Run 3
Diameter (nm)	Frequency	Frequency	Frequency
34	0	0	0
35	0	0	0
36	0	0	0
37	0	0	0
38	0	0	0
39	0	0	0
40	0	0	0
41	0	0	0
42	0	0	0
43	96	85	87
44	0	0	0
45	0	0	0
46	0	0	0
47	0	0	0
48	0	0	0
49	319	297	272
50	0	0	0
51	0	0	0
52	0	0	0
53	0	0	0
54	267	276	259
55	0	0	0
56	0	0	0
57	0	0	0
58	288	318	307
59	0	0	0
60	0	0	0
61	231	252	246
62	0	0	0
63	0	0	0
64	0	0	0
65	220	212	228
66	0	0	0
67	0	0	0
68	184	182	208
69	0	0	0
70	180	161	167
71	0	0	0
72	0	0	0

73	148	132	171
74	0	0	0
75	106	114	132
76	0	0	0
77	128	142	126
78	0	0	0
79	121	107	102
80	0	0	0
81	85	89	95
82	0	0	0
83	85	86	87
84	0	0	0
85	82	74	68
86	0	0	0
87	72	73	78
88	0	0	0
89	61	61	88
90	61	55	60
91	0	0	0
92	50	61	54
93	53	60	45
94	0	0	0
95	49	56	63
96	48	54	43
97	58	56	47
98	0	0	0
99	47	43	52
100	49	46	48
101	47	47	46
102	0	0	0
103	38	49	50
104	34	45	39
105	45	44	44
106	40	41	42
107	27	50	39
108	21	36	40
109	39	38	37
110	0	0	0
111	21	29	38
112	42	42	39
113	32	42	34
114	40	37	32
115	38	38	25
116	63	54	50
117	36	33	27
118	22	26	36
119	30	25	32

120	17	23	31
121	29	35	31
122	21	23	25
123	24	23	29
124	45	60	53
125	27	21	25
126	25	14	24
127	27	23	17
128	41	39	51
129	26	17	19
130	22	18	23
131	25	26	16
132	37	26	46
133	30	18	17
134	40	37	39
135	18	17	20
136	21	14	11
137	30	34	33
138	14	19	20
139	31	34	38
140	15	13	10
141	32	41	39
142	38	21	36
143	10	16	11
144	34	30	30
145	10	16	7
146	35	26	29
147	25	31	35
148	10	7	8
149	26	30	27
150	18	22	24
151	23	19	24
152	13	9	14
153	25	27	11
154	29	25	16
155	19	15	14
156	27	21	13
157	18	16	21
158	19	18	17
159	17	20	19
160	14	18	20
161	21	17	22
162	22	13	13
163	18	20	16
164	15	15	12
165	12	16	16
166	17	7	13

167	9	16	16
168	17	21	14
169	6	18	4
170	10	14	16
171	14	12	9
172	20	22	17
173	10	12	12
174	13	10	7
175	18	21	20
176	11	19	14
177	15	17	13
178	8	7	13
179	16	15	10
180	17	13	12
181	14	17	10
182	14	10	9
183	12	13	16
184	9	7	7
185	15	13	14
186	8	15	13
187	11	8	6
188	7	7	8
189	14	5	9
190	18	7	10
191	11	5	6
192	5	6	7
193	6	10	10
194	4	14	6
195	7	6	8
196	9	7	6
197	10	10	8
198	9	7	4
199	13	12	8
200	13	6	12
201	2	9	11
202	4	6	6
203	9	7	4
204	5	7	3
205	13	11	7
206	9	3	7
207	11	3	8
208	3	7	10
209	4	4	5
210	6	2	5
211	7	5	7
212	7	5	6
213	10	2	4

214	6	4	7
215	10	3	2
216	4	2	7
217	0	3	1
218	8	5	4
219	4	5	5
220	7	3	3
221	4	2	3
222	2	3	4
223	4	7	4
224	7	8	4
225	0	6	7
226	8	3	7
227	3	5	3
228	5	4	5
229	4	4	5
230	5	2	3
231	1	4	3
232	3	1	7
233	4	3	3
234	3	1	2
235	2	6	2
236	5	6	2
237	2	4	2
238	4	0	2
239	3	4	5
240	4	2	3
241	3	4	3
242	1	6	4
243	6	4	2
244	1	2	5
245	2	1	3
246	0	2	1
247	3	2	0
248	2	2	2
249	1	1	2
250	3	5	2
251	1	3	2
252	1	0	2
253	2	1	3
254	1	0	3
255	1	2	4
256	0	3	0
257	1	2	1
258	1	0	1
259	0	1	4
260	1	2	2

261	3	1	1
262	5	0	1
263	1	3	2
264	4	0	0
265	1	2	0
266	1	2	1
267	0	1	5
268	1	1	1
269	0	1	0
270	0	1	0
271	1	3	2
272	0	2	1
273	1	2	2
274	1	2	0
275	1	3	2
276	2	1	0
277	1	0	1
278	1	2	0
279	1	0	0
280	2	0	2
281	2	4	1
282	1	1	3
283	2	0	0
284	2	0	2
285	0	0	1
286	0	0	0
287	1	4	0
288	0	1	0
289	1	0	0
290	0	0	1
291	1	2	3
292	1	0	0
293	0	2	1
294	0	1	1
295	0	0	1
296	3	0	1
297	1	0	0
298	2	0	1
299	1	1	0
300	0	0	1
301	1	3	0
302	0	0	2
303	0	0	0
304	1	1	2
305	0	3	0
306	1	0	1
307	0	1	2

308	2	1	1
309	0	1	0
310	1	1	0
311	2	0	0
312	0	0	0
313	0	0	0
314	0	0	0
315	0	0	0
316	0	0	0
317	0	2	0
318	1	0	1
319	0	0	0
320	0	1	0
321	1	0	0
322	0	1	0
323	0	0	1
324	0	0	1
325	0	0	0
326	1	1	0
327	0	0	0
328	0	0	1
329	0	0	0
330	0	0	0
331	1	1	0
332	0	0	1
333	0	0	1
334	0	0	0
335	0	0	0
336	0	0	0
337	0	0	0
338	2	0	0
339	1	1	0
340	0	0	0
341	0	0	0
342	0	0	1
343	0	0	0
344	0	0	0
345	0	0	0
346	0	1	0
347	1	1	0
348	0	0	0
349	0	0	0
350	1	0	0
351	0	0	0
352	0	1	0
353	1	0	0
354	2	0	0

355	0	0	0
356	0	0	0
357	0	0	0
358	0	0	0
359	1	1	0
360	0	0	1
361	0	0	0
362	0	0	1
363	0	0	0
364	0	0	0
365	0	0	0
366	0	0	0
367	0	0	1
368	0	0	0
369	0	0	0
370	0	0	0
371	0	0	0
372	0	0	0
373	1	1	1
374	0	0	0
375	0	0	0
376	0	0	0
377	0	0	0
378	0	0	0
379	0	0	0
380	0	0	0
381	0	1	0
382	0	0	1
383	0	0	0
384	0	1	0
385	0	0	0
386	0	0	0
387	1	0	0
388	0	1	0
389	0	0	0
390	0	0	0
391	0	0	0
392	0	0	0
393	0	0	1
394	0	0	0
395	0	0	0
396	0	0	0
397	0	0	1
398	0	0	0
399	0	0	0
400	0	0	0
401	0	0	0

402	0	0	0
403	0	0	0
404	0	0	0
405	0	1	0
406	0	1	1
407	0	0	0
408	0	0	0
409	0	0	0
410	0	0	0
411	0	0	0
412	0	0	0
413	0	0	0
414	0	0	0
415	0	0	0
416	0	0	0
417	0	0	0
418	0	0	0
419	0	0	0
420	0	0	0
421	0	0	0
422	0	0	0
423	0	0	1
424	0	0	0
425	0	0	0
426	0	0	0
427	0	0	0
428	0	0	0
429	0	0	0
430	0	0	0
431	0	0	0
432	0	0	0
433	0	0	0
434	0	0	0
435	0	0	0
436	0	0	0
437	0	0	0
438	0	0	0
439	0	0	0
440	0	0	0
441	0	0	0
442	0	0	0
443	0	0	0
444	0	0	0
445	0	0	0
446	0	0	0
447	0	0	0
448	0	0	0

449	0	0	0
450	1	0	0
451		1	0
452			0
453			0
454			0
455			0
456			0
457			0
458			0
459			0
460			0
461			0
462			0
463			0
464			0
465			0
466			0
467			0
468			0
469			0
470			0
471			0
472			0
473			0
474			0
475			0
476			0
477			1
478			0
479			0
480			0
481			0
482			0
483			0
484			0
485			0
486			0
487			0
488			0
489			0
490			0
491			0
492			0
493			0
494			0
495			0

496	0
497	0
498	0
499	0
500	0
501	0
502	0
503	0
504	0
505	0
506	0
507	0
508	0
509	0
510	0
511	0
512	0
513	0
514	0
515	0
516	0
517	0
518	0
519	0
520	0
521	0
522	0
523	0
524	0
525	0
526	0
527	0
528	0
529	0
530	0
531	0
532	0
533	0
534	0
535	0
536	1

	Broccoli	Shoot	Plant 1
	Run 1	Run 2	Run 3
Diameter (nm)	Frequency	Frequency	Frequency

34	0	0	0
35	0	0	0
36	0	0	0
37	0	0	0
38	0	0	0
39	0	0	0
40	0	0	0
41	0	0	0
42	0	0	0
43	34	47	158
44	0	0	0
45	0	0	0
46	0	0	0
47	0	0	0
48	0	0	0
49	92	96	364
50	0	0	0
51	0	0	0
52	0	0	0
53	0	0	0
54	112	122	711
55	0	0	0
56	0	0	0
57	0	0	0
58	142	192	158
59	0	0	0
60	0	0	0
61	42	55	54
62	0	0	0
63	0	0	0
64	0	0	0
65	14	11	29
66	0	0	0
67	0	0	0
68	8	6	6
69	0	0	0
70	3	2	4
71	0	0	0
72	0	0	0
73	2	2	0
74	0	0	0
75	0	0	2
76	0	0	0
77	1	2	0
78	0	0	0
79	0	0	0
80	0	0	0

81	0	0	1
82	0	0	0
83	1	1	0
84	0		0
85	0		0
86	0		0
87	0		0
88	0		0
89	0		0
90	0		1
91	0		
92	0		
93	0		
94	0		
95	1		

	Broccoli	Shoot	Plant 2
	Run 1	Run 2	Run 3
Diameter (nm)	Frequency	Frequency	Frequency
34	0	0	0
35	0	0	0
36	0	0	0
37	0	0	0
38	0	0	0
39	0	0	0
40	0	0	0
41	0	0	0
42	0	0	0
43	27	114	115
44	0	0	0
45	0	0	0
46	0	0	0
47	0	0	0
48	0	0	0
49	45	250	222
50	0	0	0
51	0	0	0
52	0	0	0
53	0	0	0
54	57	506	427
55	0	0	0
56	0	0	0
57	0	0	0
58	89	133	125
59	0	0	0

60	0	0	0
61	27	48	38
62	0	0	0
63	0	0	0
64	0	0	0
65	12	13	7
66	0	0	0
67	0	0	0
68	0	6	3
69	0	0	0
70	1	1	1
71	0	0	0
72	0	0	0
73	0	1	0
74	0	0	0
75	1	0	0
76	0	0	0
77	0	0	0
78	0	0	0
79	0	0	0
80	0	0	0
81	0	0	0
82	0	0	0
83	1	0	0
84	0	0	0
85	0	0	0
86	0	0	0
87	0	0	0
88	0	0	0
89	0	1	0
90	0	0	0
91	0	0	0
92	0	0	0
93	1	0	0
94	0	0	0
95	0	0	0
96	0	0	0
97	0	0	0
98	0	0	0
99	0	0	0
100	0	0	0
101	0	0	0
102	0	0	0
103	0	0	0
104	0	0	0
105	1	0	0
106		0	0

107		0	0
108		0	0
109		0	0
110		0	0
111		0	0
112		1	0
113		0	0
114		0	0
115		0	0
116		0	1
117		0	
118		0	
119		0	
120		0	
121		0	
122		0	
123		0	
124		0	
125		0	
126		0	
127		0	
128		0	
129		0	
130		0	
131		0	
132		0	
133		0	
134		0	
135		0	
136		1	

	Broccoli	Shoot	Plant 3
	Run 1	Run 2	Run 3
Diameter (nm)	Frequency	Frequency	Frequency
34	0	1	61
35	0	0	0
36	0	0	0
37	0	0	0
38	0	0	0
39	0	0	0
40	0	0	0
41	0	0	0
42	0	0	0
43	166	39	396
44	0	0	0

45	0	0	0
46	0	0	0
47	0	0	0
48	0	0	0
49	566	71	637
50	0	0	0
51	0	0	0
52	0	0	0
53	0	0	0
54	94	62	106
55	0	0	0
56	0	0	0
57	0	0	0
58	18	15	18
59	0	0	0
60	0	0	0
61	2	2	2
62	0	0	0
63	0	0	0
64	0	0	0
65	1	1	3
66		0	0
67		0	0
68		1	0
69		0	0
70		1	0
71		0	0
72		0	0
73		0	0
74		0	0
75		0	1
76		0	0
77		0	0
78		0	0
79		1	0
80		0	0
81		0	0
82		0	0
83		0	0
84		0	0
85		0	0
86		0	0
87		0	0
88		0	0
89		0	1
90		0	
91		0	

92	0
93	0
94	0
95	0
96	0
97	0
98	0
99	0
100	0
101	0
102	0
103	0
104	0
105	0
106	0
107	0
108	0
109	0
110	0
111	0
112	0
113	0
114	0
115	0
116	0
117	0
118	0
119	0
120	0
121	0
122	0
123	0
124	0
125	0
126	0
127	0
128	0
129	0
130	0
131	0
132	0
133	0
134	0
135	0
136	0
137	0
138	0

139	0
140	0
141	0
142	0
143	0
144	0
145	0
146	0
147	0
148	0
149	0
150	0
151	0
152	0
153	0
154	0
155	0
156	0
157	0
158	0
159	0
160	1

	Lamb's Lettuce	Root	Plant 1
	Run 1	Run 2	Run 3
Diameter (nm)	Frequency	Frequency	Frequency
36	33	0	781
37	0	0	0
38	0	0	0
39	0	0	0
40	0	0	0
41	0	0	0
42	0	0	0
43	0	0	0
44	0	0	0
45	310	16	4424
46	0	0	0
47	0	0	0
48	0	0	0
49	0	0	0
50	0	0	0
51	0	0	0
52	528	21	421
53	0	0	0
54	0	0	0

55	0	0	0
56	0	0	0
57	50	33	29
58	0	0	0
59	0	0	0
60	0	0	0
61	5	3	4
62	0	0	0
63	0	0	0
64	0	0	0
65	2	1	0
66	0	0	0
67	0	0	0
68	0	1	1
69	0	0	
70	0	0	
71	0	0	
72	0	0	
73	0	0	
74	0	0	
75	0	0	
76	0	0	
77	0	0	
78	0	0	
79	0	0	
80	0	0	
81	0	0	
82	0	0	
83	0	0	
84	0	0	
85	0	0	
86	0	0	
87	0	0	
88	0	0	
89	0	0	
90	0	0	
91	0	0	
92	0	0	
93	0	0	
94	0	0	
95	0	0	
96	0	0	
97	0	0	
98	0	0	
99	0	0	
100	0	0	
101	0	0	

102	0	0
103	0	0
104	0	0
105	0	0
106	0	0
107	0	0
108	0	0
109	0	0
110	0	0
111	0	0
112	0	0
113	0	0
114	0	0
115	0	0
116	0	0
117	0	0
118	0	0
119	0	0
120	0	0
121	0	0
122	0	0
123	0	0
124	0	1
125	0	
126	0	
127	0	
128	0	
129	0	
130	0	
131	0	
132	0	
133	0	
134	0	
135	0	
136	0	
137	0	
138	0	
139	0	
140	1	

	Lamb's Lettuce	Root	Plant 2
	Run 1	Run 2	Run 3
Diameter (nm)	Frequency	Frequency	Frequency
36	266	37493	227
37	0	0	0

38	0	0	0
39	0	0	0
40	0	0	0
41	0	0	0
42	0	0	0
43	0	0	0
44	0	0	0
45	1898	1597	1676
46	0	0	0
47	0	0	0
48	0	0	0
49	0	0	0
50	0	0	0
51	0	0	0
52	117	128	105
53	0	0	0
54	0	0	0
55	0	0	0
56	0	0	0
57	9	4	5
58	0	0	0
59	0	0	0
60	0	0	0
61	1	1	1
62			0
63			0
64			0
65			0
66			0
67			0
68			0
69			0
70			0
71			0
72			1
73			0
74			0
75			0
76			0
77			0
78			0
79			0
80			0
81			0
82			0
83			0
84			0

85			0
86			1

	Lamb's Lettuce	Root	Plant 3
	Run 1	Run 2	Run 3
Diameter (nm)	Frequency	Frequency	Frequency
36	390	375	44768
37	0	0	0
38	0	0	0
39	0	0	0
40	0	0	0
41	0	0	0
42	0	0	0
43	0	0	0
44	0	0	0
45	2470	2489	2191
46	0	0	0
47	0	0	0
48	0	0	0
49	0	0	0
50	0	0	0
51	0	0	0
52	186	166	152
53	0	0	0
54	0	0	0
55	0	0	0
56	0	0	0
57	8	12	4
58	0	0	0
59	0	0	0
60	0	0	0
61	3	3	2

	Lamb's Lettuce	Shoot	Plant 1
	Run 1	Run 2	Run 3
Diameter (nm)	Frequency	Frequency	Frequency
36	174	177	158
37	0	0	0
38	0	0	0
39	0	0	0
40	0	0	0
41	0	0	0
42	0	0	0

43	0	0	0
44	0	0	0
45	1386	1340	1334
46	0	0	0
47	0	0	0
48	0	0	0
49	0	0	0
50	0	0	0
51	0	0	0
52	75	81	60
53	0	0	0
54	0	0	0
55	0	0	0
56	0	0	0
57	3	4	5
58			0
59			0
60			0
61			0
62			0
63			0
64			0
65			1

	Lamb's Lettuce	Shoot	Plant 2
	Run 1	Run 2	Run 3
Diameter (nm)	Frequency	Frequency	Frequency
36	25223	24275	23848
37	0	0	0
38	0	0	0
39	0	0	0
40	0	0	0
41	0	0	0
42	0	0	0
43	0	0	0
44	0	0	0
45	937	862	857
46	0	0	0
47	0	0	0
48	0	0	0
49	0	0	0
50	0	0	0
51	0	0	0
52	53	44	42
53	0	0	

54	0	0
55	0	0
56	0	0
57	1	1

	Lamb's Lettuce	Shoot	Plant 3
	Run 1	Run 2	Run 3
Diameter (nm)	Frequency	Frequency	Frequency
36	12223	11892	12051
37	0	0	0
38	0	0	0
39	0	0	0
40	0	0	0
41	0	0	0
42	0	0	0
43	0	0	0
44	0	0	0
45	383	311	357
46	0	0	0
47	0	0	0
48	0	0	0
49	0	0	0
50	0	0	0
51	0	0	0
52	15	14	17
53	0	0	
54	0	0	
55	0	0	
56	0	0	
57	0	1	
58	0		
59	0		
60	0		
61	0		
62	0		
63	0		
64	0		
65	0		
66	0		
67	0		
68	0		
69	0		
70	0		
71	0		
72	0		

73	0
74	0
75	0
76	0
77	0
78	0
79	0
80	0
81	0
82	0
83	0
84	0
85	0
86	0

	Brazil Nut	Batch 1	Nut 1
	Run 1	Run 2	Run 3
Diameter (nm)	Frequency	Frequency	Frequency
30	0	0	0
31	0	0	0
32	0	0	0
33	0	0	0
34	0	0	0
35	0	0	0
36	0	0	0
37	0	0	0
38	83	3	488
39	0	0	0
40	0	0	0
41	0	0	0
42	0	0	0
43	320	2	1041
44	0	0	0
45	0	0	0
46	0	0	0
47	0	0	0
48	374	0	1874
49	0	0	0
50	0	0	0
51	267	3	683
52	0	0	0
53	0	0	0
54	0	0	0
55	55	3	248
56	0	0	0

57	7	4	80
58	0	0	0
59	0	0	0
60	0	1	26
61	0	0	0
62	0	0	7
63	0	0	0
64	0	0	0
65	0	0	3
66	0	0	0
67	0	1	0
68	0	0	0
69	0	0	0
70	0	0	0
71	0	0	1
72	0	1	
73	0	0	
74	2	0	
75		0	
76		0	
77		0	
78		0	
79		0	
80		1	
81		0	
82		1	
83		1	
84		0	
85		0	
86		0	
87		0	
88		0	
89		0	
90		0	
91		0	
92		0	
93		0	
94		0	
95		0	
96		0	
97		0	
98		0	
99		0	
100		0	
101		0	
102		0	
103		0	

104	0
105	0
106	0
107	0
108	0
109	0
110	0
111	0
112	0
113	0
114	1
115	0
116	0
117	0
118	0
119	0
120	0
121	0
122	0
123	0
124	0
125	0
126	0
127	0
128	0
129	0
130	0
131	0
132	0
133	0
134	0
135	0
136	0
137	0
138	0
139	0
140	0
141	0
142	0
143	0
144	0
145	0
146	0
147	0
148	0
149	0
150	0

151		0	
152		0	
153		0	
154		0	
155		0	
156		1	

	Brazil Nut	Batch 1	Nut 2
	Run 1	Run 2	Run 3
Diameter (nm)	Frequency	Frequency	Frequency
30	0	0	0
31	0	0	0
32	0	0	0
33	0	0	0
34	0	0	0
35	0	0	0
36	0	0	0
37	0	0	0
38	10	1	10
39	0	0	0
40	0	0	0
41	0	0	0
42	0	0	0
43	33	3	25
44	0	0	0
45	0	0	0
46	0	0	0
47	0	0	0
48	36	5	35
49	0	0	0
50	0	0	0
51	58	5	44
52	0	0	0
53	0	0	0
54	0	0	0
55	11	7	7
56	0	0	0
57	2	0	2
58	0	0	0
59	0	0	0
60	1	1	2
61	0	0	0
62	0	0	2
63	0	0	0
64	0	0	0

65	1	0	0
66	0	0	0
67	0	0	1
68	0	0	0
69	1	0	0
70	0	0	0
71	1	0	0
72		0	0
73		0	0
74		0	0
75		0	0
76		0	0
77		0	1
78		0	
79		0	
80		0	
81		0	
82		0	
83		1	

	Brazil Nut	Batch 1	Nut 3
	Run 1	Run 2	Run 3
Diameter (nm)	Frequency	Frequency	Frequency
30	0	0	0
31	0	0	0
32	0	0	0
33	0	0	0
34	0	0	0
35	0	0	0
36	0	0	0
37	0	0	0
38	105	460	23
39	0	0	0
40	0	0	0
41	0	0	0
42	0	0	0
43	101	445	33
44	0	0	0
45	0	0	0
46	0	0	0
47	0	0	0
48	96	296	24
49	0	0	0
50	0	0	0
51	42	42	12

52	0	0	0
53	0	0	0
54	0	0	0
55	13	8	8
56	0	0	0
57	2	5	2
58	0	0	0
59	0	0	0
60	1	0	0
61	0	0	0
62	2	0	0
63	0	0	0
64	0	0	0
65	0	1	0
66	0	0	0
67	0	0	0
68	0	0	0
69	2	0	0
70	0	0	0
71	0	0	0
72	0	0	0
73	0	0	0
74	0	1	0
75	0		0
76	0		1
77	0		0
78	0		0
79	0		1
80	0		0
81	0		0
82	0		0
83	0		0
84	0		0
85	0		0
86	0		0
87	0		0
88	0		0
89	0		0
90	0		0
91	0		0
92	0		0
93	0		0
94	0		0
95	0		0
96	0		0
97	0		0
98	0		0

99	0		0
100	0		0
101	0		0
102	0		0
103	0		0
104	0		0
105	1		0
106	0		2
107	0		
108	0		
109	0		
110	1		

	Brazil Nut	Batch 2	Nut 1
	Run 1	Run 2	Run 3
Diameter (nm)	Frequency	Frequency	Frequency
34	0	0	0
35	0	0	0
36	0	0	0
37	0	0	0
38	0	0	0
39	0	0	0
40	0	0	0
41	0	0	0
42	0	0	0
43	7	6	0
44	0	0	0
45	0	0	0
46	0	0	0
47	0	0	0
48	0	0	0
49	3	9	0
50	0	0	0
51	0	0	0
52	0	0	0
53	0	0	0
54	6	4	0
55	0	0	0
56	0	0	0
57	0	0	0
58	5	3	0
59	0	0	0
60	0	0	0
61	0	0	0
62	2	4	0

63	0	0	0
64	0	0	0
65	0	1	0
66	0	0	0
67	0	0	0
68	0	0	1
69	0	0	0
70	0	0	0
71	1	1	0
72	0	0	0
73	0	0	0
74	0	0	0
75	0	0	0
76	0	0	0
77	0	0	0
78	0	0	0
79	0	0	0
80	0	0	0
81	0	0	0
82	0	1	0
83	0	0	0
84	0	0	0
85	0	0	0
86	0	0	0
87	0	0	0
88	0	1	0
89	0	1	0
90	0	0	0
91	0	0	0
92	0	0	0
93	0	0	0
94	0	0	0
95	0	0	0
96	0	0	0
97	0	0	0
98	0	0	0
99	0	0	0
100	1	0	0
101	0	0	0
102	0	0	0
103	0	0	0
104	0	0	0
105	0	0	0
106	0	0	0
107	0	0	0
108	0	0	0
109	0	0	0

110	0	0	1
111	0	0	0
112	0	0	0
113	0	0	0
114	0	0	0
115	0	0	0
116	0	0	0
117	0	0	0
118	0	0	0
119	0	0	0
120	0	0	0
121	0	0	0
122	0	0	0
123	0	0	0
124	0	0	0
125	0	0	0
126	0	0	0
127	0	0	0
128	0	0	0
129	0	0	0
130	0	0	0
131	0	0	0
132	0	0	0
133	0	0	0
134	0	0	0
135	0	0	0
136	0	0	0
137	0	0	0
138	0	0	0
139	0	0	0
140	0	0	0
141	0	0	0
142	0	0	1
143	0	0	0
144	0	0	0
145	0	0	0
146	0	0	0
147	0	0	0
148	0	0	0
149	0	0	0
150	0	0	0
151	0	0	0
152	0	0	0
153	0	0	0
154	0	0	0
155	0	1	0
156	0	0	0

157	0	1	0
158	1	0	0
159		0	0
160		0	0
161		0	0
162		0	0
163		0	0
164		0	0
165		0	0
166		0	0
167		0	0
168		0	0
169		0	0
170		0	0
171		0	0
172		0	0
173		0	0
174		0	0
175		0	0
176		0	0
177		0	0
178		0	0
179		0	0
180		0	0
181		0	0
182		0	0
183		0	0
184		0	0
185		0	0
186		0	0
187		0	0
188		0	0
189		0	0
190		0	0
191		0	0
192		0	0
193		0	0
194		0	0
195		0	0
196		0	0
197		0	0
198		0	0
199		0	0
200		0	0
201		0	0
202		0	0
203		1	0

204	0
205	0
206	0
207	0
208	0
209	0
210	0
211	0
212	0
213	0
214	0
215	0
216	0
217	0
218	0
219	0
220	0
221	0
222	0
223	0
224	0
225	0
226	0
227	0
228	0
229	0
230	0
231	0
232	0
233	0
234	0
235	0
236	0
237	0
238	0
239	0
240	0
241	0
242	0
243	0
244	0
245	0
246	0
247	0
248	0
249	0
250	0

251			0
252			0
253			0
254			0
255			0
256			0
257			0
258			0
259			0
260			0
261			0
262			0
263			0
264			0
265			0
266			0
267			0
268			0
269			0
270			0
271			0
272			0
273			1

	Brazil Nut	Batch 2	Nut 2
	Run 1	Run 2	Run 3
Diameter (nm)	Frequency	Frequency	Frequency
34	0	0	0
35	0	0	0
36	0	0	0
37	0	0	0
38	0	0	0
39	0	0	0
40	0	0	0
41	0	0	0
42	0	0	0
43	0	0	0
44	0	0	0
45	0	0	0
46	0	0	0
47	0	0	0
48	0	0	0
49	0	0	0
50	0	0	0
51	0	0	0

52	0	0	0
53	0	0	0
54	0	0	0
55	0	0	0
56	0	0	0
57	0	0	0
58	0	0	1
59	0	0	0
60	0	0	0
61	0	0	0
62	0	0	4
63	0	0	0
64	0	0	0
65	1	0	0
66	0	0	0
67	0	0	0
68	0	0	0
69	0	0	0
70	0	0	0
71	0	0	0
72	0	0	0
73	0	0	0
74	0	0	0
75	0	0	0
76	0	0	0
77	0	0	0
78	0	0	0
79	0	0	0
80	0	1	0
81	0	0	0
82	1	0	0
83	0	0	0
84	0	0	0
85	0	0	0
86	0	0	1
87	0	0	0
88	0	0	0
89	0	0	0
90	0	0	0
91	0	0	1
92	0	0	0
93	0	0	0
94	0	2	0
95	0	0	0
96	0	0	0
97	0	0	0
98	0	0	0

99	0	0	0
100	0	0	0
101	0	0	0
102	0	0	0
103	0	0	0
104	0	0	0
105	0	0	0
106	0	0	1
107	0	0	0
108	0	0	0
109	0	0	0
110	0	0	0
111	0	0	0
112	0	0	0
113	0	0	0
114	0	0	0
115	0	0	0
116	0	0	0
117	0	0	0
118	0	0	0
119	0	0	0
120	0	0	0
121	0	0	0
122	0	0	0
123	0	0	0
124	0	0	0
125	0	0	0
126	0	0	0
127	0	0	0
128	0	0	0
129	0	0	0
130	0	0	0
131	0	0	0
132	0	0	0
133	0	0	0
134	0	0	0
135	0	0	0
136	0	0	0
137	0	0	0
138	0	0	0
139	0	0	0
140	0	0	0
141	0	0	0
142	0	0	0
143	0	1	0
144	0	0	0
145	1	0	0

146	0	1
147	0	0
148	0	0
149	0	0
150	0	0
151	0	0
152	0	0
153	0	0
154	0	0
155	0	0
156	0	0
157	0	0
158	0	0
159	0	0
160	0	0
161	0	0
162	0	0
163	0	1
164	0	
165	0	
166	0	
167	0	
168	0	
169	0	
170	0	
171	0	
172	0	
173	0	
174	0	
175	0	
176	0	
177	0	
178	0	
179	0	
180	0	
181	0	
182	0	
183	0	
184	0	
185	0	
186	0	
187	0	
188	0	
189	0	
190	0	
191	0	
192	0	

193	0
194	0
195	0
196	0
197	0
198	0
199	0
200	0
201	0
202	1
203	0
204	0
205	0
206	0
207	0
208	0
209	0
210	0
211	0
212	0
213	0
214	0
215	0
216	0
217	0
218	0
219	0
220	0
221	0
222	0
223	0
224	0
225	0
226	0
227	0
228	0
229	0
230	0
231	1

	Brazil Nut	Batch 2	Nut 3
	Run 1	Run 2	Run 3
Diameter (nm)	Frequency	Frequency	Frequency
34	0	0	0
35	0	0	0

36	0	0	0
37	0	0	0
38	0	0	0
39	0	0	0
40	0	0	0
41	0	0	0
42	0	0	0
43	4	2	8
44	0	0	0
45	0	0	0
46	0	0	0
47	0	0	0
48	0	0	0
49	7	10	27
50	0	0	0
51	0	0	0
52	0	0	0
53	0	0	0
54	8	8	14
55	0	0	0
56	0	0	0
57	0	0	0
58	6	3	13
59	0	0	0
60	0	0	0
61	0	0	0
62	7	3	10
63	0	0	0
64	0	0	0
65	2	5	7
66	0	0	0
67	0	0	0
68	1	5	2
69	0	0	0
70	0	0	0
71	2	0	2
72	0	0	0
73	0	2	0
74	0	0	0
75	0	0	0
76	1	0	2
77	0	0	0
78	0	3	1
79	0	0	0
80	1	1	0
81	0	0	0
82	1	0	0

83	0	0	0
84	2	1	0
85	0	0	0
86	2	0	1
87	0	0	0
88	1	0	0
89	0	0	0
90	0	0	0
91	1	0	0
92	0	0	0
93	1	0	0
94	2	1	0
95	0	0	0
96	1	1	0
97	0	1	0
98	0	0	0
99	0	0	0
100	0	0	0
101	0	1	0
102	0	1	1
103	0	0	0
104	1	0	0
105	0	0	0
106	0	0	0
107	1	0	1
108	0	0	0
109	0	0	0
110	0	0	1
111	0	0	0
112	0	1	0
113	0	0	0
114	0	0	1
115	0	0	0
116	0	0	0
117	0	0	0
118	0	0	0
119	0	1	0
120	0	0	0
121	0	0	0
122	0	0	0
123	0	0	0
124	1	0	0
125	0	0	0
126	0	1	1
127	0	0	
128	0	0	
129	1	0	

130	0	0
131	0	0
132	0	0
133	0	0
134	0	0
135	0	0
136	0	0
137	0	0
138	0	0
139	0	0
140	0	0
141	0	0
142	0	0
143	0	0
144	0	0
145	0	0
146	0	0
147	0	0
148	0	0
149	0	0
150	0	0
151	0	0
152	0	0
153	0	0
154	0	0
155	0	0
156	0	0
157	0	0
158	0	0
159	0	0
160	0	0
161	0	0
162	0	0
163	0	0
164	0	0
165	0	0
166	0	0
167	0	0
168	0	0
169	0	0
170	0	0
171	0	0
172	0	0
173	0	0
174	0	0
175	0	0
176	0	0

177	0	0
178	0	0
179	0	1
180	1	0
181		0
182		0
183		0
184		0
185		0
186		0
187		0
188		0
189		0
190		0
191		0
192		0
193		0
194		0
195		0
196		0
197		0
198		0
199		0
200		0
201		0
202		0
203		0
204		0
205		0
206		0
207		0
208		0
209		0
210		0
211		0
212		0
213		0
214		0
215		0
216		0
217		0
218		0
219		0
220		0
221		0
222		0
223		0

224	0
225	0
226	0
227	0
228	0
229	0
230	0
231	0
232	0
233	0
234	0
235	0
236	0
237	0
238	0
239	0
240	0
241	0
242	0
243	0
244	0
245	0
246	0
247	1
