# Supplementary material for: Ubiquitous Occurrence of Nano Selenium in Food Plants
Source: Foods. 2023 Aug 25;12(17):3203. doi: 10.3390/foods12173203 (PMC10487048; doi:10.3390/foods12173203)
Supplement: Supplementary file 1 [file foods-12-03203-s001.zip › Supplementary information S2 SeNP size histograms.pdf]

Supplementary information S2 for

Ubiquitous occurrence of nano selenium in food plants

Jonas Verstegen, Klaus Günther*
*Corresponding author. Email: k.guenther@fz-juelich.de

To improve conciseness and clarity in visualization the histograms shown in the figures in the paper all display the mean value and standard deviation of the three individual runs that have been performed for every sample in this study. This supplementary information contains all individual data points for the histograms included in figures 1 to 4, i.e. supplementary figures SF1 to SF48.

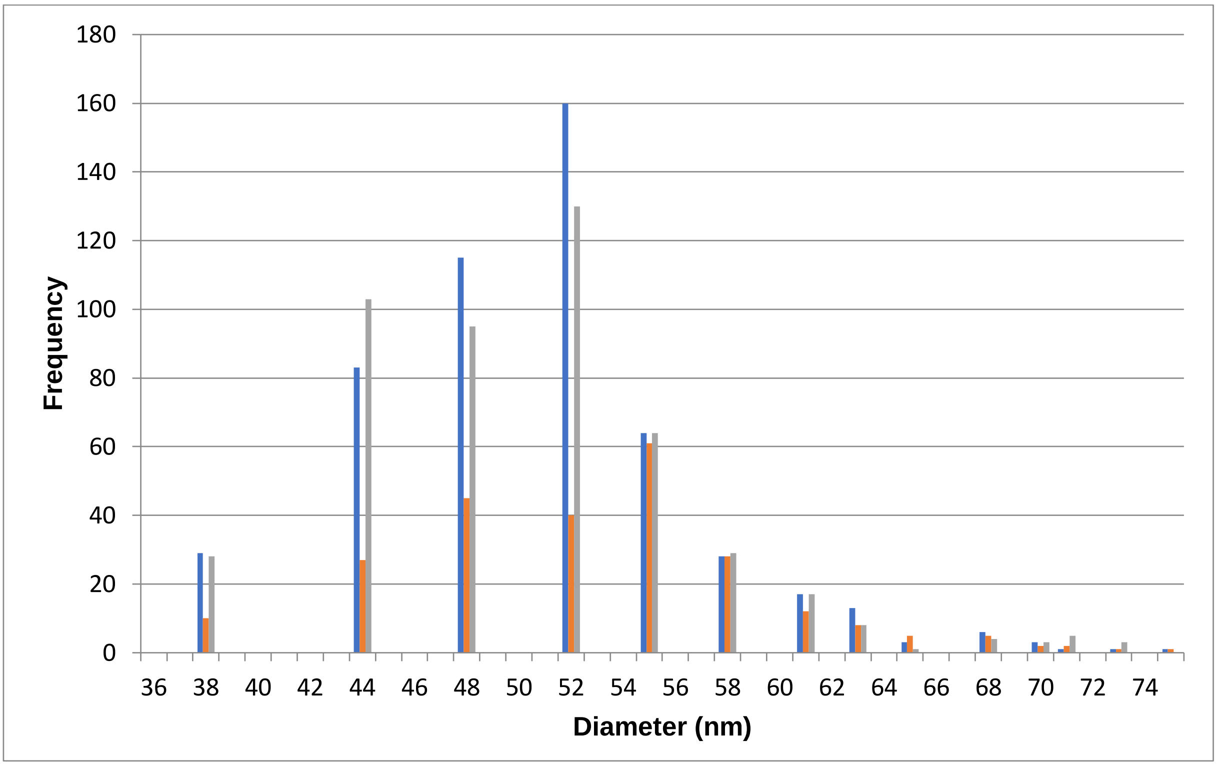

Supplementary figure S1: Size histograms for 3 runs of Basil 1 Root.

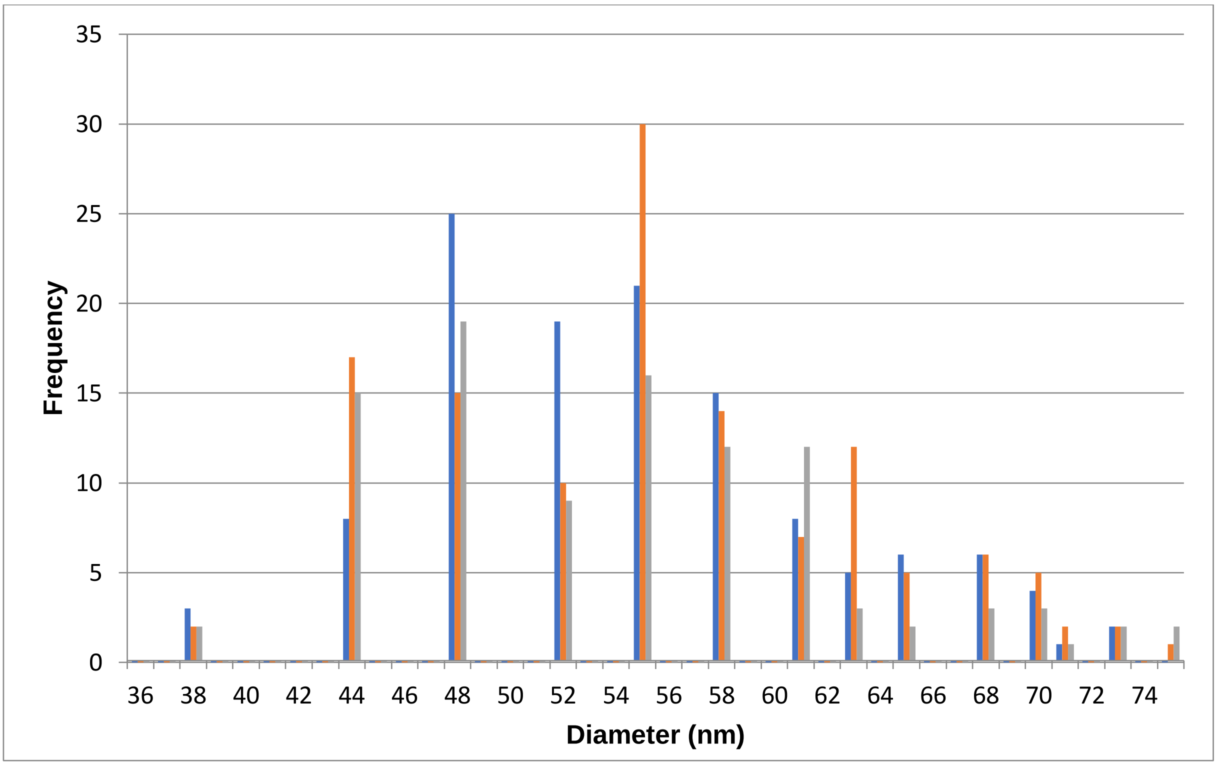

Supplementary figure S2: Size histograms for 3 runs of Basil 2 Root.

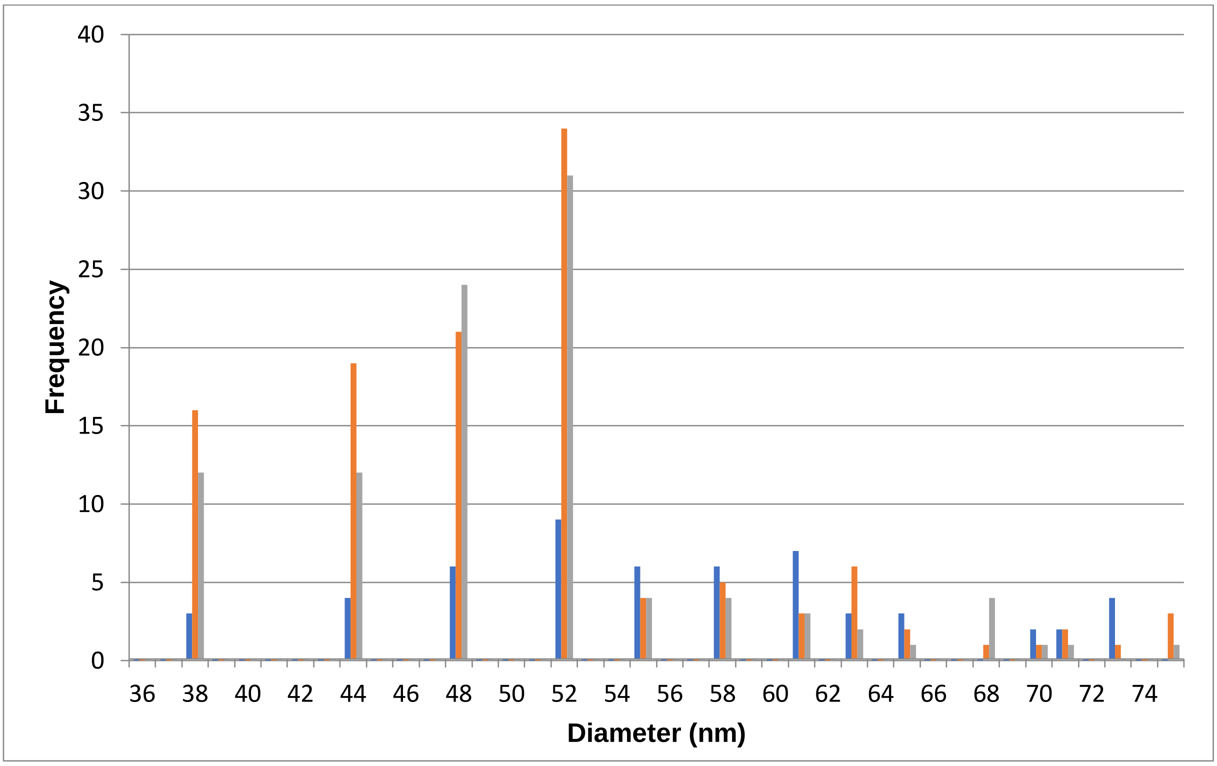

Supplementary figure S3: Size histograms for 3 runs of Basil 3 Root.

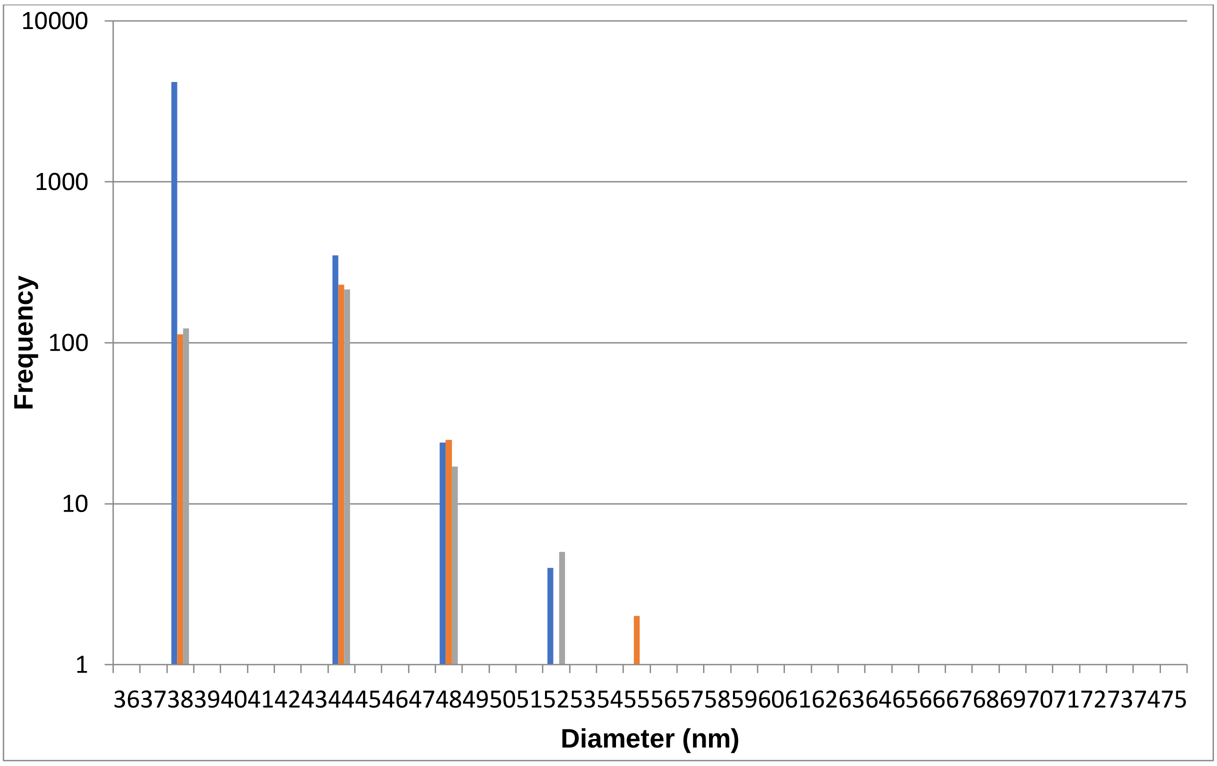

Supplementary figure S4: Size histograms for 3 runs of Basil 1 Shoot.

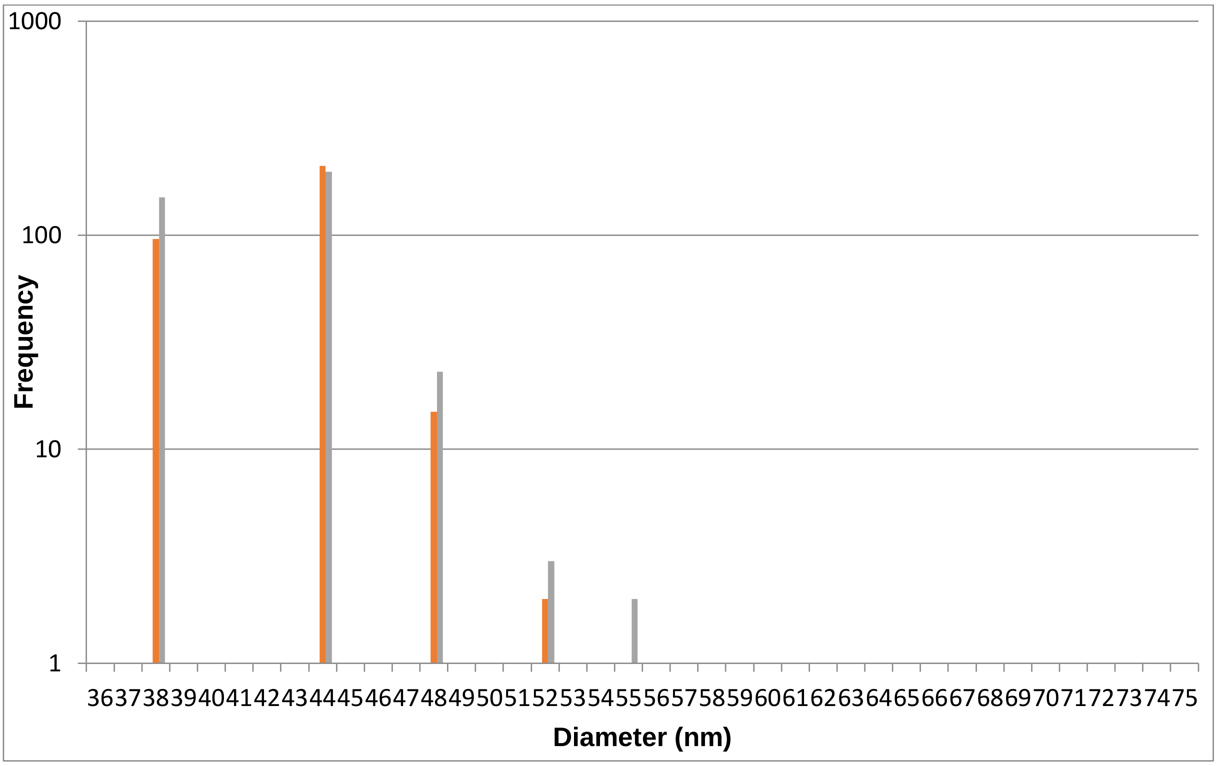

Supplementary figure S5: Size histograms for 3 runs of Basil 2 Shoot.

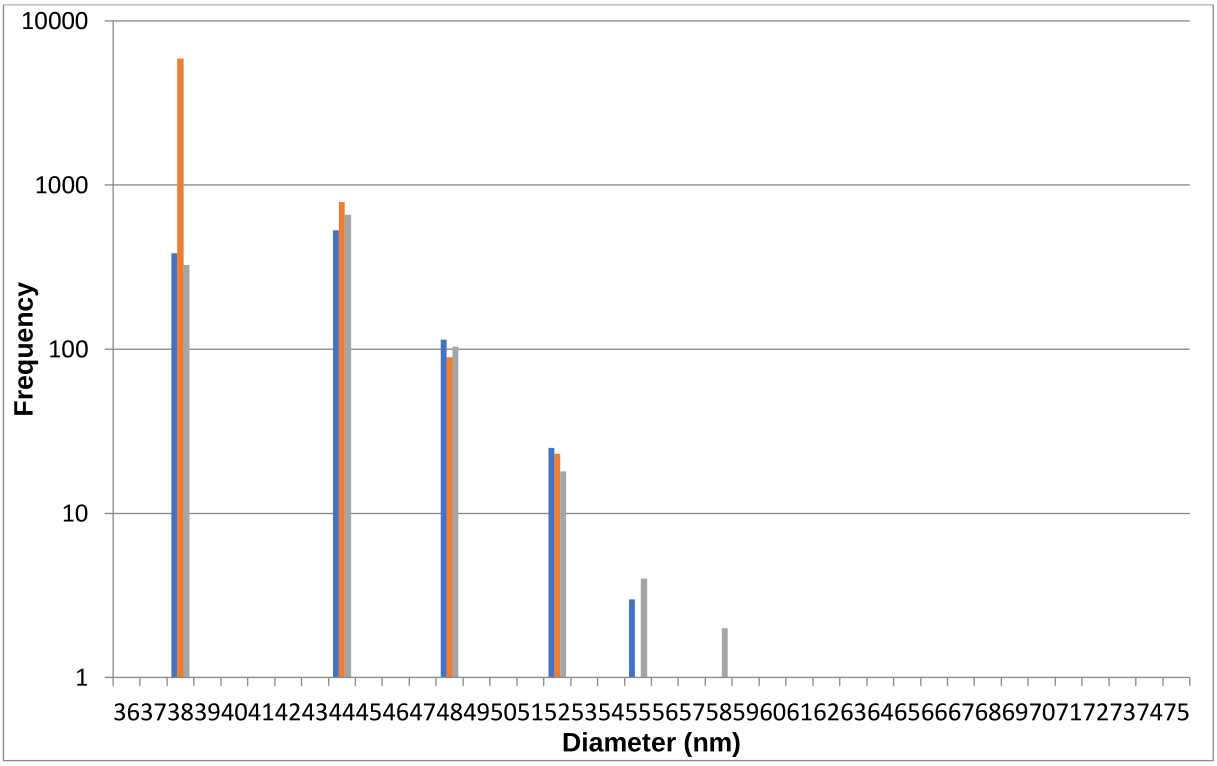

Supplementary figure S6: Size histograms for 3 runs of Basil 3 Shoot.

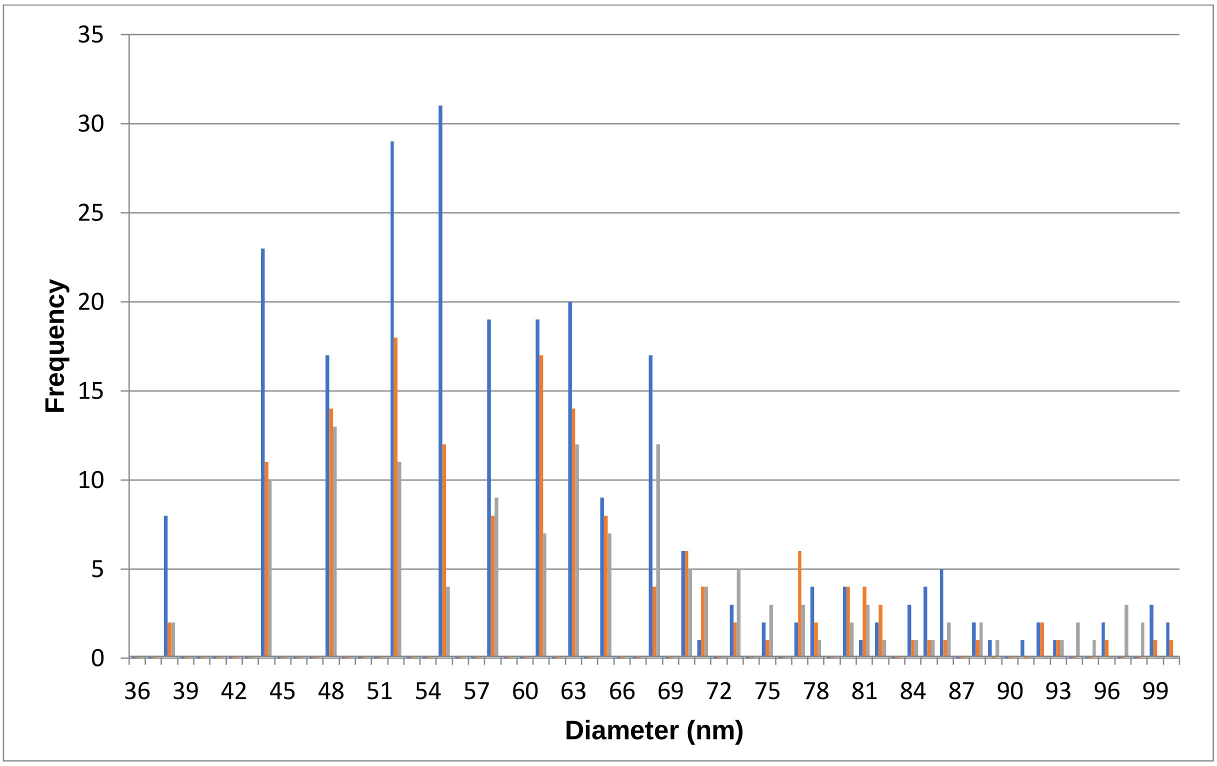

Supplementary figure S7: Size histograms for 3 runs of Dill 1 Root.

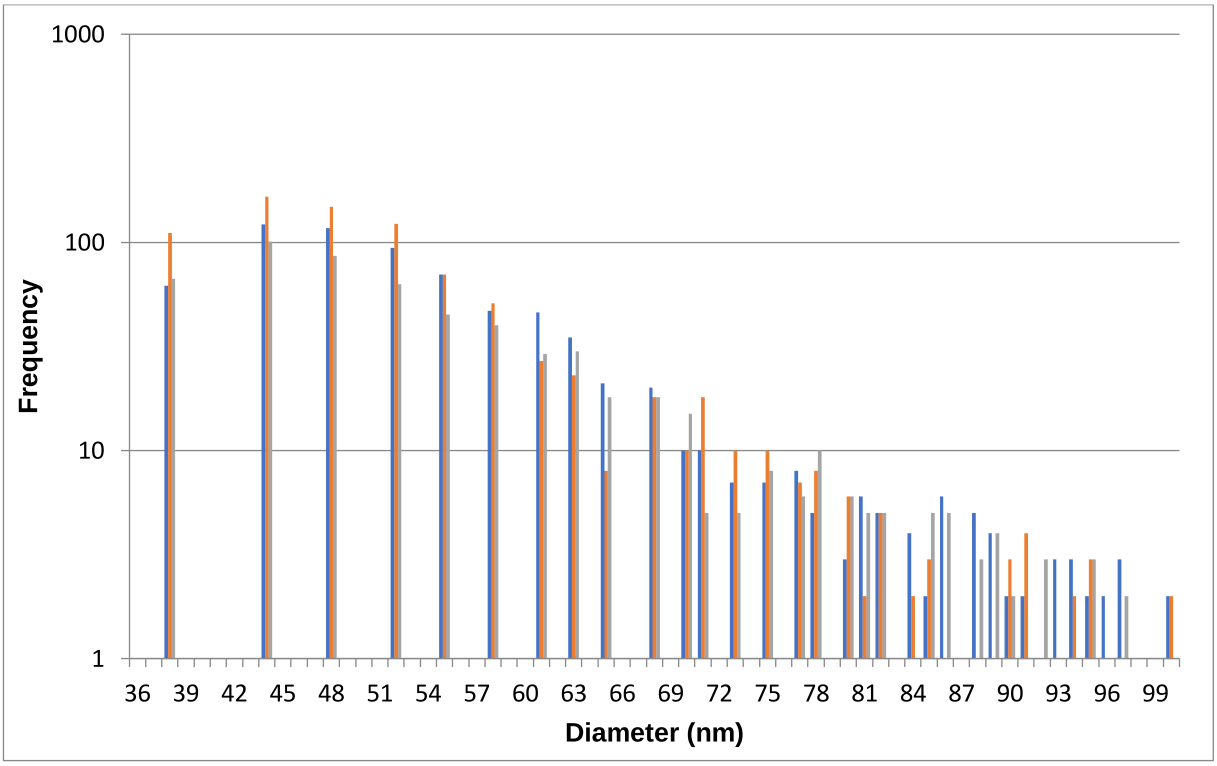

Supplementary figure S8: Size histograms for 3 runs of Dill 2 Root.

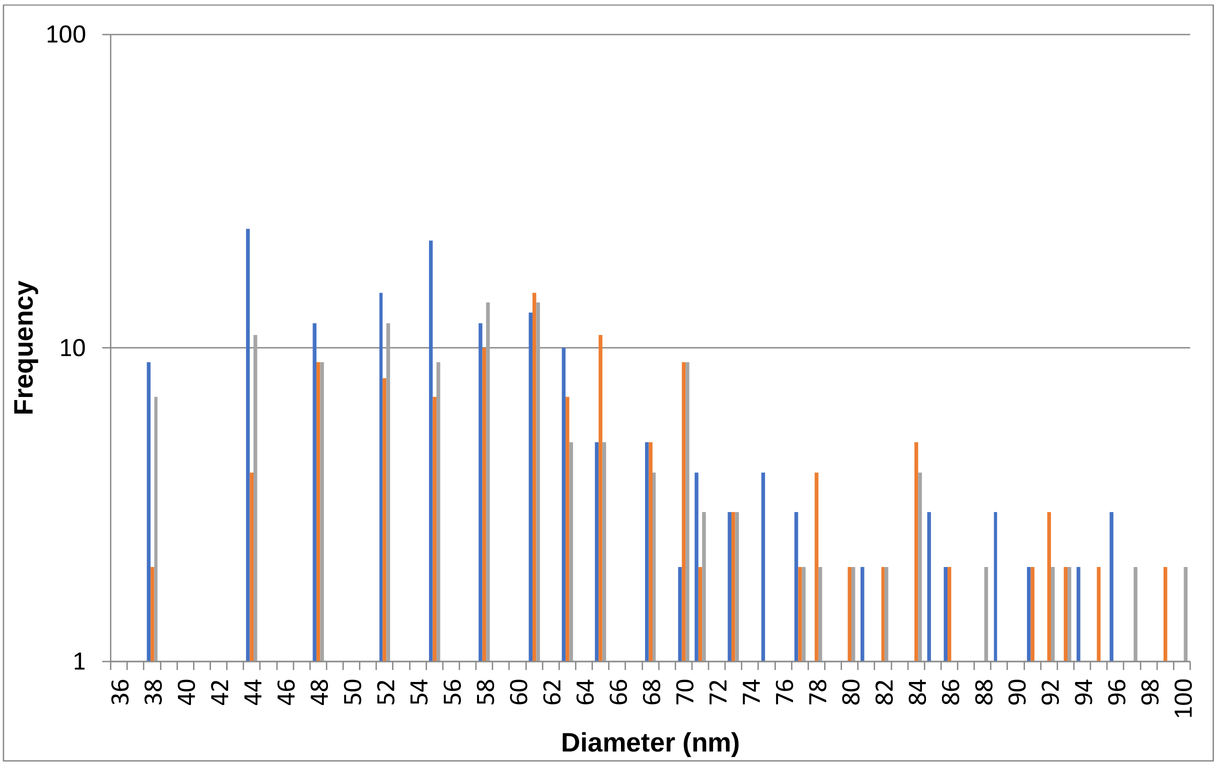

Supplementary figure S9: Size histograms for 3 runs of Dill 3 Root.

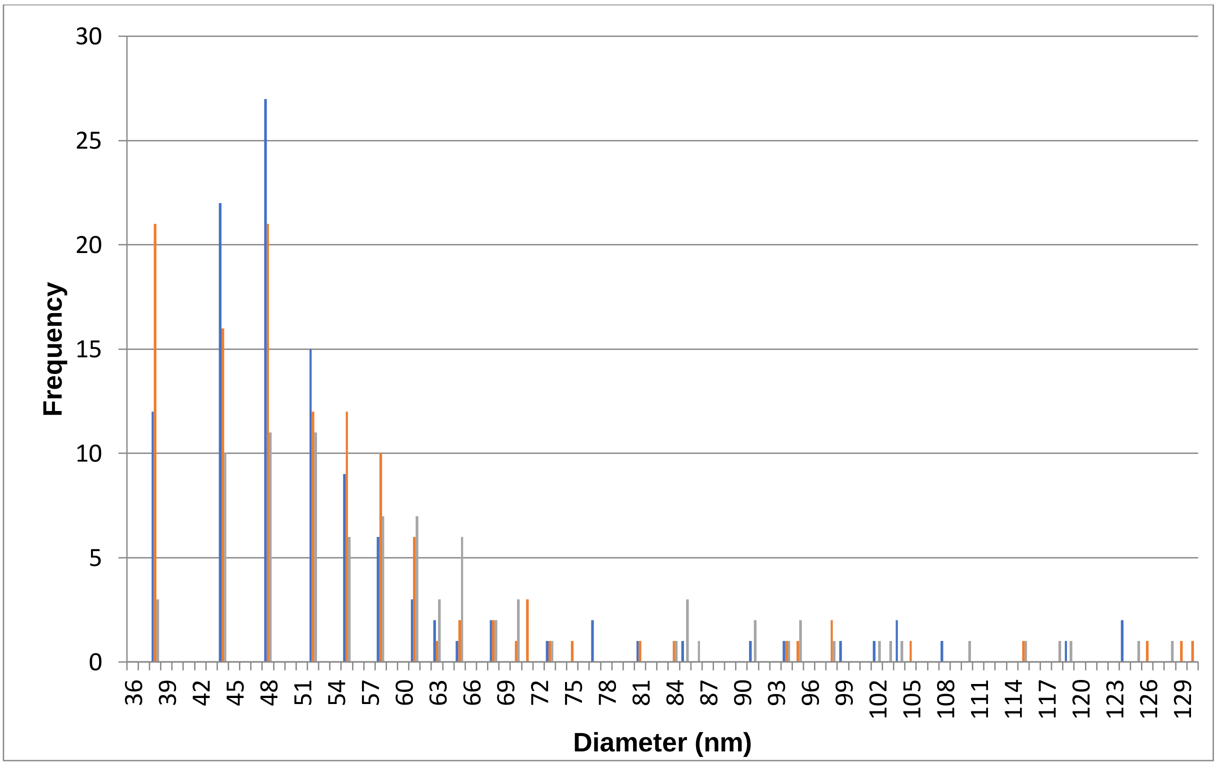

Supplementary figure S10: Size histograms for 3 runs of Dill 1 Shoot.

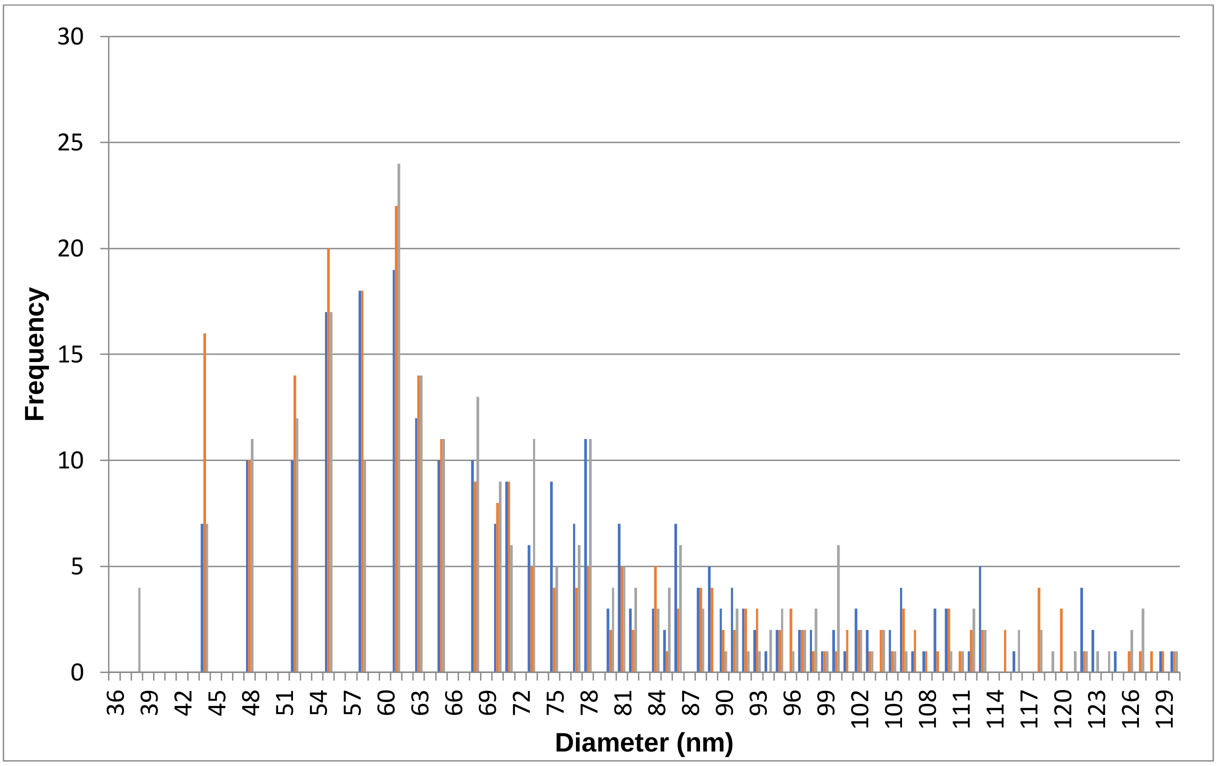

Supplementary figure S11: Size histograms for 3 runs of Dill 2 Shoot.

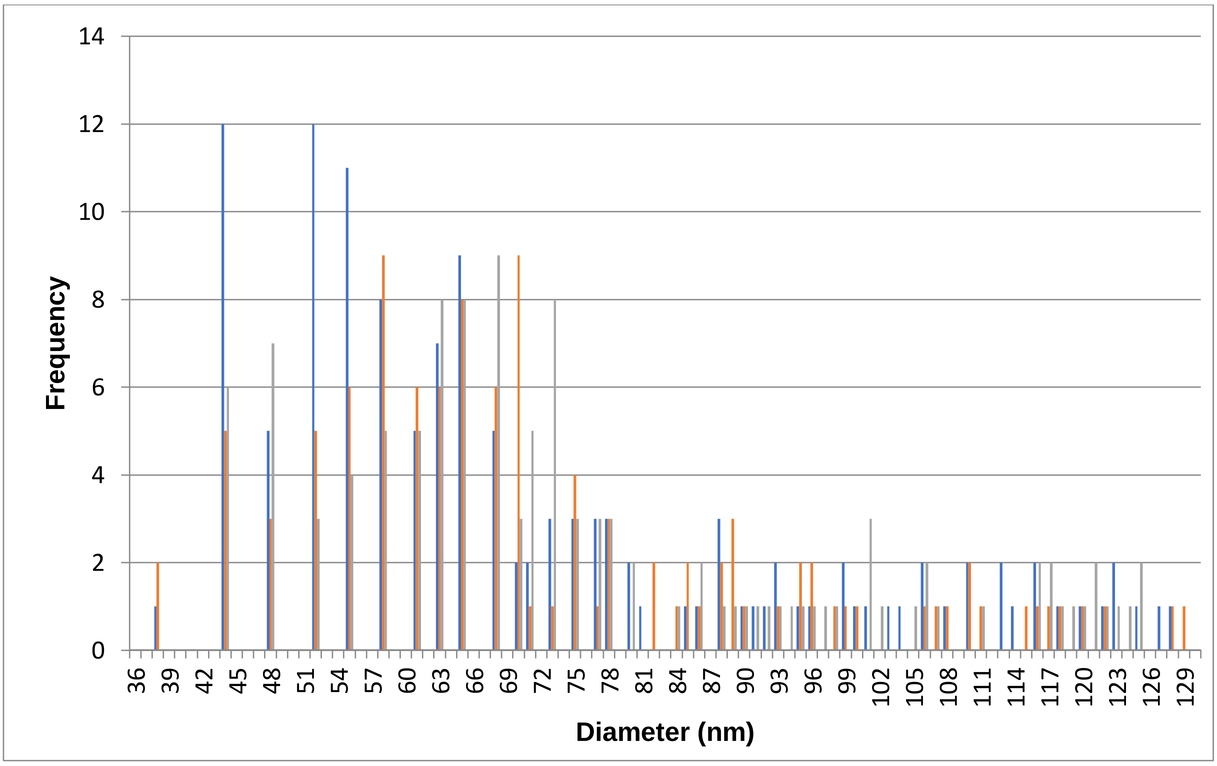

Supplementary figure S12: Size histograms for 3 runs of Dill 3 Shoot.

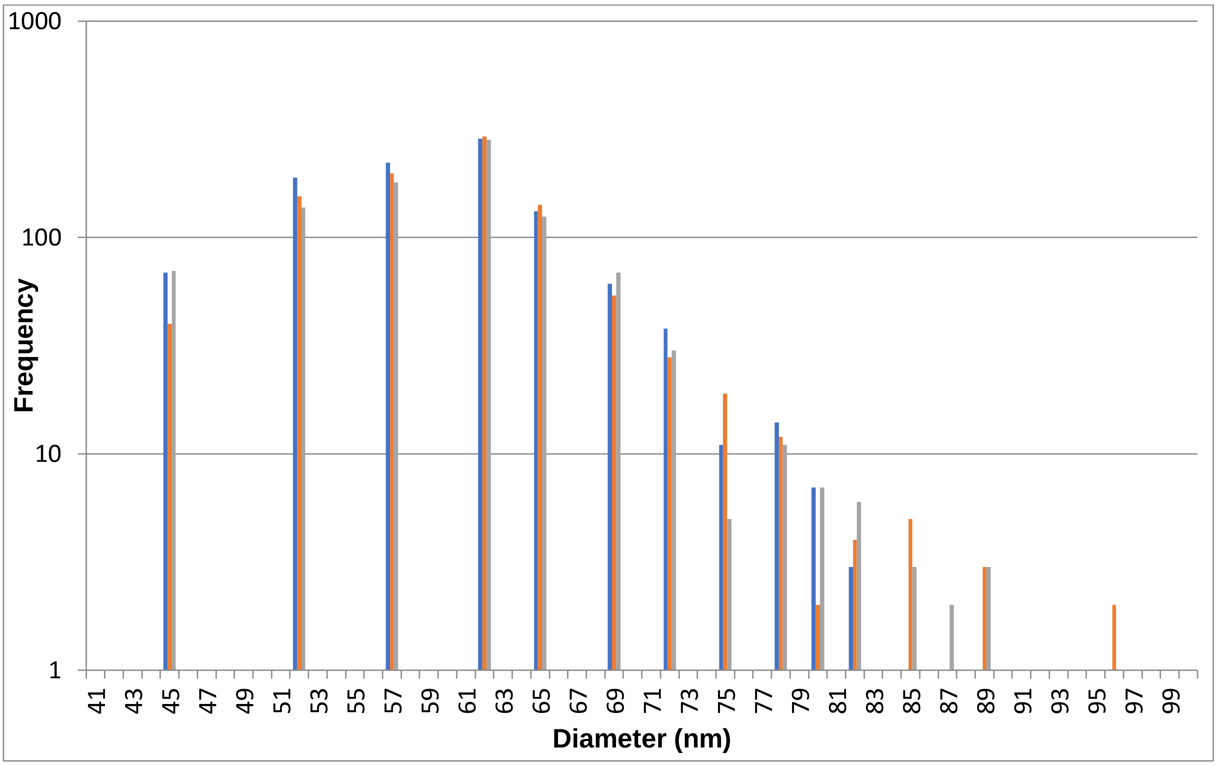

Supplementary figure S13: Size histograms for 3 runs of Chard 1 Root.

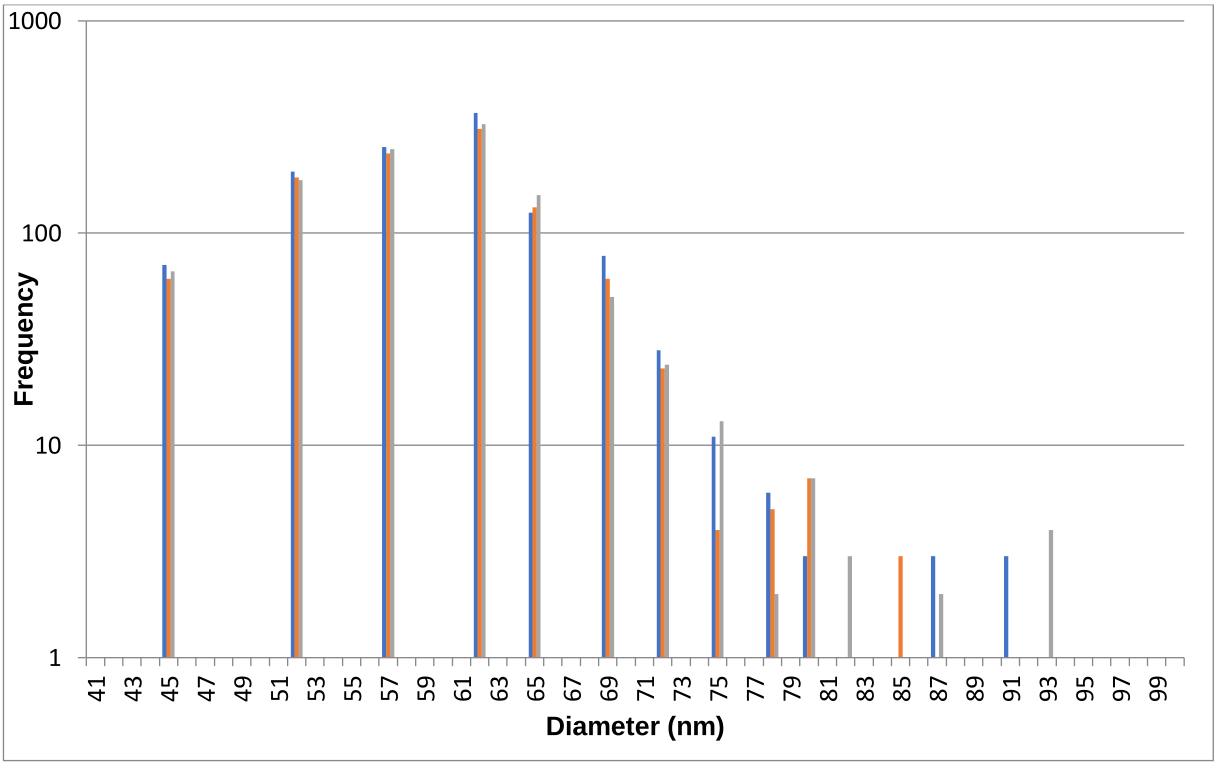

Supplementary figure S14: Size histograms for 3 runs of Chard 2 Root.

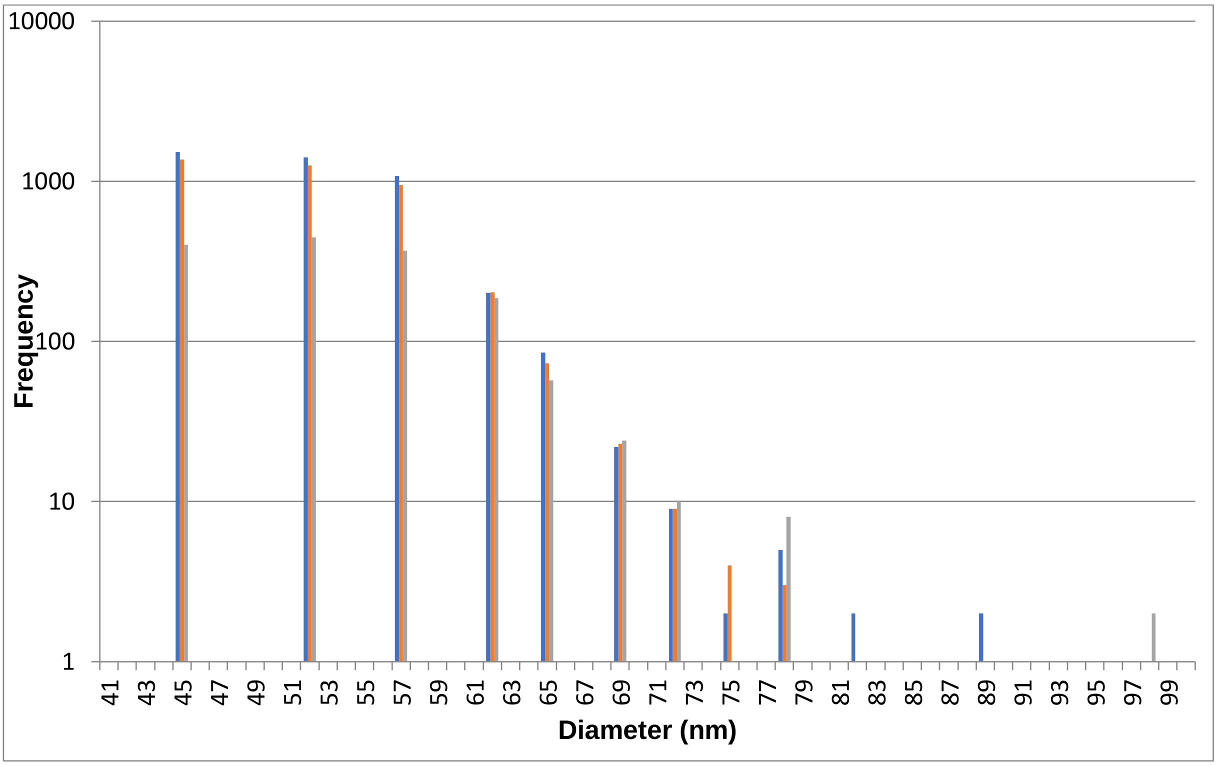

Supplementary figure S15: Size histograms for 3 runs of Chard 3 Root.

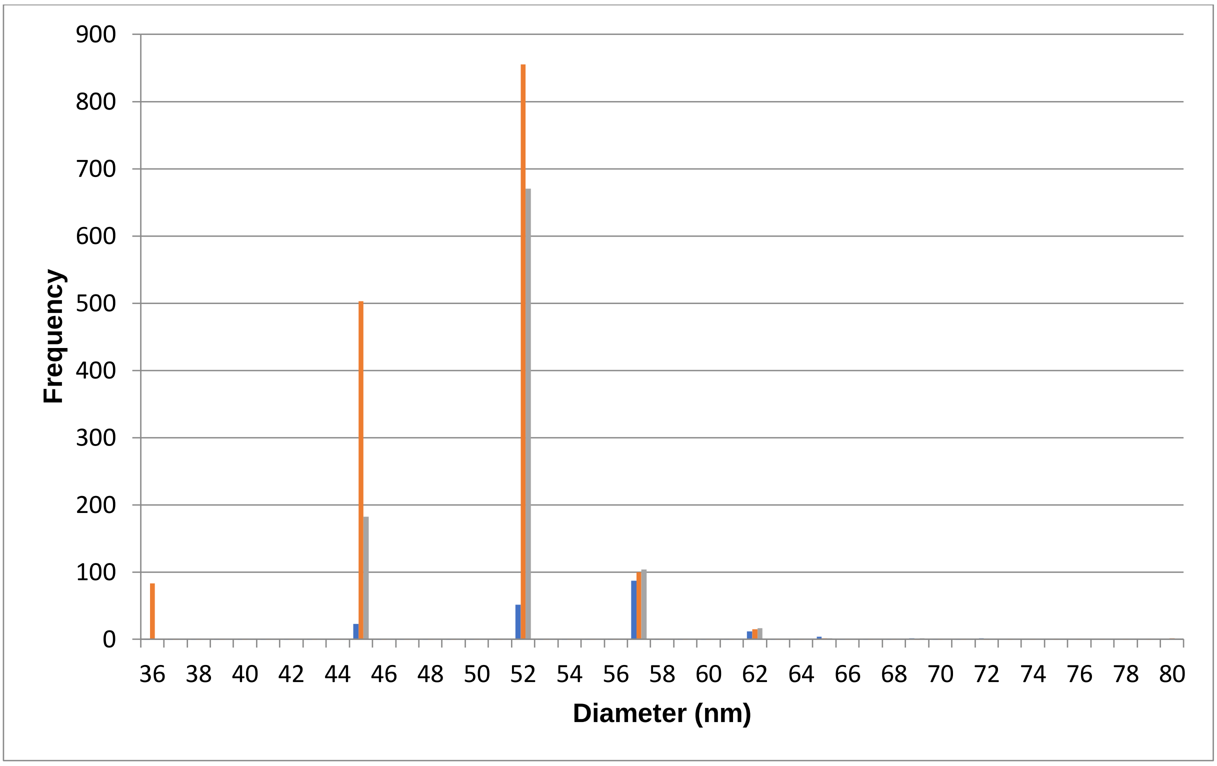

Supplementary figure S16: Size histograms for 3 runs of Chard 1 Shoot.

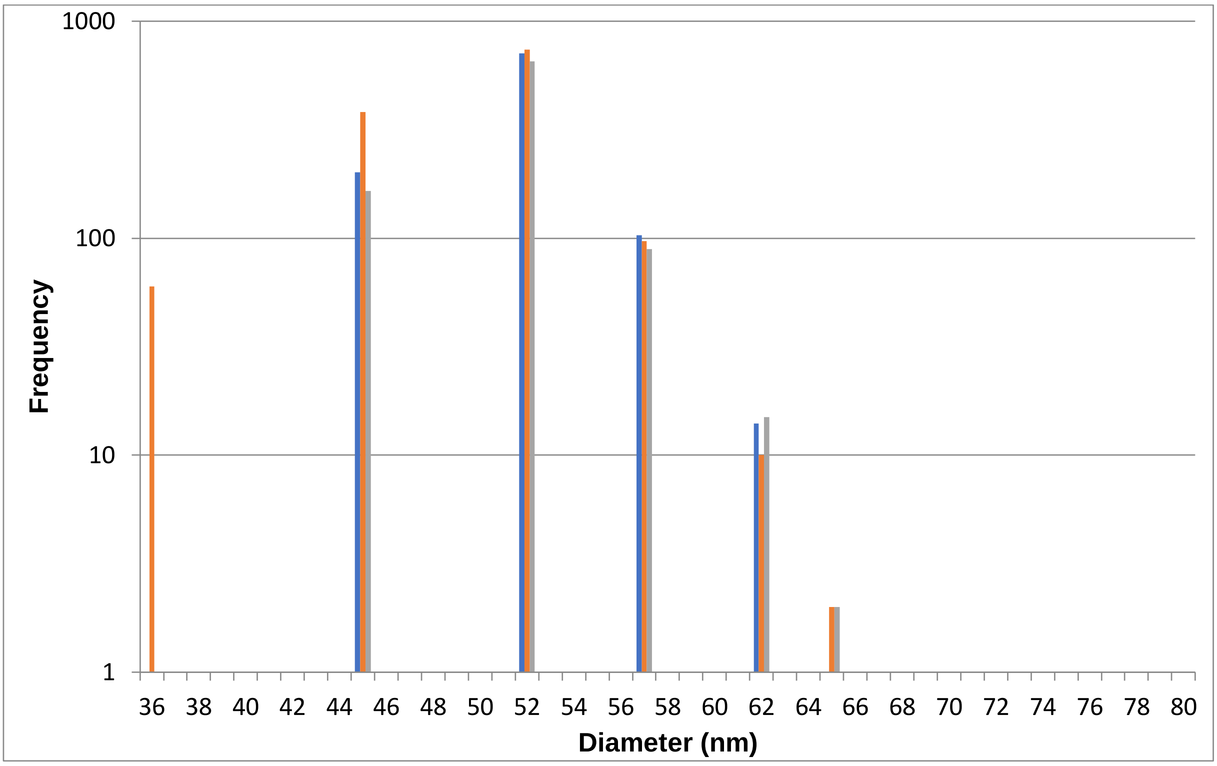

Supplementary figure S17: Size histograms for 3 runs of Chard 2 Shoot.

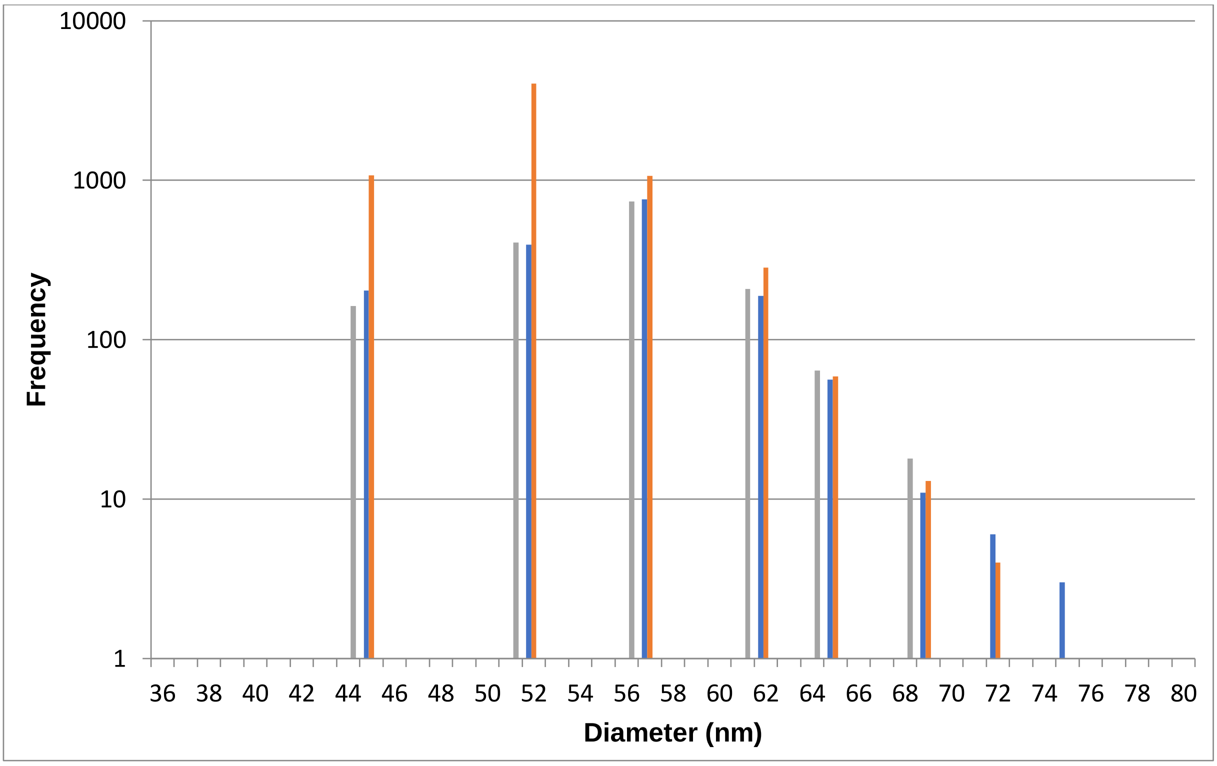

Supplementary figure S18: Size histograms for 3 runs of Chard 3 Shoot.

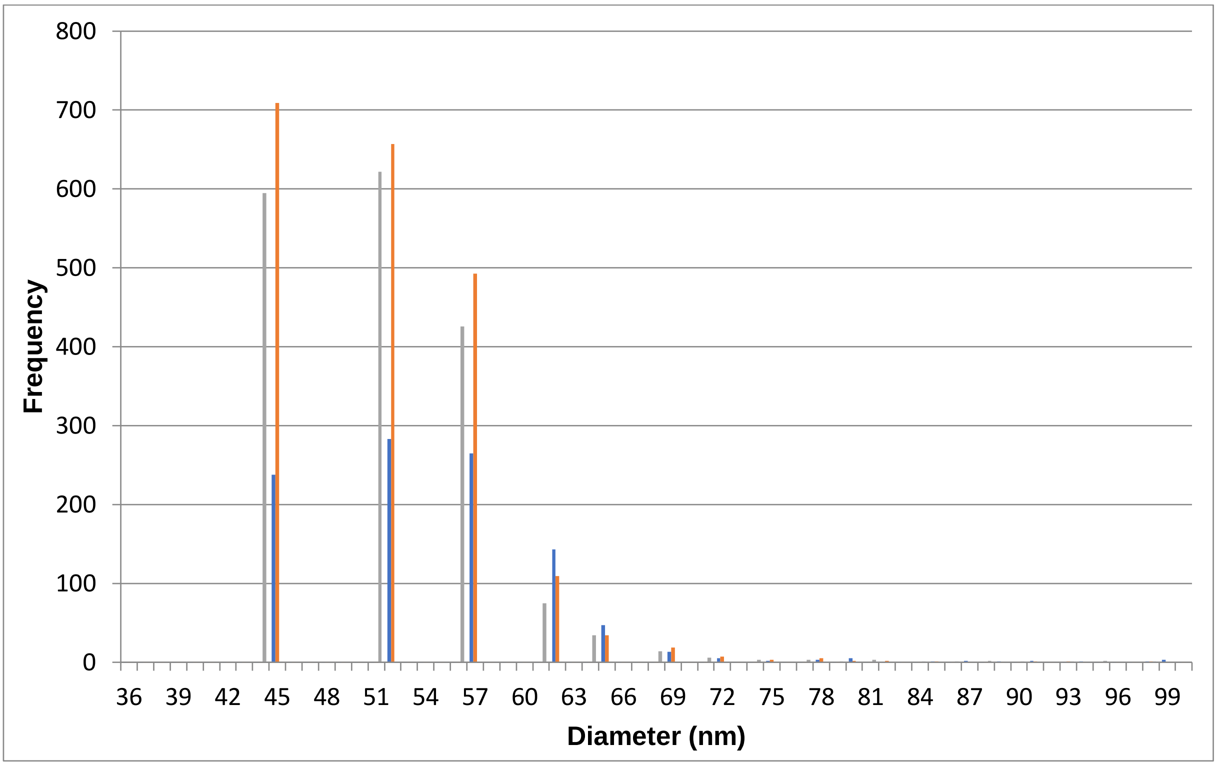

Supplementary figure S19: Size histograms for 3 runs of Spinach 1 Root.

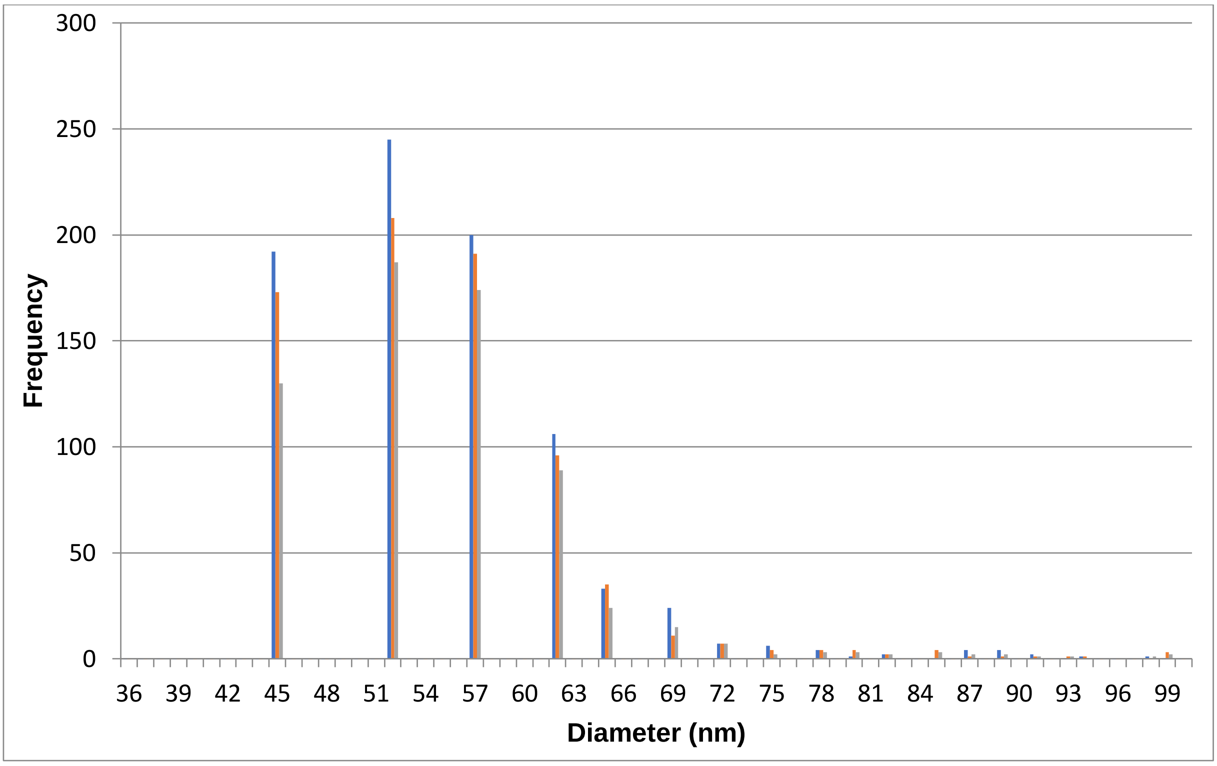

Supplementary figure S20: Size histograms for 3 runs of Spinach 2 Root.

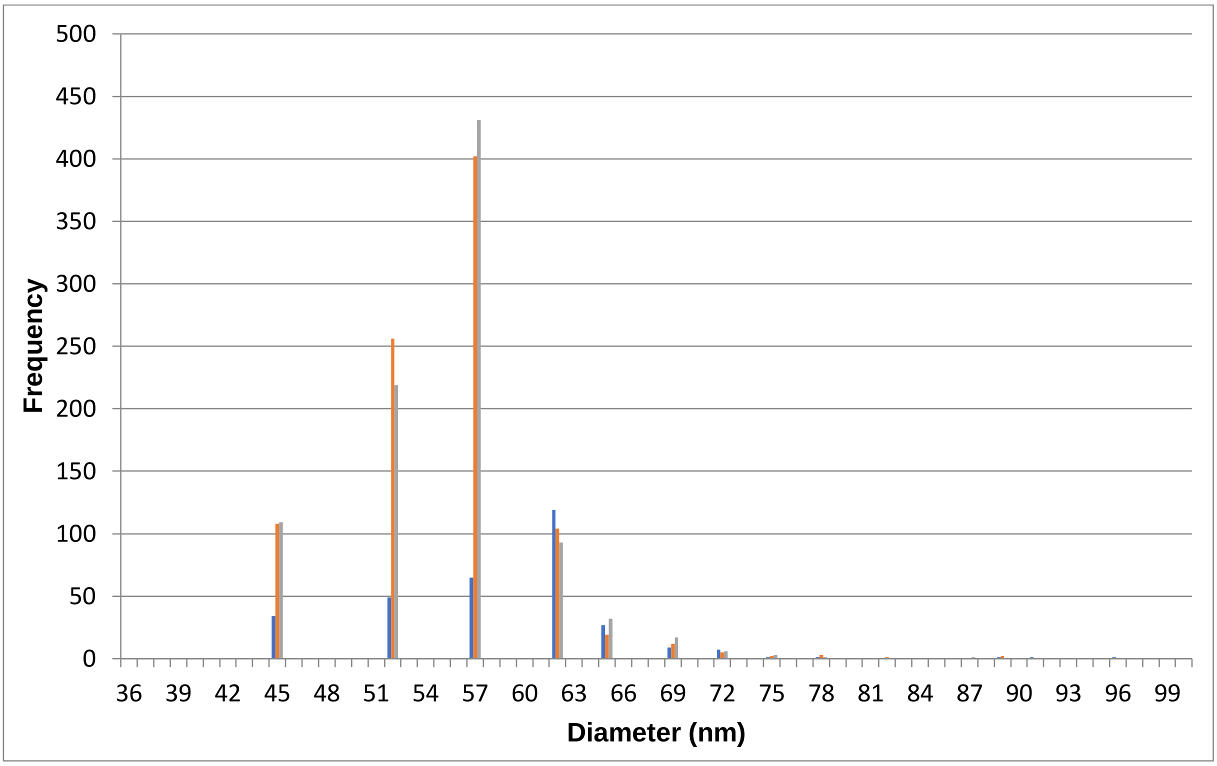

Supplementary figure S21: Size histograms for 3 runs of Spinach 3 Root.

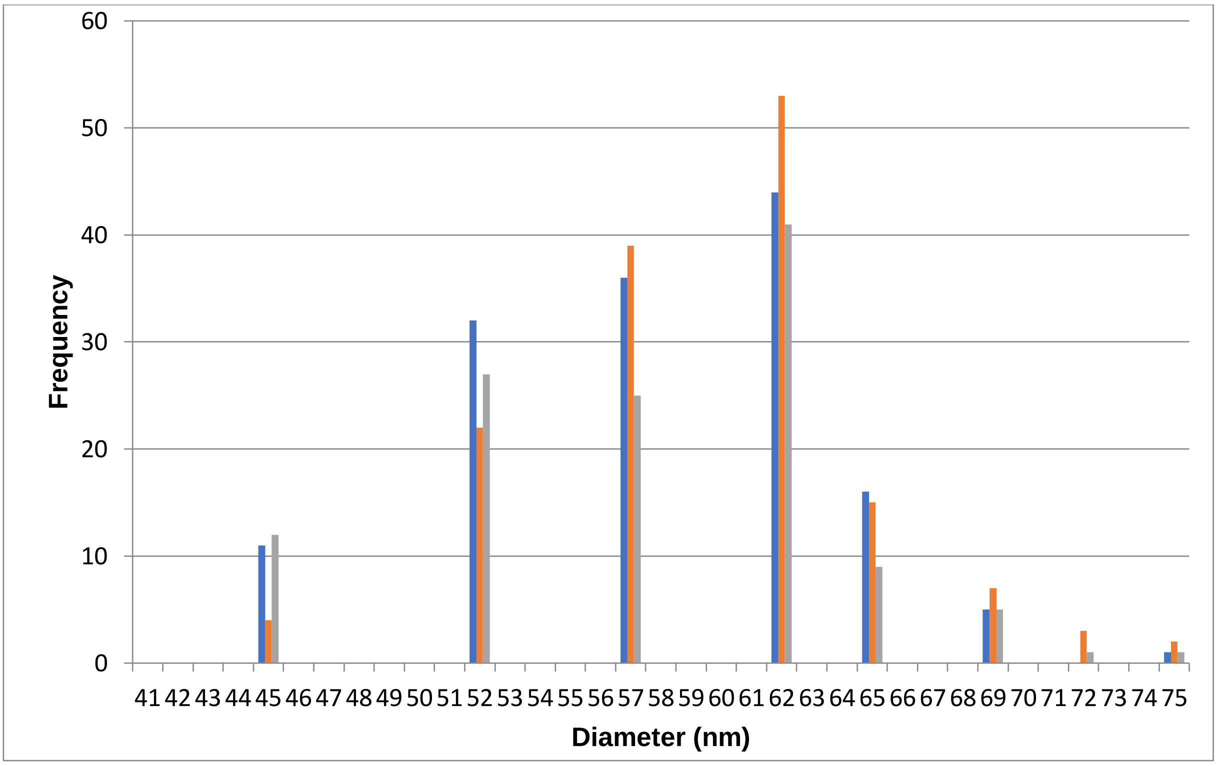

Supplementary figure S22: Size histograms for 3 runs of Spinach 1 Shoot.

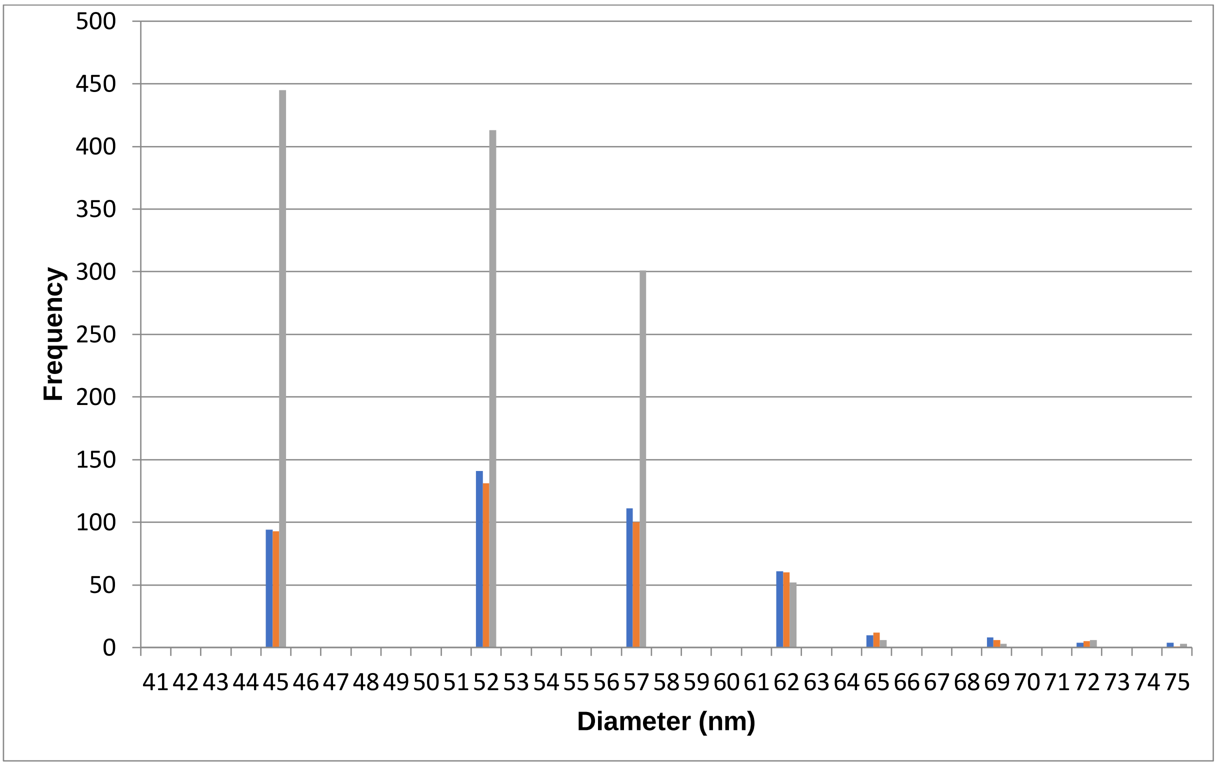

Supplementary figure S23: Size histograms for 3 runs of Spinach 2 Shoot.

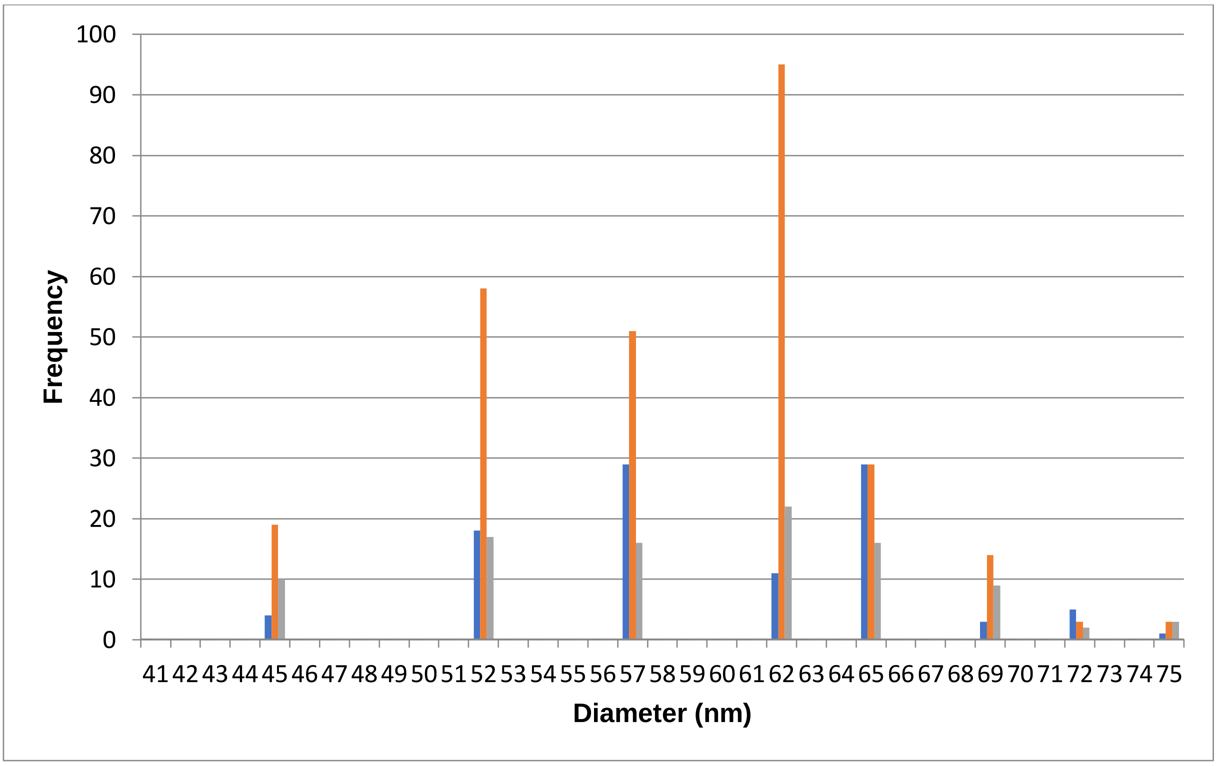

Supplementary figure S24: Size histograms for 3 runs of Spinach 3 Shoot.

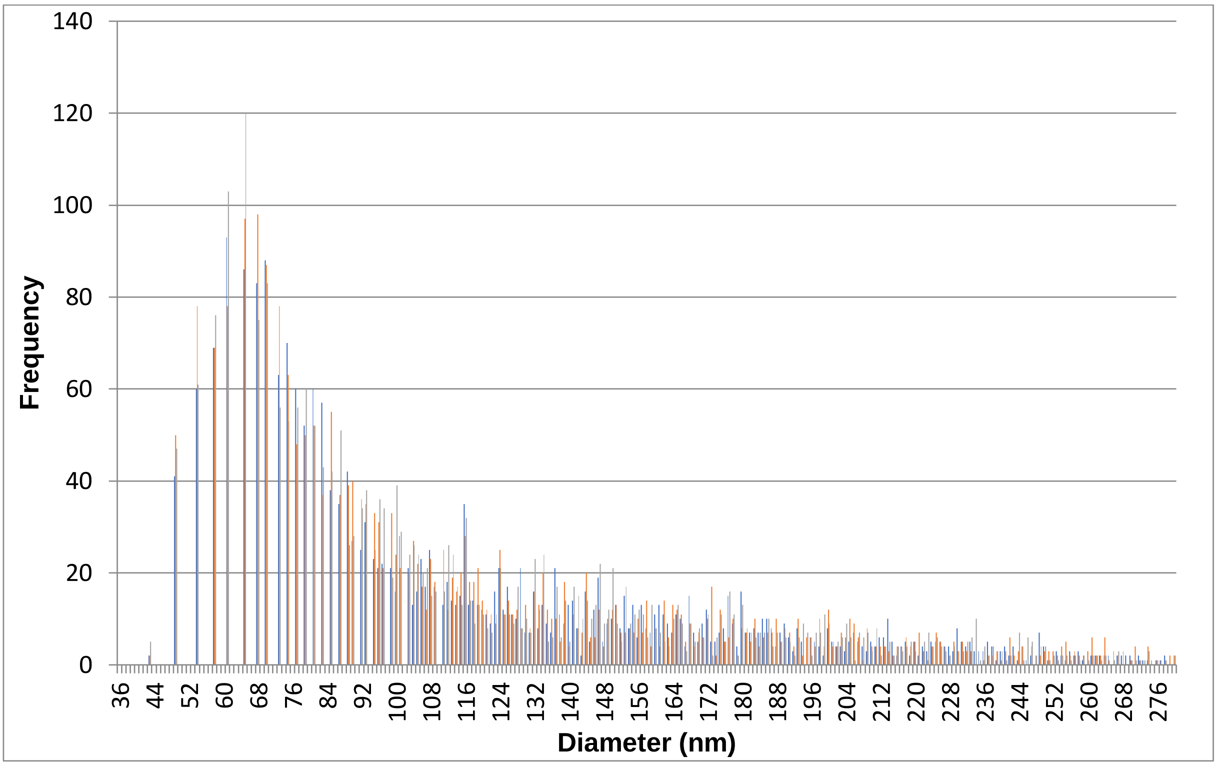

Supplementary figure S25: Size histograms for 3 runs of Brussels Sprout 1 Root.

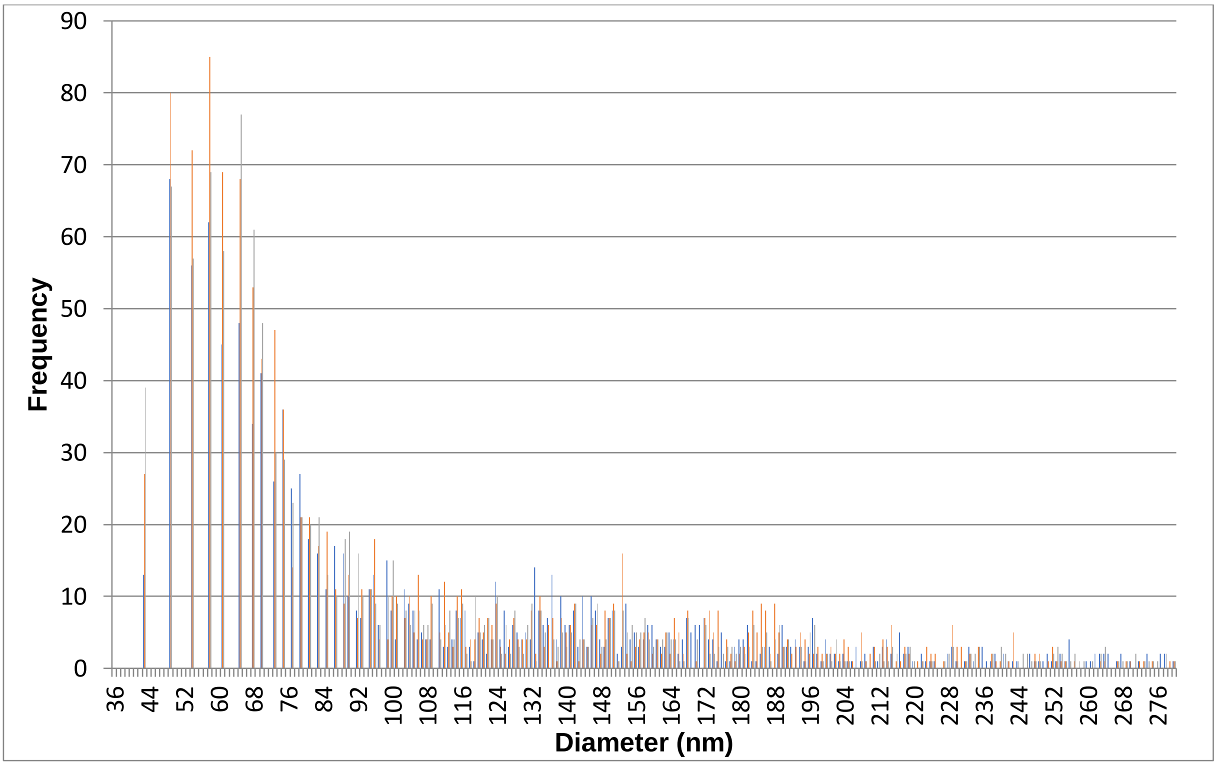

Supplementary figure S26: Size histograms for 3 runs of Brussels Sprout 2 Root.

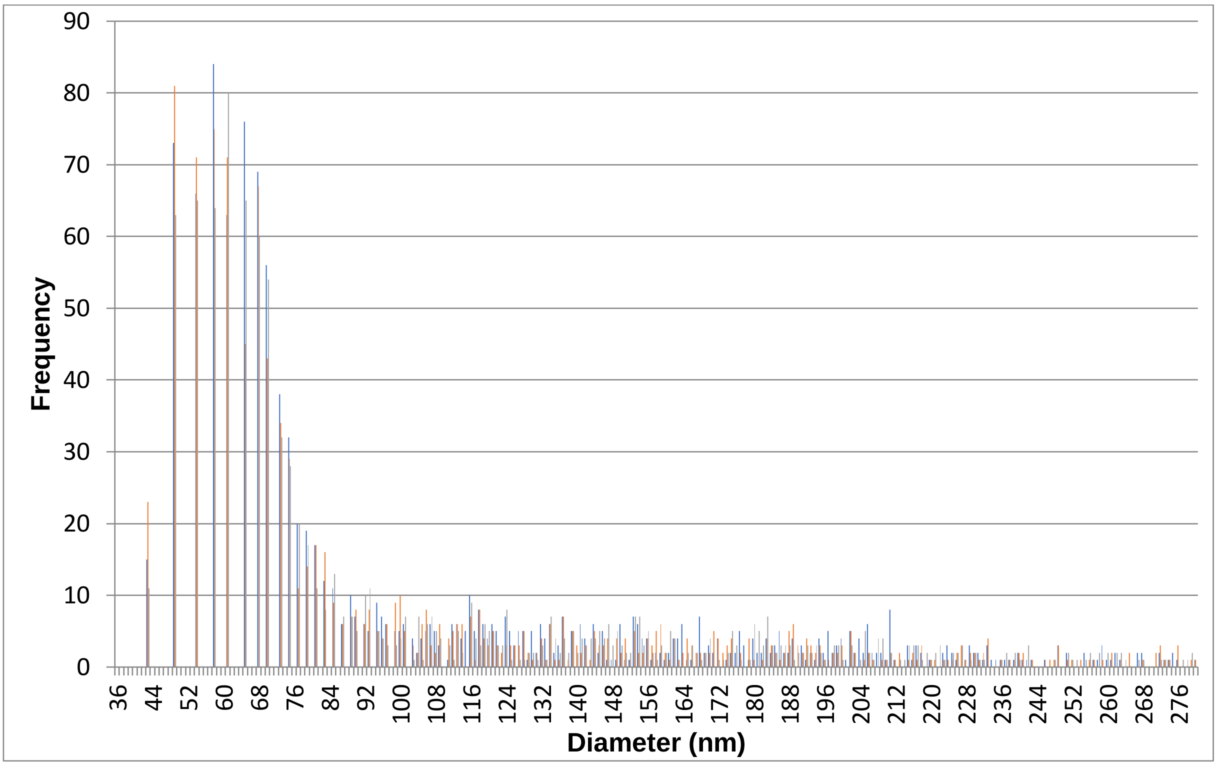

Supplementary figure S27: Size histograms for 3 runs of Brussels Sprout 3 Root.

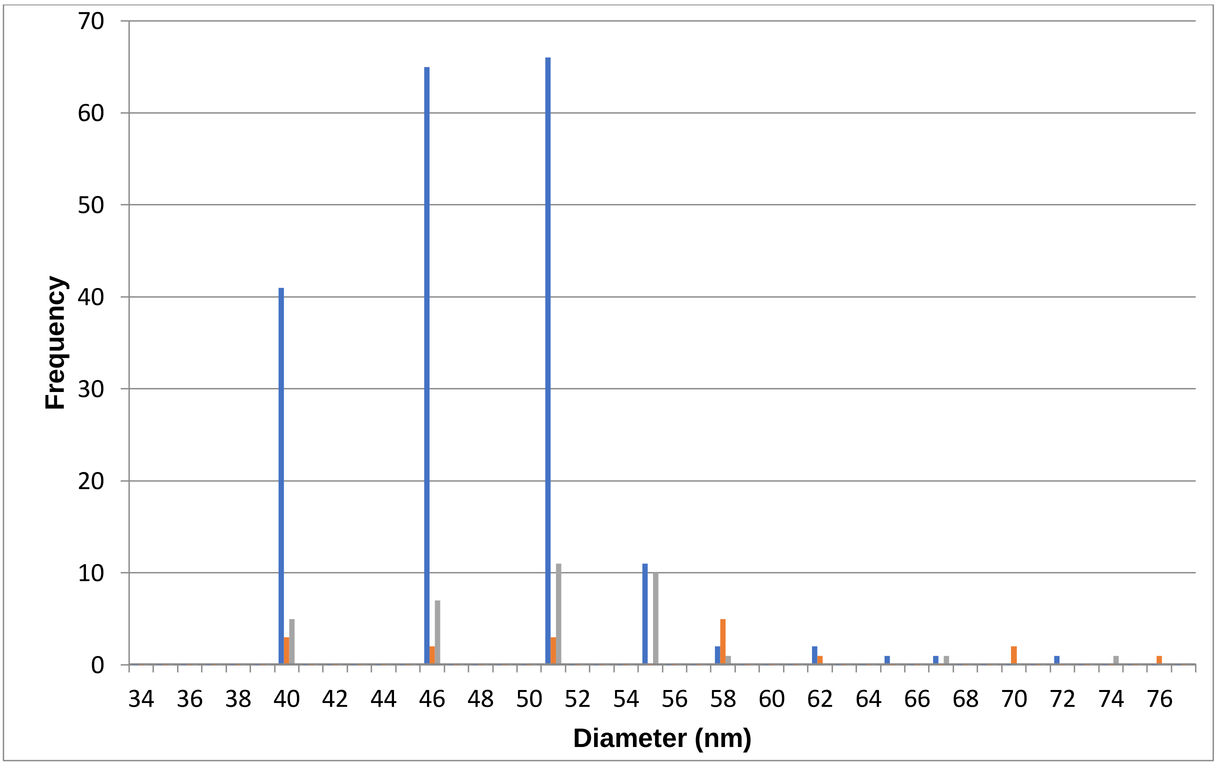

Supplementary figure S28: Size histograms for 3 runs of Brussels Sprout 1 Shoot.

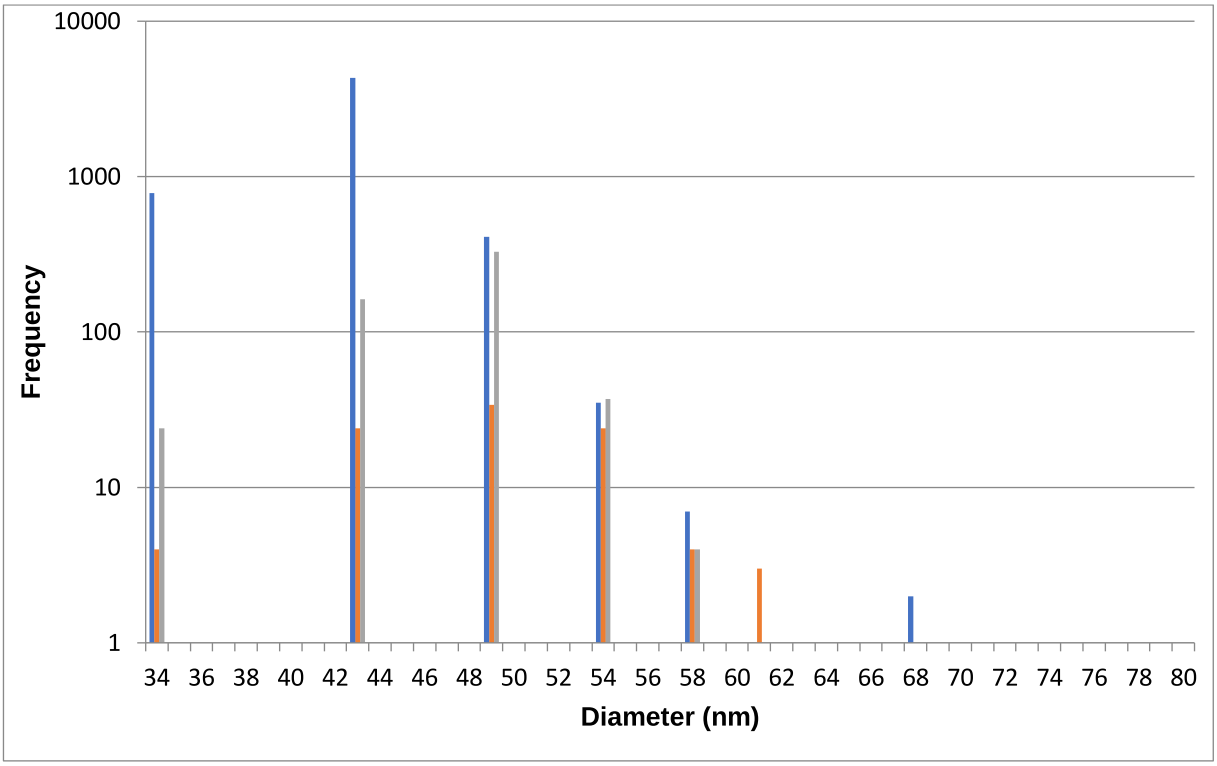

Supplementary figure S29: Size histograms for 3 runs of Brussels Sprout 2 Shoot.

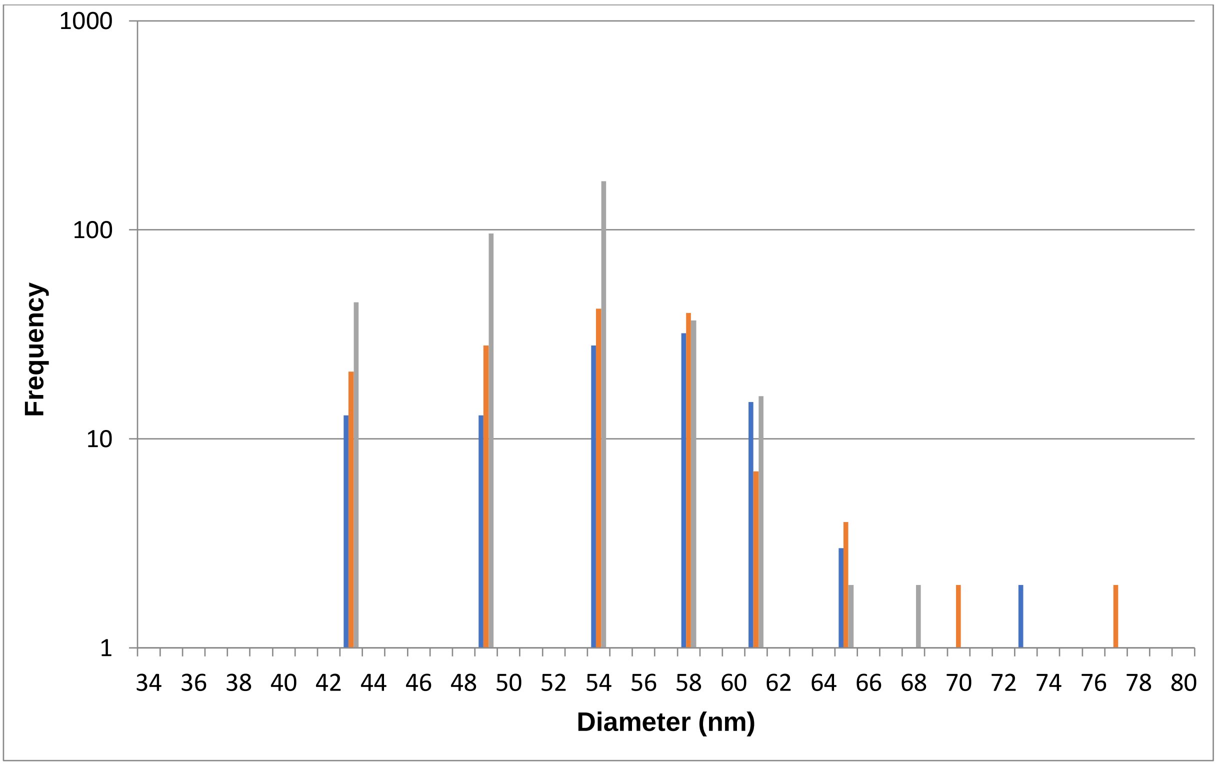

Supplementary figure S30: Size histograms for 3 runs of Brussels Sprout 3 Shoot.

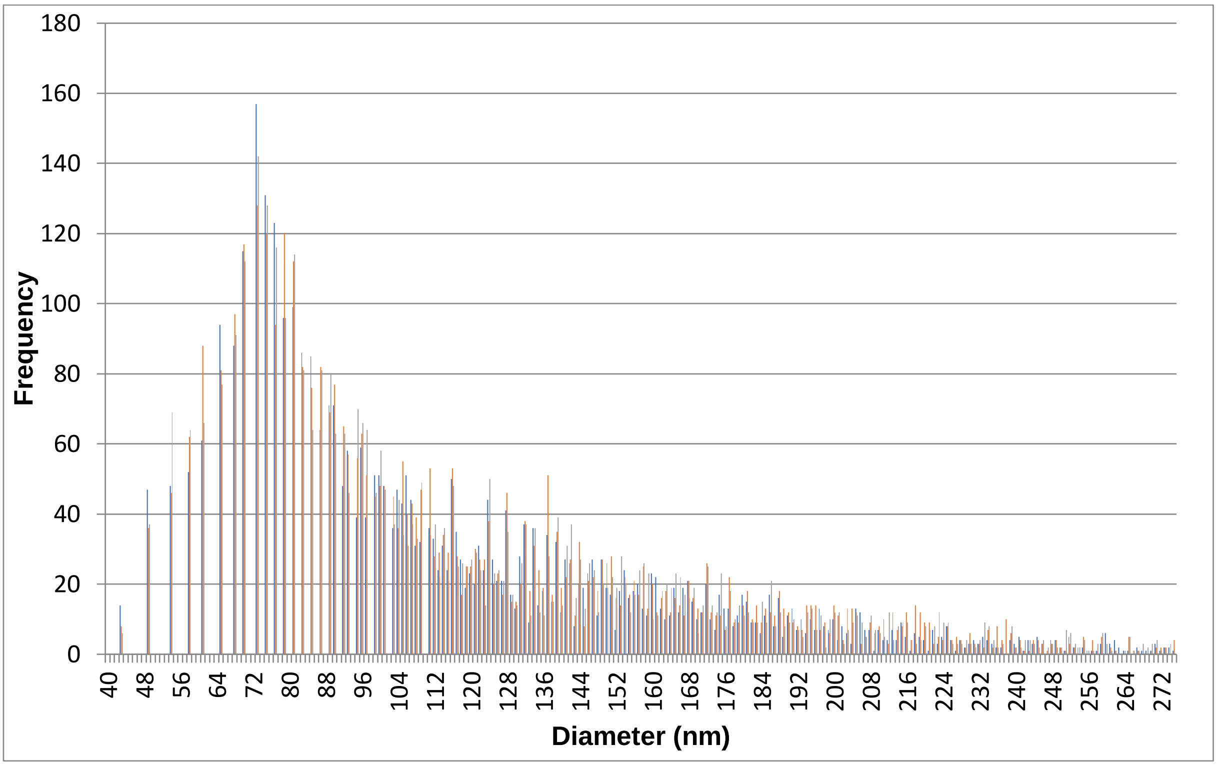

Supplementary figure S31: Size histograms for 3 runs of Broccoli 1 Root.

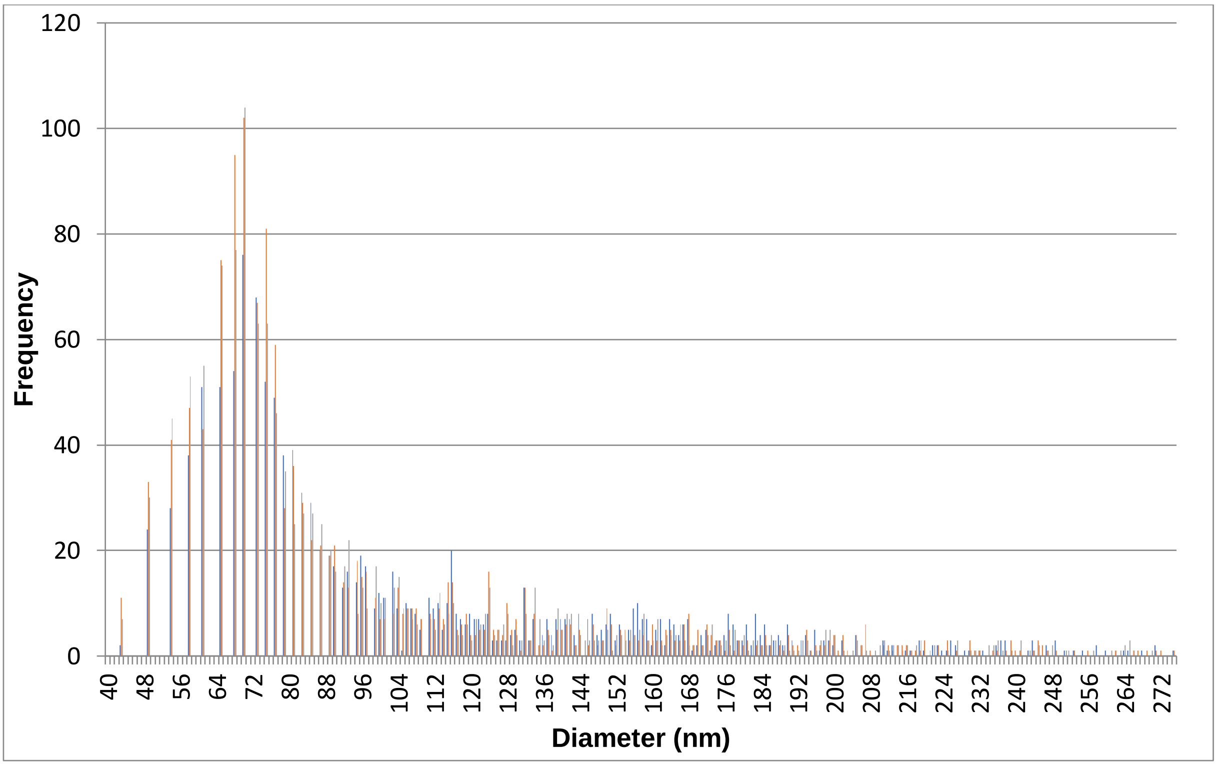

Supplementary figure S32: Size histograms for 3 runs of Broccoli 2 Root.

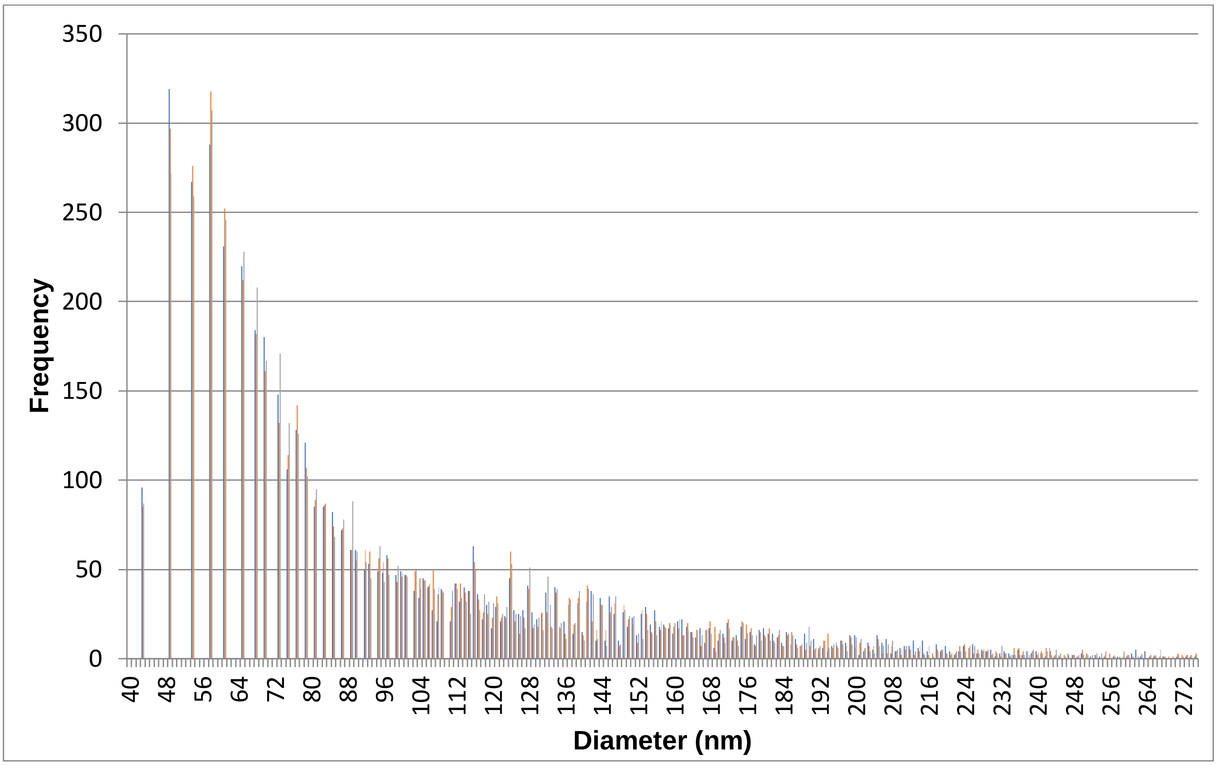

Supplementary figure S33: Size histograms for 3 runs of Broccoli 3 Root.

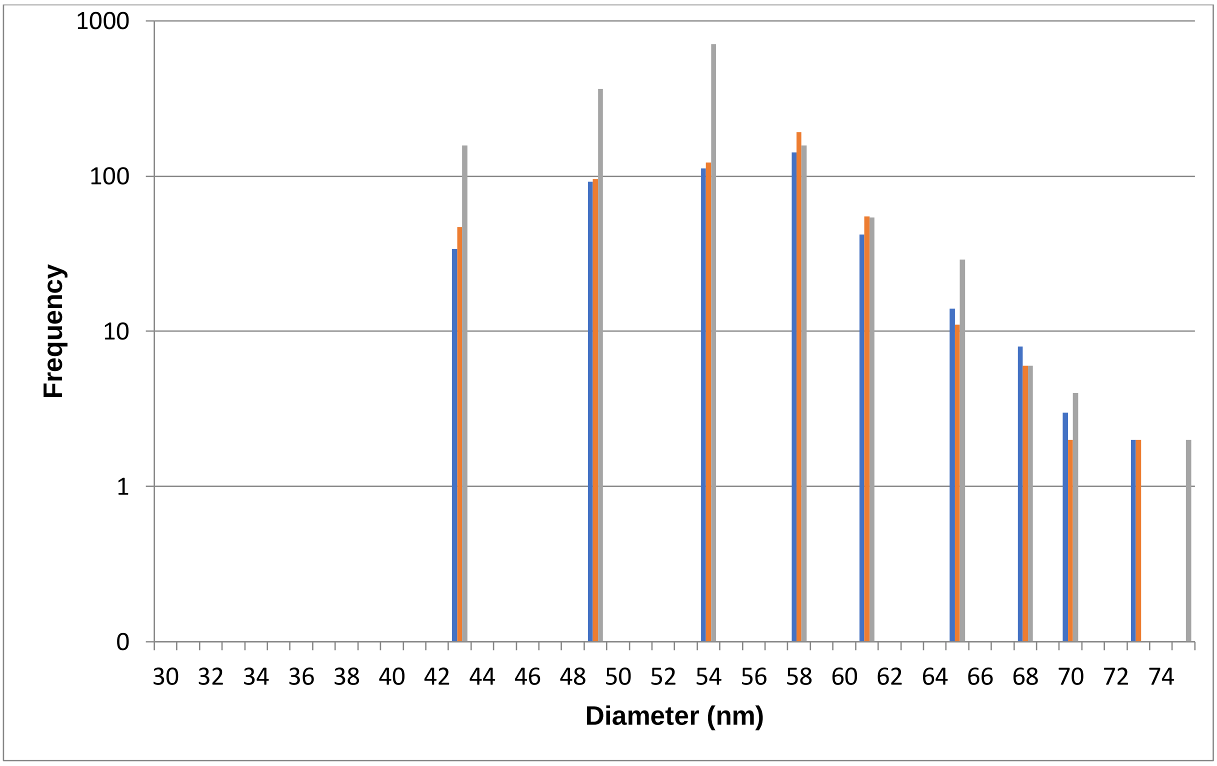

Supplementary figure S34: Size histograms for 3 runs of Broccoli 1 Shoot.

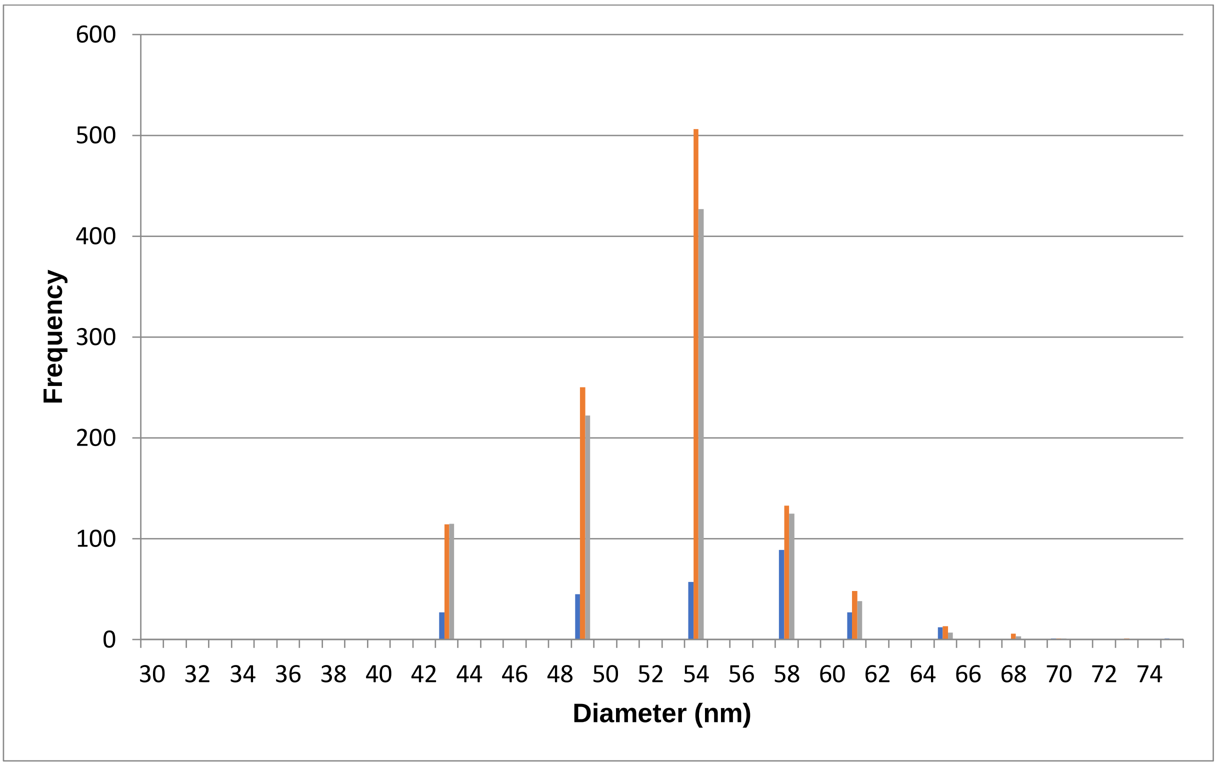

Supplementary figure S35: Size histograms for 3 runs of Broccoli 2 Shoot.

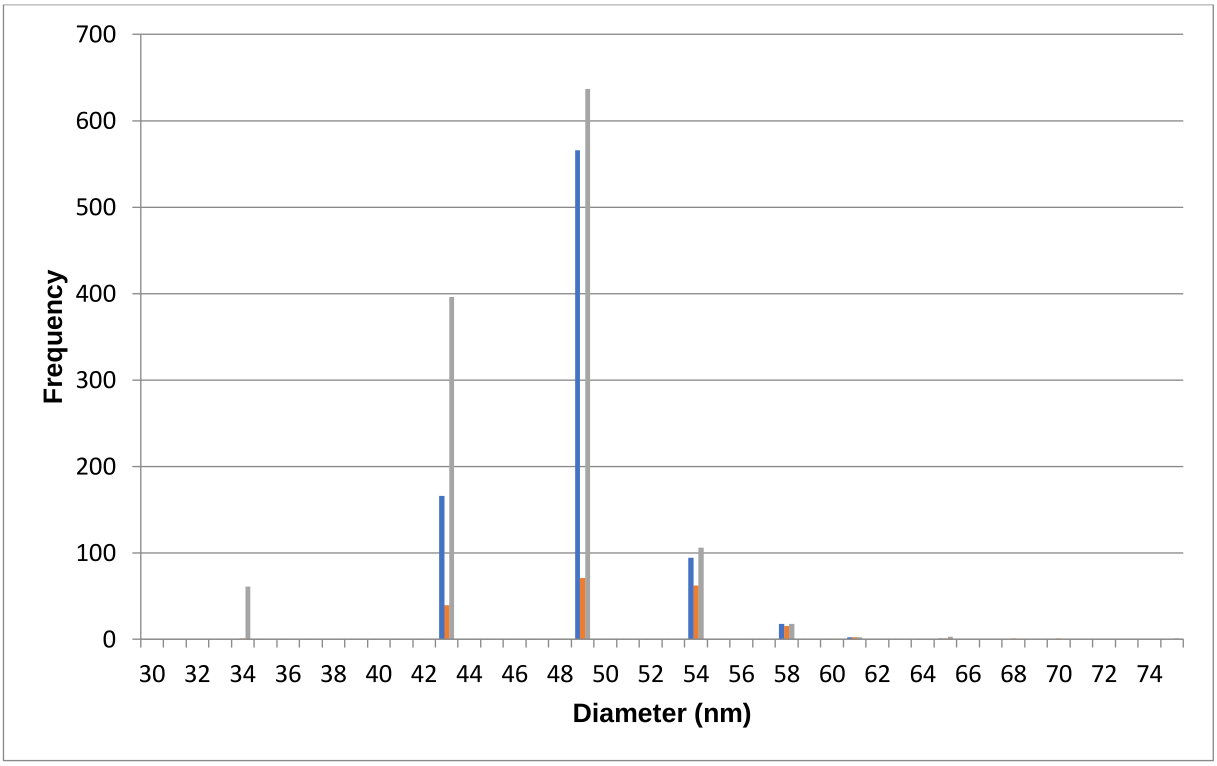

Supplementary figure S36: Size histograms for 3 runs of Broccoli 3 Shoot.

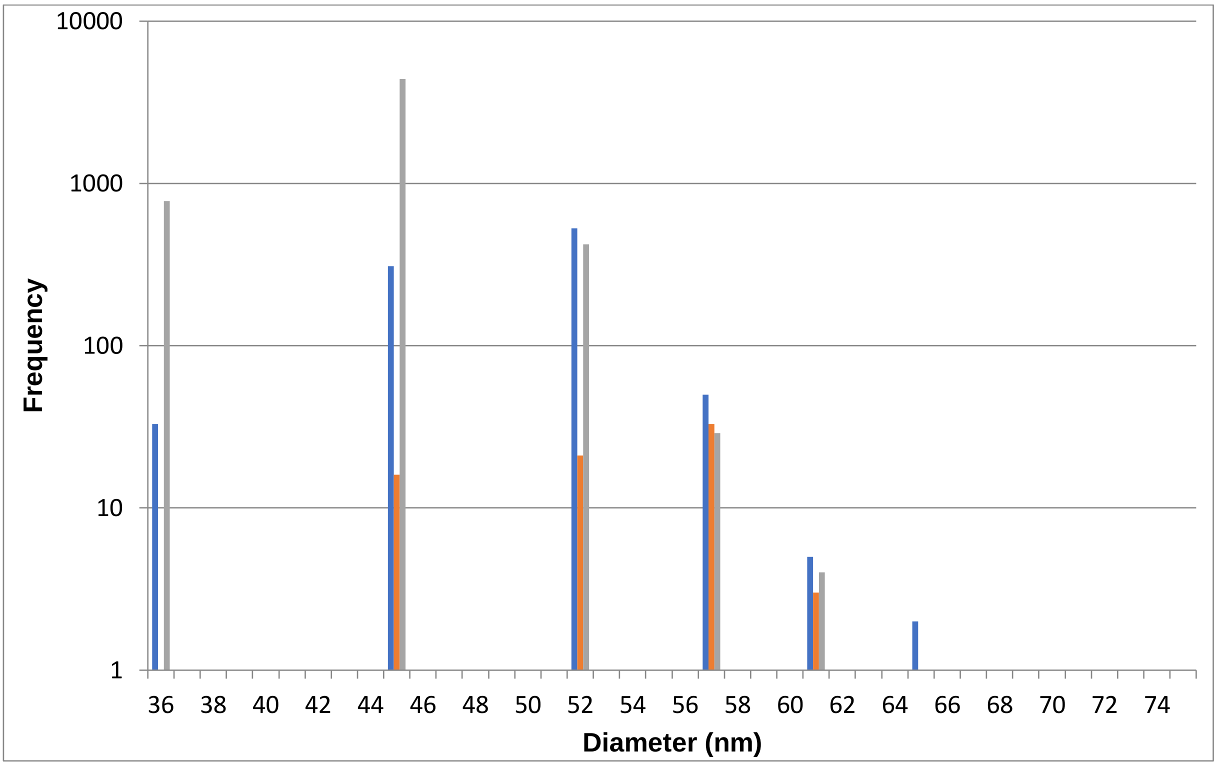

Supplementary figure S37: Size histograms for 3 runs of Lambs lettuce 1 Root.

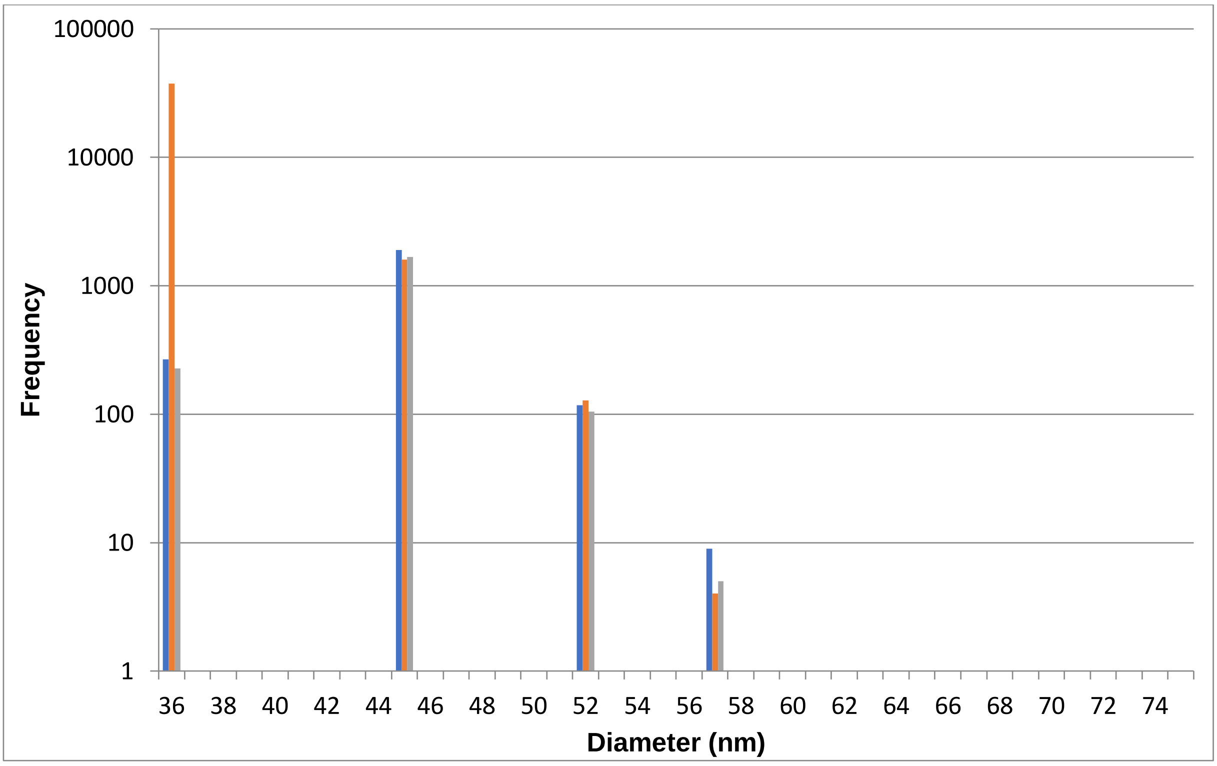

Supplementary figure S38: Size histograms for 3 runs of Lambs lettuce 2 Root.

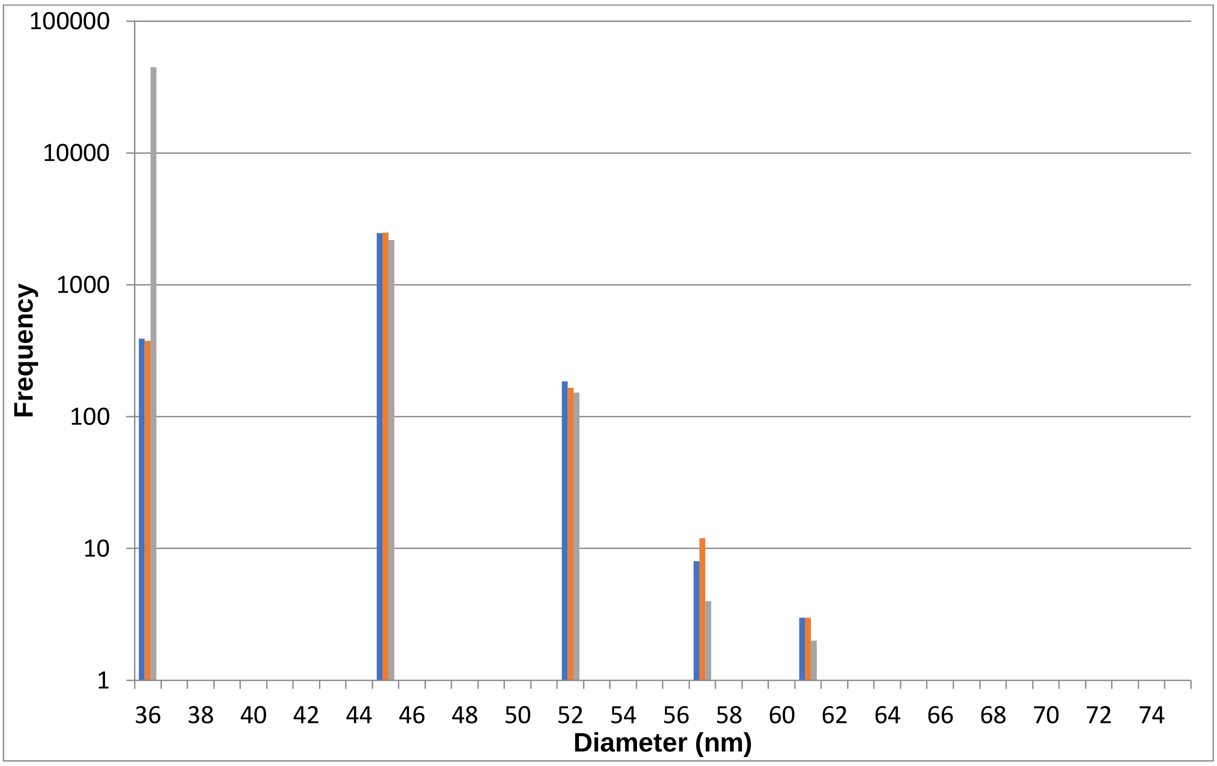

Supplementary figure S39: Size histograms for 3 runs of Lambs lettuce 3 Root.

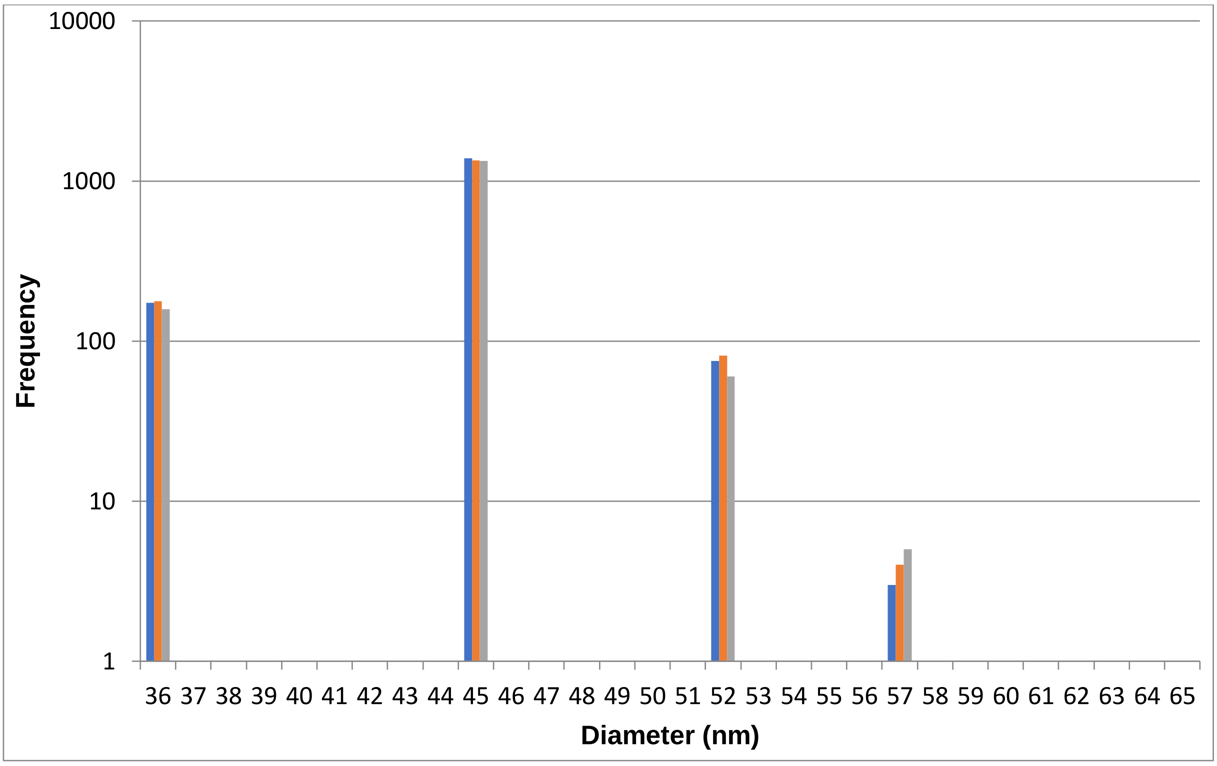

Supplementary figure S40: Size histograms for 3 runs of Lambs lettuce 1 Shoot.

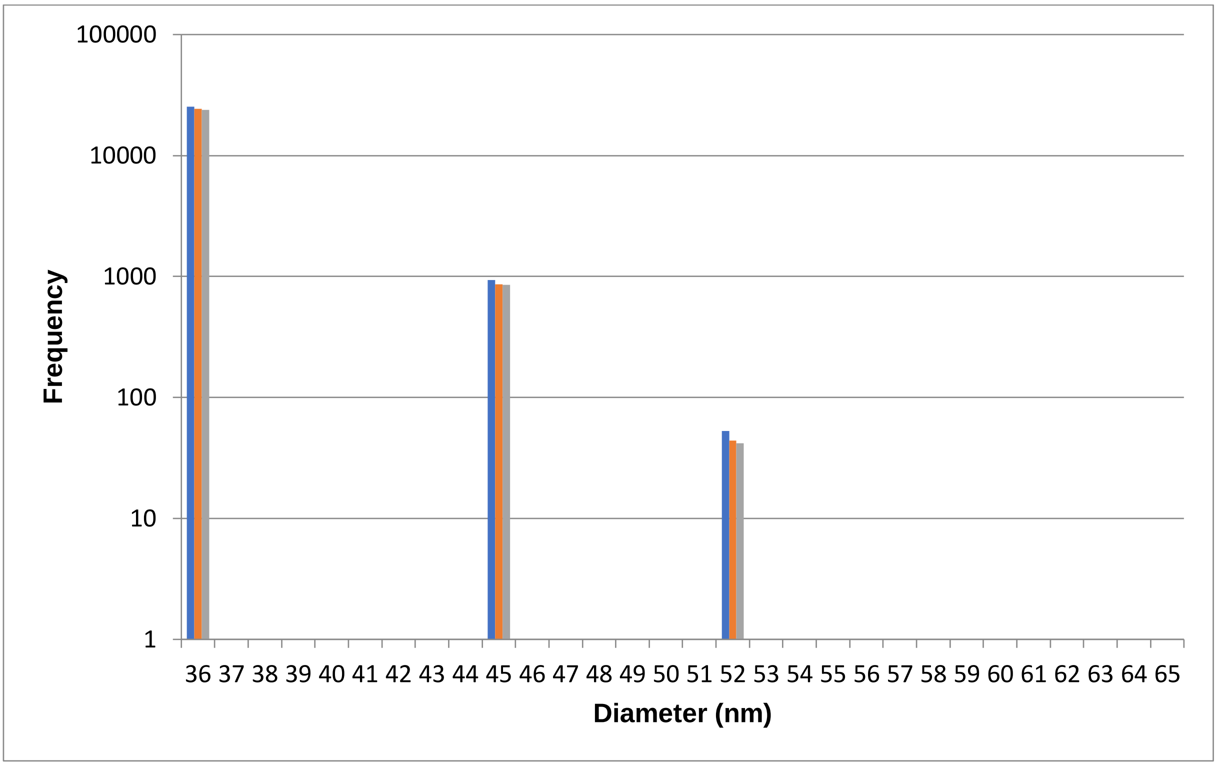

Supplementary figure S41: Size histograms for 3 runs of Lambs lettuce 2 Shoot.

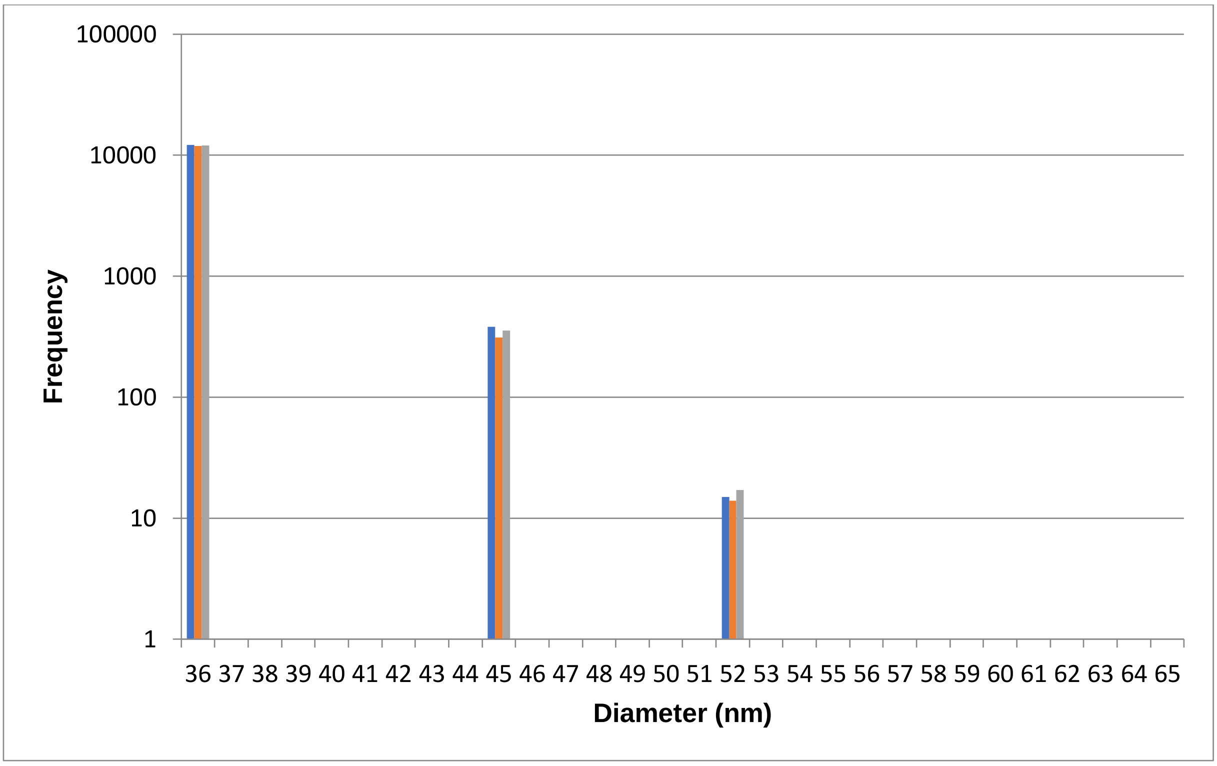

Supplementary figure S42: Size histograms for 3 runs of Lambs lettuce 3 Shoot.

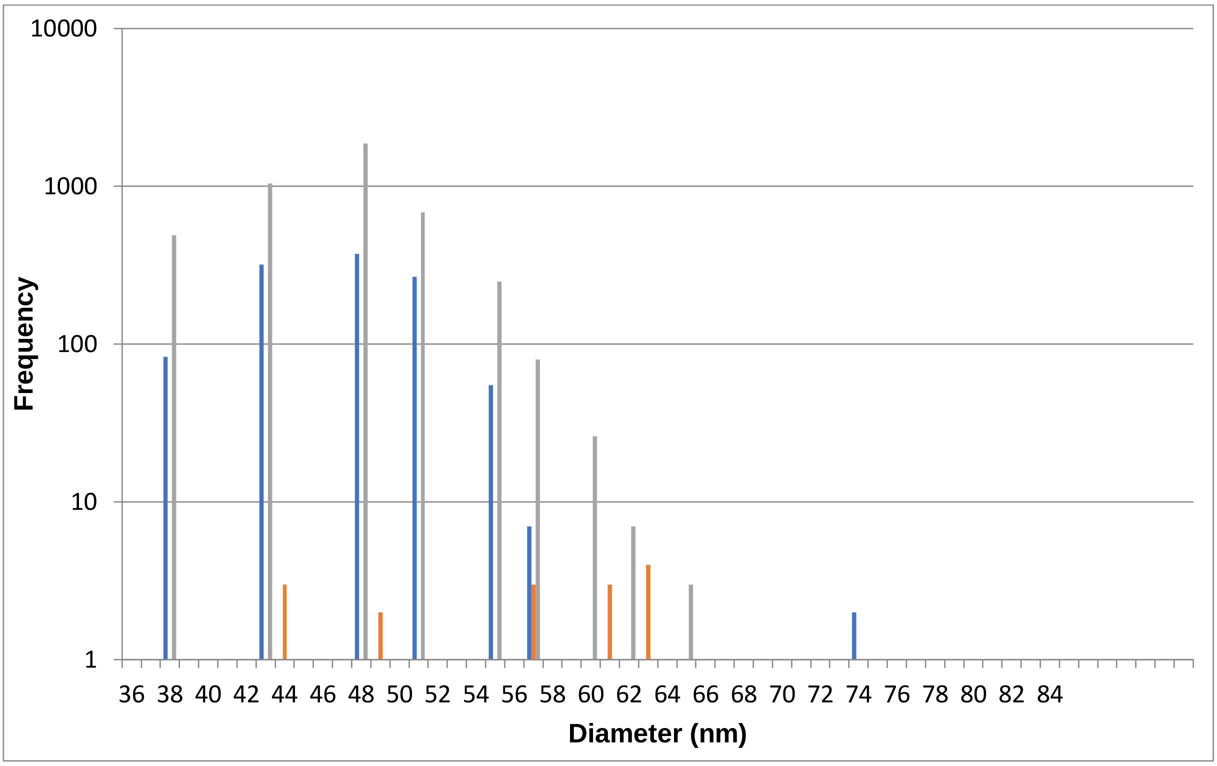

Supplementary figure S43: Size histograms for 3 runs of Brazil Nut 1 Batch 1.

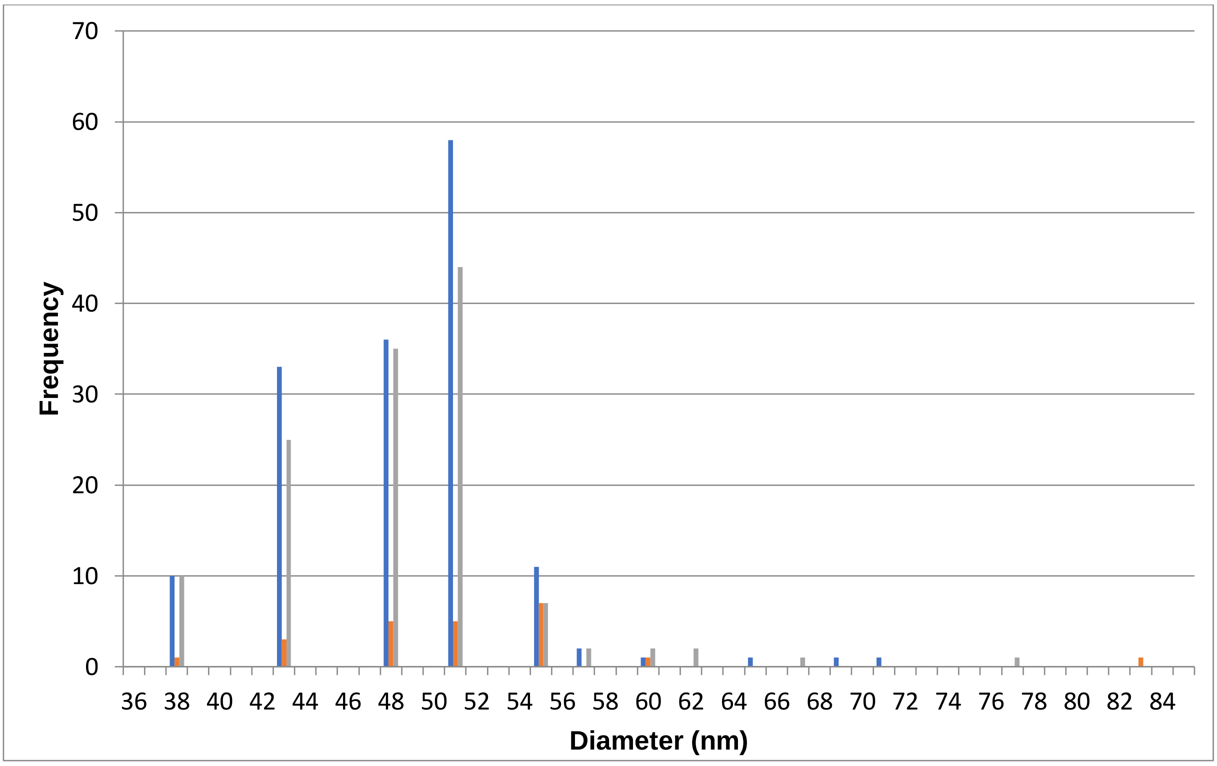

Supplementary figure S44: Size histograms for 3 runs of Brazil Nut 2 Batch 1.

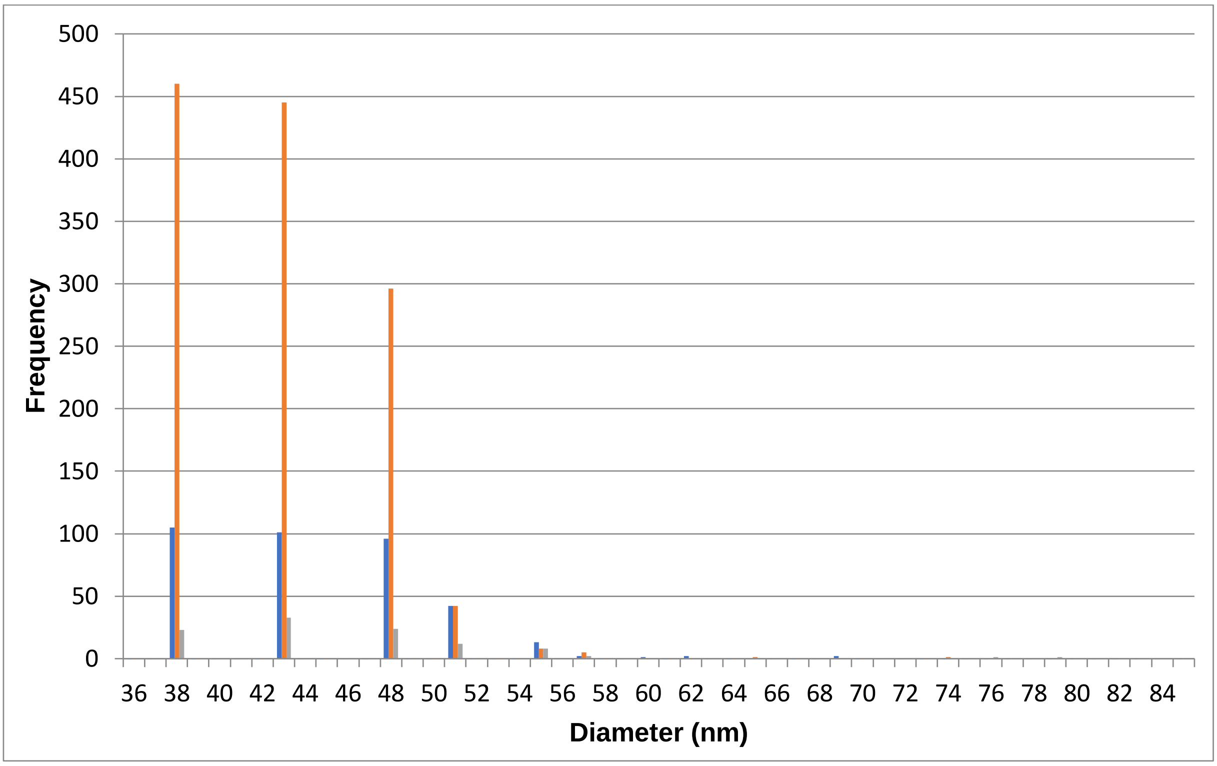

Supplementary figure S45: Size histograms for 3 runs of Brazil Nut 3 Batch 1.

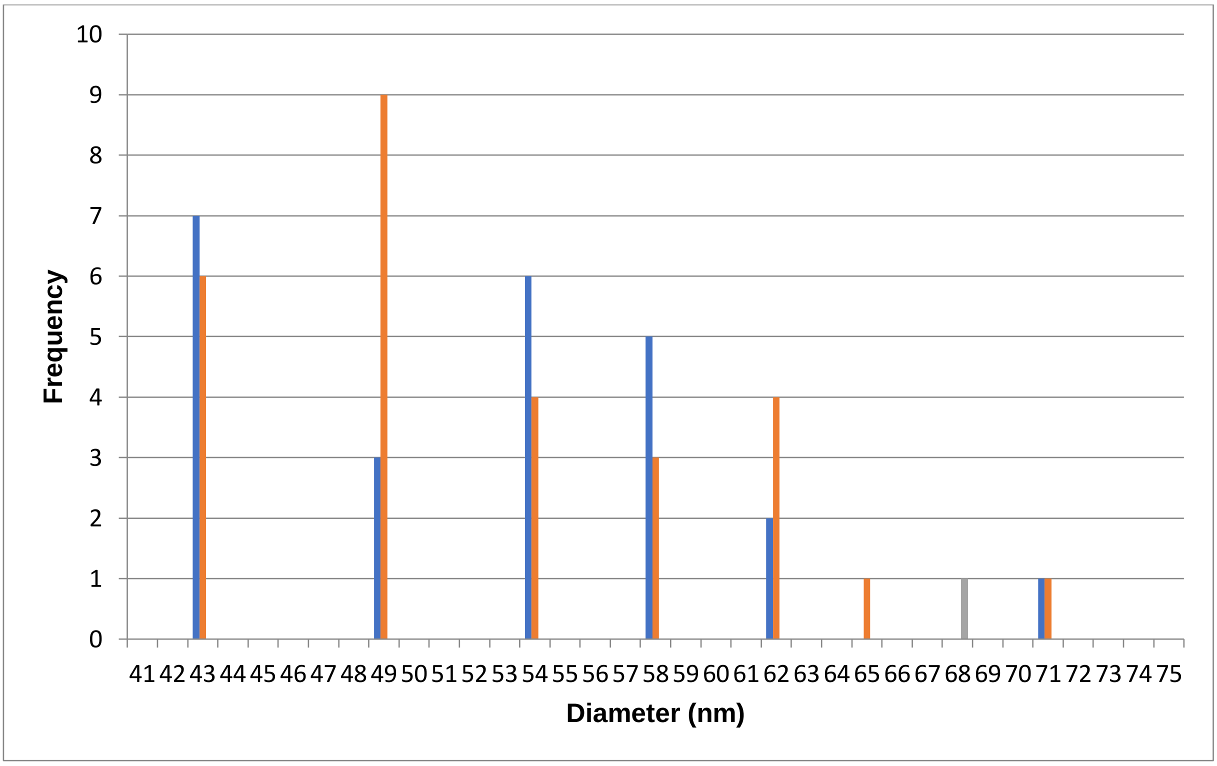

Supplementary figure S46: Size histograms for 3 runs of Brazil Nut 1 Batch 2.

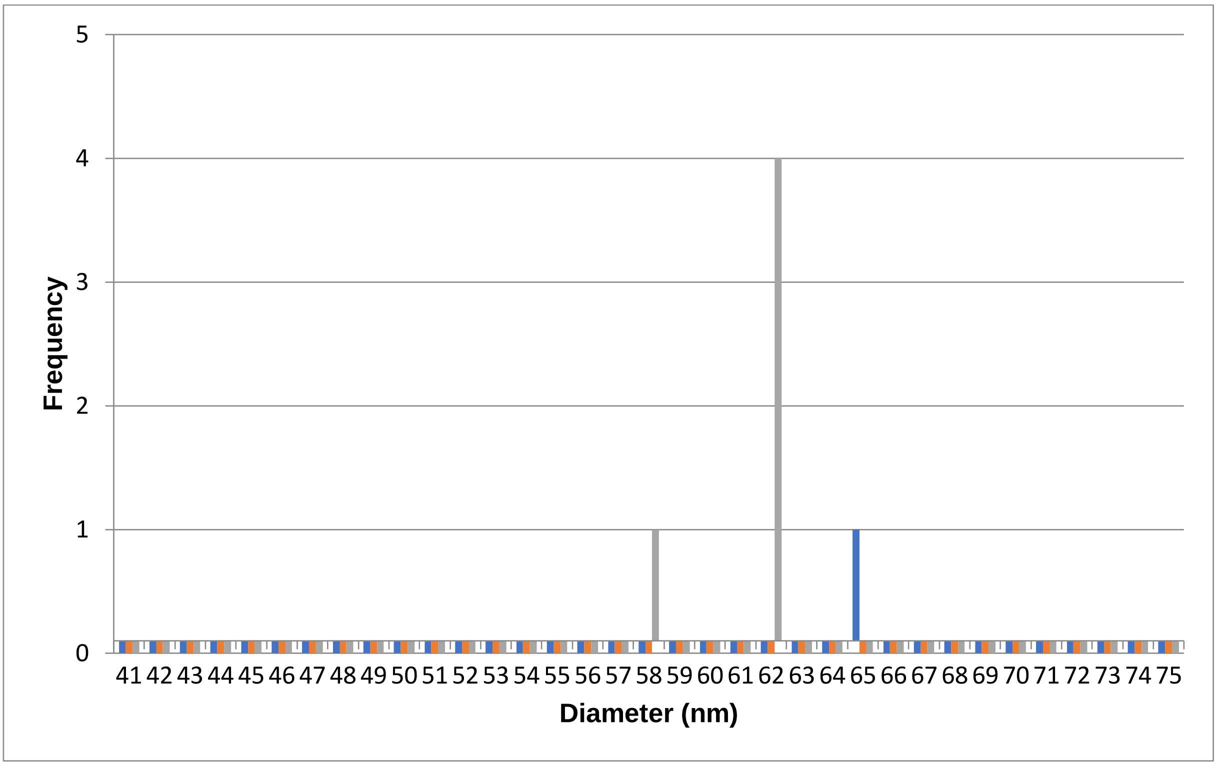

Supplementary figure S47: Size histograms for 3 runs of Brazil Nut 2 Batch 2.

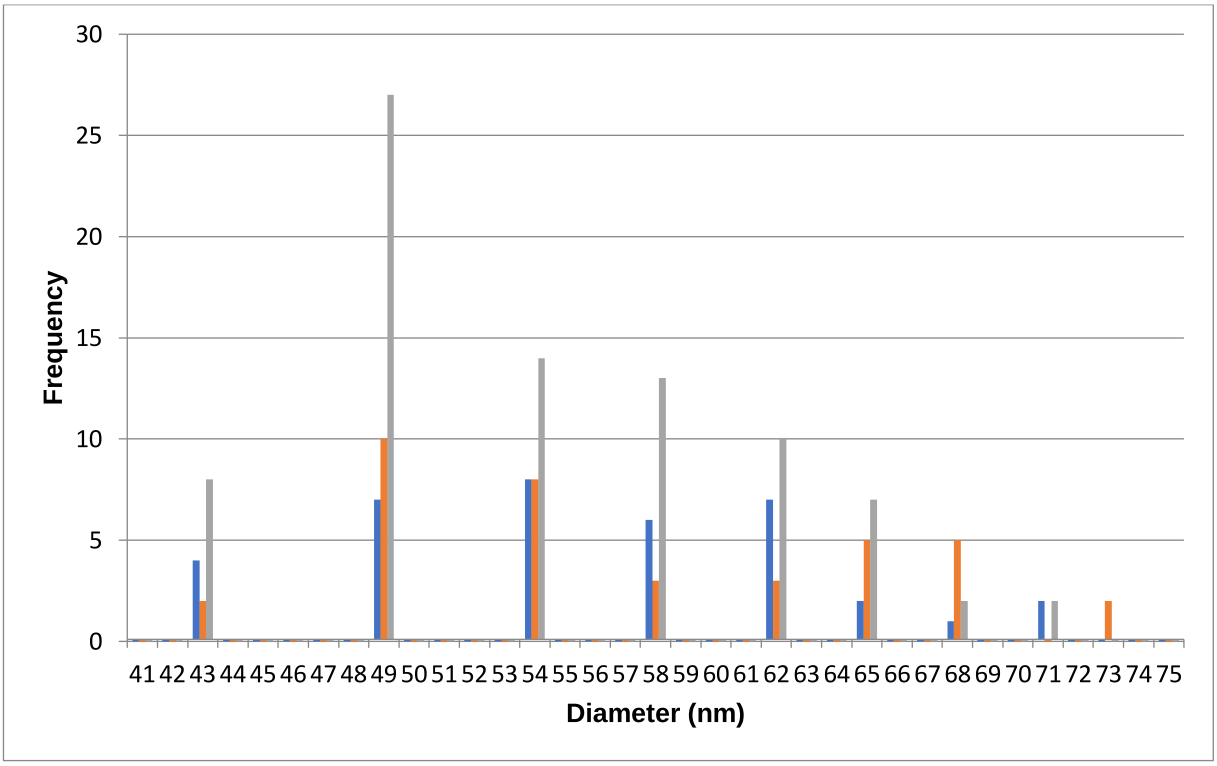

Supplementary figure S48: Size histograms for 3 runs of Brazil Nut 3 Batch 2.
